# CSF1R inhibitors mitigate CDK4/6 inhibitor-induced immunosuppression to increase antitumor immunity in HR+/HER2− breast cancer

**DOI:** 10.1038/s41388-026-03786-w

**Published:** 2026-04-15

**Authors:** Siwei Li, Yajie Gong, Hui Li, Siyu Liu, Yanling Yin, Yuanyuan Yu, Yijun Chu, Jianxun Hou, Jialin Liu, Huaixi Zhang, Abiyasi Nanding, Weiguang Yuan, Youxue Zhang, Qin Wang, Hao Wu, Xianyu Zhang, Da Pang

**Affiliations:** 1 Department of Breast Surgery, Harbin Medical University Cancer Hospital, Harbin, China; 2 Department of Pathology, Harbin Medical University Cancer Hospital, Harbin, China; 3 Institute of Cancer Prevention and Treatment, Harbin Medical University, Harbin, China; 4 Institute of Cancer Prevention and Treatment, Heilongjiang Academy of Medical Sciences, Harbin, China; 5 Department of Scientific Research, Harbin Medical University Cancer Hospital, Harbin, China; 6 Sino-Russian Medical Research Center, Harbin Medical University Cancer Hospital, Harbin, China

**Keywords:** Breast cancer, Immunotherapy, Cancer microenvironment, Cancer immunotherapy

## Abstract

Hormone receptor-positive, human epidermal growth factor receptor 2-negative (HR + /HER2 − ) breast cancer, the most common subtype, shows a low pathological complete response (pCR) rate and limited benefit from immunotherapy, highlighting the need for more effective strategies. Although immunotherapy has become increasingly important in cancer treatment, its efficacy in this subtype remains modest. CDK4/6 inhibitors, first-line treatments for advanced HR + /HER2− breast cancer, not only suppress tumor proliferation but may also reshape the immune microenvironment, offering new opportunities for immunotherapy. In this study, multiplex immunohistochemistry, drug testing of HR + /HER2− breast cancer organoids, single-cell sequencing, and primary cell coculture showed that the CDK4/6 inhibitor palbociclib promotes fibroblast senescence, thereby increasing IGF1 and FGF7 levels. These factors drive macrophage polarization toward an M2-like phenotype through STAT3 Tyr705 phosphorylation and ARG1 upregulation, resulting in arginine depletion and reduced lymphocyte viability. To counteract this immunosuppressive microenvironment, we selected the CSF1R inhibitor pexidartinib. Pexidartinib inhibited macrophage activity, suppressed STAT3 phosphorylation, reduced ARG1 expression, and increased lymphocyte viability, thereby enhancing the antitumor efficacy of palbociclib in HR + /HER2− breast cancer. These findings reveal a previously unrecognized immunosuppressive mechanism induced by CDK4/6 inhibition and support CSF1R blockade as a promising combination strategy.

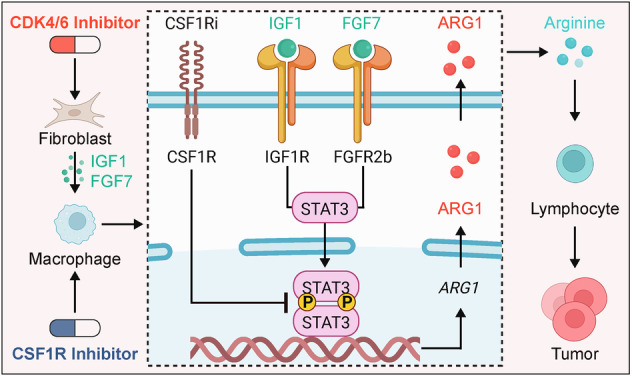

## Introduction

Breast cancer is the most common malignant tumor among women worldwide, and the hormone receptor-positive, human epidermal growth factor receptor 2-negative (HR + /HER2 − ) subtype accounts for approximately 70% of all cases. Currently, surgery remains the cornerstone of treatment for this breast cancer subtype. However, surgical trauma and its associated complications cannot be overlooked. Against this backdrop, pharmacotherapy has gained increasing importance—not only for reducing surgery-related side effects but also for lowering the risk of tumor recurrence, prolonging patient survival, and even enabling some patients to avoid surgery altogether. As such, pharmacological interventions have become a major focus in the treatment of HR + /HER2 breast cancer. Nevertheless, the efficacy of pharmacotherapy in this subtype remains unsatisfactory. The pathological complete response (pCR) rate in HR + /HER2− breast cancer patients receiving neoadjuvant therapy (NAT) is only 3.8 to 18% [1–3], and the 5-year survival rate for patients with postoperative distant metastases is merely 34%, which is significantly lower than that reported for HER2-positive breast cancer (pCR rate: 36–70%) [2, 4] and triple-negative breast cancer (TNBC) patients (pCR rate: ~38%) [2]. These poor outcomes may be attributed to the limitations of current therapeutic strategies. Therefore, optimizing pharmacological treatment strategies for HR + /HER2− breast cancer, particularly in the neoadjuvant and adjuvant settings, to improve treatment response and long-term survival remains an urgent and critical challenge.

Immunotherapy, especially immune checkpoint inhibitors (ICIs), such as PD-L1/PD-1 inhibitors, has achieved significant breakthroughs in cancer treatment in recent years [5]. In triple-negative breast cancer (TNBC), these therapies have demonstrated notable clinical efficacy. However, in HR + /HER2− breast cancer, the therapeutic response has been far less impressive, likely due to the unique characteristics of the immune microenvironment. TNBC is generally linked to elevated levels of tumor-infiltrating lymphocytes (TILs) [6], which promote antitumor immune responses and increase sensitivity to immunotherapy. In contrast, the tumor immune microenvironment (TIME) of HR + /HER2− breast cancer is characterized mostly by myeloid cells, such as immunosuppressive M2-like macrophages, regulatory T cells (Tregs), and other myeloid-derived suppressor cells (MDSCs), which collectively facilitate immune evasion [7, 8]. Furthermore, the enrichment of immunosuppressive cytokines (such as TGF-β and IL-10) and the relatively low expression of PD-L1 in HR + /HER2− tumors further diminish the effectiveness of ICIs [9]. Thus, reprogramming the immunosuppressive TIME to overcome immune tolerance and optimize immunotherapeutic strategies remains a major challenge in both research and clinical practice.

In HR+ breast cancer, estrogen receptor (ER) signaling promotes overexpression of cyclin D proteins, leading to the formation and activation of CDK4/6–cyclin D complexes. These complexes phosphorylate retinoblastoma (Rb) proteins, releasing E2F transcription factors and driving cell cycle progression from G1 to S phase, thereby promoting tumor cell proliferation. CDK4/6 inhibitors block this process by binding to the ATP pocket of CDK4/6, preventing Rb phosphorylation and halting cell cycle progression at the G1 phase [10, 11]. Thus, inhibiting CDK4/6 activity can prevent the proliferation of these cancer cells. Currently, CDK4/6 inhibitors serve as an important therapeutic option for advanced HR + /HER2− breast cancer. Their cooperative effects on ER signaling pathways suggest potential clinical efficacy across multiple stages of disease progression.

Increasing preclinical and clinical data indicate that CDK4/6 inhibitors are related to various immune responses in the peripheral blood, such as enhancing T-cell memory [12, 13] and activation [14], enriching CD4+ and CD137 + CD8 + T cells, and reducing the numbers of Tregs and MDSCs [15, 16], while modulating PD-L1 expression levels on tumor cells [17]. However, there is currently a lack of systematic analysis of the local TIME. Additionally, CDK4/6 inhibitors induce cellular senescence, resulting in the secretion of substantial amounts of senescence-associated secretory phenotype (SASP) factors, including cytokines and growth factors, thereby altering the cellular immune microenvironment [18]. Collectively, these effects suggest that CDK4/6 inhibitors may modulate the TIME. Given that immunotherapy for HR + /HER2− breast cancer is still in its early stages [19], CDK4/6-based immunotherapeutic approaches may offer promising new treatment avenues.

CSF1R is a tyrosine kinase receptor primarily expressed on myeloid cells, including macrophages. When this receptor binds to its main ligand CSF-1 or IL-34, it promotes the survival, proliferation, and differentiation of myeloid cells [20]. The FDA approved a CSF1R inhibitor (pexidartinib, Pex) for the treatment of inoperable tenosynovial giant cell tumors. In breast cancer research, Anita Mehta reported that a CSF1R inhibitor can enhance the therapeutic effect of a PARP inhibitor on BRCA1-associated TNBC [21]; however, specific studies on the use of CSF1R inhibitors in HR + /HER2− breast cancer have not yet been reported.

In this study, we aimed to comprehensively investigate the regulatory effects of CDK4/6 inhibitors on the immune microenvironment of HR + /HER2− breast cancer and to explore potential immunotherapeutic strategies based on these mechanisms. Through multiplex immunohistochemistry (mIHC), drug testing on HR + /HER2− breast cancer organoids, in vitro coculture experiments using primary cells, and single-cell RNA sequencing (scRNA-seq) in mouse models, we demonstrated that the CDK4/6 inhibitor Pal reshaped the immunosuppressive TIME in HR + /HER2− breast cancer. Based on these findings, we selected the CSF1R inhibitor Pex to counteract this effect, thereby increasing Pal's antitumor efficacy in HR + /HER2− breast cancer. Our study suggests that CSF1R inhibitors mitigate CDK4/6 inhibitor-induced immunosuppression to increase antitumor immunity in HR + /HER2− breast cancer.

## Materials and Methods

### Cell culture

The 67NR murine breast cancer cell line (HTX2666, Otwo Biotech, Shenzhen, China), originally derived from BALB/c mice, was cultured in RPMI-1640 medium (PM150110, Procell) supplemented with 10% fetal bovine serum (FBS; 164210-50, Procell) and 1% penicillin–streptomycin (PB180120, Procell) at 37 °C in a humidified atmosphere with 5% CO₂. The cell line was authenticated by the supplier using species-specific PCR to confirm its mouse origin and absence of human, rat, or Chinese hamster cross-contamination, and was routinely tested for mycoplasma contamination before use. THP-1 cells (CL-0233, Procell) were cultured under conditions using RPMI 1640 medium (PM150110, Procell) enriched with 10% FBS (164210-50, Procell), 1% penicillin-streptomycin (PB180120, Procell), and 0.05 mM β-mercaptoethanol. Human primary mammary fibroblasts (HMFs) obtained from Procell (CP-H018) and Bluefcell (HMF7630) were maintained in their specific growth medium (CM-H018, Procell). Primary macrophages derived from human peripheral blood (HPBMs), sourced as CP-H264 from Procell and HPBMC from Bluefcell, were propagated using specialized culture medium CM-H211 (Procell). Human peripheral blood lymphocytes (HPBLs), including Procell’s CP-H211 and Bluefcell’s HPBLC, were grown under controlled conditions with their corresponding medium CM-H211 (Procell).

### Mycoplasma detection

To exclude mycoplasma contamination, newly received or thawed breast cancer cell stocks were quarantined and tested before any experimental use. Subsequently, all cell lines were routinely screened every three months using a standard, commercially available PCR-based detection kit according to the manufacturer’s instructions. For the assay, culture supernatants were collected from cells grown in antibiotic-free medium for at least 48 hours to ensure optimal detection sensitivity. During routine culture, cells were closely monitored for any changes in morphology or growth behavior suggestive of contamination; any suspicious cultures were immediately segregated and retested or discarded. Standard aseptic procedures were strictly adhered to throughout all procedures to minimize cross-contamination between cell lines, ensuring that only cells explicitly confirmed to be mycoplasma-negative were utilized in this study.

### Animal experiments

Female BALB/c mice aged five weeks (14–16 g body weight) were sourced from Beijing Vital River. These animals were maintained under SPF conditions in barrier facilities with controlled 12-hour light/dark cycles, receiving standard laboratory feed and water ad libitum. 67NR cells were suspended in RPMI-1640 medium and orthotopically injected into the secondary mammary fat pad at a density of 8 × 10³ cells per mouse to establish tumors. TCGA dataset evaluation revealed comparable expression patterns of cyclin-dependent kinases 4 and 6 (Cdk4/6) in 67NR cell cultures relative to those observed in murine mammary carcinoma cell lines. Tumor dimensions were assessed using vernier calipers, with neoplastic volume computed according to the formula: volumetric measurement (mm³) = [(longitudinal axis × transverse axis²) × π/6]. On the 12th experimental day following tumor volume attainment of 100 mm³, murine subjects received oral Pal administration. The study was terminated when neoplastic growth exceeded 1000 mm³. Post-euthanasia procedures included comprehensive tumor analysis through scRNA-seq technology, multiplex immunohistochemical evaluation, protein immunoblotting analysis, and cytokine detection via ELISA. Upon reaching a tumor volume of 1000 mm³ in the Veh group on Day 24, the experimental animals were humanely sacrificed, followed by immediate specimen collection. Tumor dimensions were precisely measured prior to necropsy, and tissue samples were systematically harvested for subsequent histological and molecular analyses.

Female BALB/c mice aged five weeks (14–16 g body weight) were sourced from Beijing Vital River Laboratory Animal Technology. The animals were maintained under specific pathogen-free (SPF) conditions with controlled 12-hour light/dark cycles, provided standard rodent chow and sterile water ad libitum. Following acclimatization, 4T1 murine mammary carcinoma cells were resuspended in RPMI-1640 medium and orthotopically injected into the secondary mammary fat pad at a density of 3 × 10⁴ cells per mouse. Tumor dimensions were regularly assessed using precision calipers throughout the study period, with volumetric calculations employing the mathematical formula: Volume (mm³) = (length × width²) × π/6. When tumors reached the predetermined threshold of 100 mm³ on day 12 post-implantation, experimental subjects received Pal compound administration through oral gavage. The investigation concluded on day 24, as per the established protocol.

In animal experiments, palbociclib was dissolved in saline and administered once per day by oral gavage at a dose of 25–75 mg/kg [22–25] (25 mg/kg on days 12–13, 50 mg/kg on days 14–17, and 75 mg/kg on days 18–24). Pexidartinib was dissolved in DMSO and prepared in PEG300 for administration once per day by oral gavage at a dose of 17–50 mg/kg [26–29] (17 mg/kg on days 12–13, 34 mg/kg on days 14–17, and 50 mg/kg on days 18–24). INCB3344 was dissolved in DMSO and prepared in PEG300 for administration once per day by oral gavage at a dose of 10–30 mg/kg [30, 31] (10 mg/kg on days 12–13, 20 mg/kg on days 14–17, and 30 mg/kg on days 18–24). Mice in the corresponding groups were given saline, DMSO, or PEG300 by oral gavage. The interval between each intragastric administration was longer than 2 h.

CDK4/6 inhibitors (such as Pal) and CSF1R inhibitors (such as Pex) may cause gastrointestinal toxicity, hepatic stress, and body weight loss when administered at the full dose from the outset, potentially compromising animal welfare and the feasibility of long-term experiments. Therefore, treatment was initiated at one-third of the target dose, followed by escalation to two-thirds and then to the full dose, allowing the animals to gradually adapt to drug exposure. This stepwise dosing strategy helped reduce acute systemic stress, minimize early mortality, and improve the continuity and reliability of the study. Such dose escalation is commonly used in preclinical pharmacology and toxicology studies to improve tolerability and maintain stable drug exposure over time.

### Human FFPE samples

Three HR + /HER2− breast cancer patients with complete medical histories and intact FFPE sections, who received neoadjuvant therapy with CDK4/6 inhibitors, were identified. Tumor tissue samples from these patients were collected before Pal treatment and 1 month after the final Pal cycle. Since CDK4/6 inhibitors are currently recommended only for advanced breast cancer patients according to guidelines, we selected breast cancer patients who had received CDK4/6 inhibitors as neoadjuvant therapy in recent years because of individualized treatment plans. The samples used in this study were not obtained from a clinical trial. Paraffin sections of patient tumor tissues were subjected to mIHC staining via the PANCK/CD68/CD86/CD206/ARG1 and PANCK/CD4/CD8/FOXP3 protocols. After staining, the immunofluorescence of 5–10 regions per slide was analyzed using Halo and QuPath. The following clinical treatment regimens were used for the three patients: patient A’s regimen consisted of letrozole combined with Pal for eight cycles; Patient B’s regimen consisted of Abraxane combined with carboplatin for two cycles, followed by Abraxane combined with Pal for four cycles; and Patient C’s regimen consisted of epirubicin and cyclophosphamide combined with docetaxel (four cycles), followed by Abraxane combined with Pal for two cycles. Research has indicated that letrozole promotes lymphocyte infiltration in Patient A [32], whereas Abraxane increases both lymphocyte infiltration and polarization of macrophages toward an M1-like phenotype in Patients B and C [33].

### Drug selection

The cell and animal studies utilized several compounds: Palbociclib (S1116, Selleck), Pexidartinib (S7818, Selleck), INCB3344 (S8220, Selleck, USA), Stattic (HY-13818, MCE), Phorbol 12-myristate 13-acetate (PMA, HY-18739, MCE), Lipopolysaccharide (LPS, HY-D1056, MCE), along with various cytokines including human IFN-γ (HY-P7025, MCE), IL-4 (HY-P70764, MCE), IL-13 (HY-P70568, MCE), CSF1 (HY-P701101, MCE), IL-34 (HY-P700125AF), IGF-1 (HY-P7018, MCE), and FGF-7 (HY-P70597). These reagents were specifically selected for their relevance to the experimental protocols.

For cellular assays, palbociclib, LPS, IFN-γ, IL-4, IL-13, IL-34, IGF-1, and FGF-7 were prepared in aqueous solution. Pexidartinib, stattic, PMA, and CSF1 were reconstituted using dimethyl sulfoxide. THP-1 monocytes were exposed to 100 ng/mL PMA for 24 hours to differentiate into adherent macrophages. These differentiated cells subsequently received 100 ng/mL LPS combined with 20 ng/mL IFN-γ over 48 h to drive M1 polarization. To induce M2 polarization, macrophages were stimulated with 20 ng/mL IL-4 and 20 ng/mL IL-13 for 48 h. Subsequently, THP-1 cell cultures were supplemented with 50 ng/ml IGF1 paired with 50 ng/ml FGF7 for equivalent duration. Additional experimental groups underwent treatment protocols involving 50 ng/ml CSF1, 50 ng/ml IL-34, and 5 μM Pex administered concurrently over two days in controlled culture conditions.

### β-Galactosidase staining

Cellular senescence detection was accomplished using β-galactosidase activity test (KTA3030, Abbkine). Following treatment, cells were rinsed with PBS before fixation with the fixative solution. After removing the fixative, samples were subsequently rinsed again with PBS and immersed in staining mixture. The prepared specimens were maintained in a CO2-free incubation chamber at 37 °C for a 1–48-hour period. Cellular morphology and staining patterns were subsequently analyzed through bright-field microscopy observation. The quantities of both blue-stained cells and the overall cell population were enumerated, and the proportion of β-galactosidase-positive cells (indicative of senescence) was determined.

### Cell viability assay

After plating 2000–5000 cells per well into 96-well plates and allowing them to adhere, the cells were kept in drug-supplemented growth media for two days at 37 °C under carefully monitored conditions. Following medium exchange with CCK-containing solution, cellular samples underwent 1–2 h of incubation prior to optical density measurement using an absorbance reader.

### ELISA

ELISA kits were acquired from Neobioscience (EMC113QT, EMC010a), MEIMIAN (4199, 3946, 3944), and ENZO (E10482). Cellular supernatants and tumor tissue lysates were processed following manufacturers’ protocols. Optical density measurements were performed using a microplate reader, with protein concentrations determined through standard curve interpolation. Final values were adjusted against total protein content determined by BCA assay for comparison analysis.

### Western blot

Protein isolation was conducted using RIPA lysis buffer (R0010, Solarbio) including PMSF protease inhibitor (P0100, Solarbio) and PhosSTOP phosphatase inhibitor cocktail (4906837001, Roche). Electrophoretic separation of proteins was achieved through SDS-PAGE, followed by immunoblotting analysis with specific rabbit-derived antibodies targeting human/mouse epitopes. Quantitative analysis involved measuring band intensities through densitometry, which were subsequently quantified relative to the corresponding loading control. Comprehensive details regarding the antibodies utilized are available in the Western blot Extended Data section.

### Organoids

Breast cancer specimens obtained from clinical cases were initially immersed in pre-chilled phosphate-buffered saline, then finely sectioned and enzymatically treated to generate three-dimensional tumor models. The isolated cellular components were subsequently reconstituted in specialized tumoroid growth medium and maintained at 4 °C. During culture, 100 μl aliquots of the cell-containing extracellular matrix hydrogel mixture were seeded into 12-well plates and overlaid with 500 μl of tumoroid maintenance medium, which was replaced every 2 days under aseptic conditions. For processing mature organoids in 12-well plates, enzymatic dissociation was performed using a specialized digestion solution to obtain single-cell suspensions. Mechanical disruption was achieved by pipetting matrix gels using 1-ml tips while preventing bubble formation. Microscopic examination was conducted to assess suspension quality before proceeding. Subsequent procedures included gentle scraping of matrix gels from culture surfaces, followed by precise pipetting techniques to preserve breast cancer organoid integrity during transfer. For cryopreservation procedures, organoids were chilled on ice for 5–10 min before undergoing centrifugation. After discarding the supernatant, the cellular pellet underwent resuspension in 1 mL of cryoprotective solution. This homogeneous mixture was subsequently aliquoted into specialized freezing vials, which were stored at −80 °C in temperature-controlled storage containers for 24 h. Following this initial freezing phase, samples were permanently archived in liquid nitrogen storage systems.

### Assessment of organoid viability and cytotoxicity (ATP and LDH Assays)

Organoid viability was assessed by measuring intracellular ATP levels and extracellular LDH release. After drug treatment for the indicated time, organoids were collected and gently dissociated to ensure uniform exposure. For ATP detection, organoids were lysed, and the luminescence signal corresponding to ATP content was measured using a microplate reader. ATP levels were used to reflect cellular metabolic activity. For LDH measurement, the culture supernatant was collected, and the enzymatic reaction between released LDH and the substrate mixture was allowed to proceed at room temperature. The absorbance was then read at 490 nm, and the percentage of LDH release was calculated relative to total LDH, indicating the degree of cell membrane damage. All measurements were performed in triplicate, and results were expressed as mean ± standard deviation.

### Single-cell RNA-seq

The tumors of BALB/c mice in the vehicle (Veh) group (three samples, 15397 cells), palbociclib (Pal) group (four samples, 20895 cells), pexidartinib (Pex) group (three samples, 17484 cells), and palbociclib combined with pexidartinib (Pal+Pex, 25727 cells) group (four samples) were tested. For each sample, two random tumors from within the group were mixed. The data were analyzed on the Majorbio Cloud Platform (www.majorbio.com) [34] by Shanghai Majorbio Biopharm Technology Co., Ltd. The number of captured cells for each sample ranged from 4259 to 7020, and the median number of genes per cell ranged from 2194 to 4755. The percentage of effective barcodes ranged from 97.8 to 98.4%. The sequencing capacity for each sample was 120 G. See the Supplementary Data scRNA-seq for details.

Cell-cycle phase assignment was performed using single-cell RNA-sequencing data. This method is based on the characteristic gene-expression signatures of different cell-cycle phases (G1, S, and G2/M). Because mitotic cells undergo substantial transcriptomic variation during the transition between S and G2/M phases, which can mask underlying biological signals, we calculated phase-specific scores (G1, S, and G2/M) for each cell to infer its likely cell-cycle state. Our single-cell analyses were performed using the Majorbio Cloud Platform (https://www.majorbio.com/tools).

### Cell cycle analysis

Cell-cycle distribution was determined by flow cytometry. Treated cells were washed twice with PBS and fixed in 70% pre-chilled ethanol at 4 °C overnight. After fixation, cells were washed with PBS, incubated with 50 μg/mL RNase A (Thermo Fisher Scientific) at 37 °C for 30 min to remove RNA, and then stained with 50 μg/mL propidium iodide (PI; Sigma-Aldrich) for 30 min in the dark. Samples were analyzed on a BD FACSCanto II flow cytometer, and at least 10,000 events per sample were collected. Data were processed using FlowJo software (v10.8, BD Biosciences) to determine the percentage of cells in G0/G1, S, and G2/M phases. Experiments were performed at least three times independently, and data are expressed as mean ± standard deviation (SD); statistical significance was evaluated using a two-tailed Student’s t-test or one-way ANOVA.

### Communication analysis

AUCell, UMAP, GSEA, GO, KEGG, and cell cycle analyses were conducted using the Majorbio Cloud Platform (https://cloud.majorbio.com/page/tools/).

### GEO analysis

We downloaded breast cancer cell line gene expression data for BALB/c mice from the GSE11259, GSE160101, GSE226910 and GSE42272 datasets. Gene expression in 67NR cells was subsequently normalized.

### Confocal microscopy

Breast cancer organoids were cultured and subsequently fixed onto glass slides, followed by EdU staining to assess cellular proliferation. Imaging was performed using high-resolution confocal microscopy to capture detailed structural and proliferation data within the organoids. The acquired confocal images were then processed and analyzed using Fiji software, which allowed for precise quantification and visualization of cell proliferation markers (https://fiji.sc).

### Statistical analysis

Statistical analyses were conducted via GraphPad Prism software version 10. For the comparison of data from two groups, the t test, Welch’s t test, or Wilcoxon rank sum test was chosen for the analysis. For the comparison of data from multiple groups, one-way ANOVA, Welch’s one-way ANOVA, or the Kruskal‒Wallis test was used. *P* < 0.05 was regarded as indicating statistical significance for all experiments. The following symbols represent the degrees of significance: *P < *0.05*; **P < *0.01*; ***P < *0.001*.

## Results

### Pal promotes M2-like macrophage polarization and upregulates Arg1 expression

**Fig. 1:**
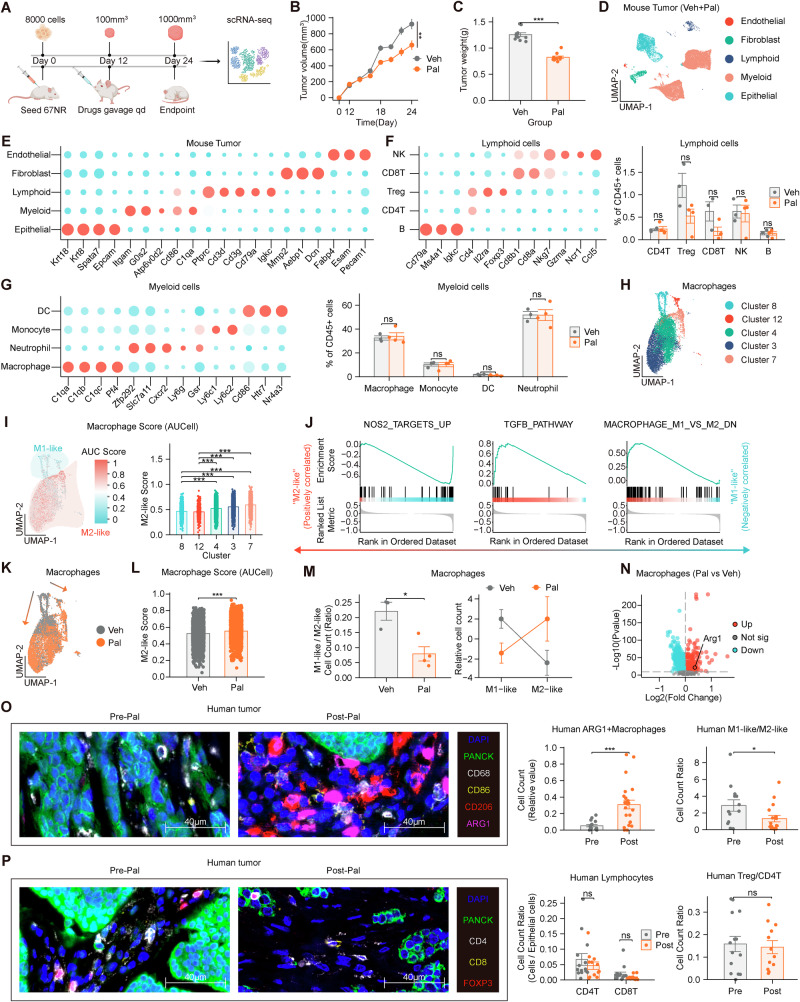
Pal promotes M2-like macrophage polarization and upregulates Arg1 expression. **A** ER + 67NR breast cancer cells derived from BALB/c mice were orthotopically implanted into 20 female BALB/c mice. **B** Pal reduced the tumor volume in the mice (Veh: *n* = 10; Pal: *n* = 10). **C** Pal reduced the tumor weight in the mice (Veh: *n* = 10; Pal: *n* = 10). **D**, **E** Cell clustering annotation of mouse tumor tissues. **F** Pal did not affect the number of lymphoid cells. **G** Pal did not affect the number of myeloid cells. **H** Macrophage UMAP plot. **I** Macrophage AUCell analysis of M2-like cells (*n* = 10621). **J** GSEA of the NOS2, TGFB, and MACROPHAGE_M1_VS_M2 gene sets revealed that the macrophages in Clusters 3, 4, and 7 were similar to M2-like macrophages. **K**–**M** UMAP, AUCell and cell count analyses revealed the polarization of macrophages toward M2-like macrophages after Pal treatment (*n* = 10621). **N** Pal promoted the expression of Arg1 in macrophages. **O** Pal increased the number of ARG1+ macrophages. Pal decreased the number of M1-like macrophages (PANCK − /CD68 + /CD86 + ) and increased the number of M2-like macrophages (PANCK − /CD68 + /CD86 − /CD206 + ) (Pre: *n* = 3; Post: *n* = 3). **P** No significant differences in the numbers of CD4T (PANCK − /CD4 + /CD8 − /FOXP3-), CD8T (PANCK − /CD4 − /CD8 + /FOXP3 − ), or Tregs (PANCK − /CD4 + /CD8 − /FOXP3 + ) were observed (Pre: *n* = 3; Post: n = 3). mIHC: multiplex immunohistochemistry. Veh: vehicle, Pal: palbociclib. The data are shown as the means ± SEMs. *P < 0.05, ***P < 0.001, ns: not significant, Wilcoxon rank sum test, Kruskal–Wallis test, two-way repeated-measures ANOVA and t test.

We initially investigated the potential of the CDK4/6 inhibitor Pal to modulate the TIME of HR + /HER2− breast cancer. To further explore its impact on the TIME in vivo, we performed scRNA-seq on breast tumors from mice. Twenty female BALB/c mice were orthotopically implanted with 67NR mouse breast cancer cells that were ER-positive [35, 36] (Fig. 1A and Supplementary Fig. S1A). We assessed the expression of ER α (ERα) in 67NR cells and found that 67NR cells express ERα at a lower level than the ER-positive control MCF7, but markedly higher than the ER-negative cell lines MDA-MB-231, 4T1, and EMT6 (Supplementary Fig. S1B). The data regarding tumor volume and mass indicated that Pal suppressed tumor growth in the mice (Fig. 1B, C). Initially, the cells were categorized as epithelial, fibroblast, endothelial, myeloid, or lymphoid cells (Fig. 1D–E, Supplementary Fig. S2). Lymphoid cells were divided into CD4 + T cells (CD4T), CD8 + T cells (CD8T), Tregs, natural killer (NK) cells, and B lymphocytes (B), no significant differences were observed between the Veh and Pal groups (Fig. 1F, Supplementary Fig. S3). The myeloid cells were further divided into neutrophils, macrophages, dendritic cells (DCs), and monocytes; no significant differences were observed between the Veh and Pal groups (Fig. 1G, Supplementary Fig. S4). We performed flow cytometric analysis of murine tumor tissues and found that the numbers of CD4⁺ and CD8⁺ T cells within the TIME showed no marked changes following Pal treatment (Supplementary Fig. S1C). This finding contrasts with existing studies reporting that CDK4/6 inhibitors can increase the numbers of CD4 + T cells and CD8 + T cells in the blood [15, 16]. The observed effect may be attributed to the immunosuppressive actions of CDK4/6 inhibitors within the TIME.

The macrophage UMAP plot revealed that the macrophages consisted of Clusters 3, 4, 7, 8, and 12 (Fig. 1H). According to the AUCell score analysis, the cells with higher scores (represented in red) resembled M2-like macrophages, whereas the cells with lower scores (represented in blue) resembled M1-like macrophages (Fig. 1I, Supplementary Fig. S5A). GSEA of the NOS2, TGFB, and MACROPHAGE_M1_VS_M2 gene sets revealed that the macrophages in Clusters 3, 4, and 7 were similar to M2-like macrophages (Fig. 1J), and CXCR4 and PDGF were also enriched in M2-like macrophages (Supplementary Fig. S6A, B). UMAP, AUCell and cell count analyses revealed the polarization of macrophages toward M2-like phenotypes after Pal interference (Fig. 1K–M, Supplementary Fig. S5B). Cell cycle analysis and GSEA revealed that Pal induced cell cycle arrest in M1-like macrophages (Supplementary Figure S6C–H). Since Pal treatment markedly affected macrophage populations within the TIME, we further analyzed the expression of key macrophage functional genes, including ARG1, NOS2, CCL, IL10, TGFB, CD163, and CD206. *Arg1* expression in macrophages was upregulated at the transcriptomic level (Fig. 1N).

To validate the findings from the animal experiments, we performed mIHC staining in triplicate on FFPE samples. Samples from HR + /HER2− breast cancer patients receiving neoadjuvant therapy, including Pal, were subjected to mIHC staining (Supplementary Fig. S7A). mIHC revealed that post-Pal neoadjuvant therapy led to an increase in the number of ARG1+ macrophages, an increase in the number of M2-like macrophages (PANCK − /CD68 + /CD86 − /CD206 + ), and a reduction in the number of M1-like macrophages (PANCK − /CD68 + /CD86 + ) (Fig. 1O, Supplementary Fig. S7D, E). There were no significant changes in the numbers of CD4-positive T cells (CD4T) (PANCK − /CD4 + /CD8 − /FOXP3 − ), CD8-positive T cells (CD8T) (PANCK − /CD4 − /CD8 + /FOXP3 − ), or regulatory T cells (Tregs) (PANCK − /CD4 + /CD8 − /FOXP3 + ) (Fig. 1P, Supplementary Fig. S7F, G). These findings were further confirmed by flow cytometry analysis of human tumor tissues (Supplementary Fig. S8). Therefore, we hypothesize that the CDK4/6 inhibitor Pal may inhibit lymphocytes in the TIME by promoting macrophage polarization toward an M2-like phenotype.

### Pal attenuates lymphocyte communication within the TIME and reduces Ki-67 expression in T and NK cells

**Fig. 2:**
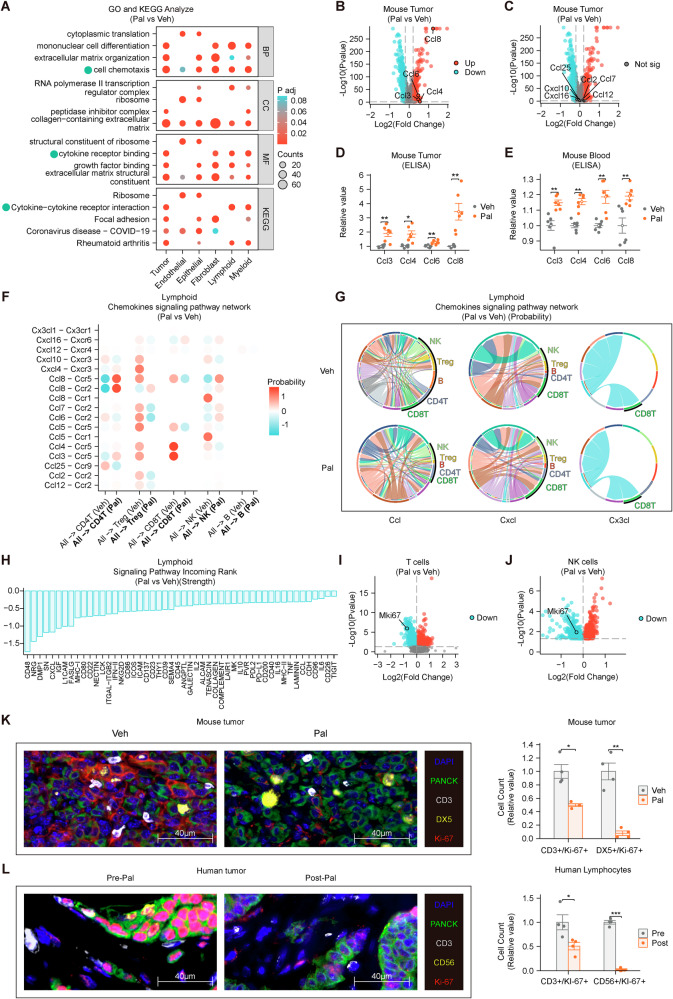
Pal attenuates lymphocyte communication within the TIME and reduces Ki-67 expression in T and NK cells. **A** GO and KEGG enrichment analyses of the mouse tumor tissue transcriptome revealed multiple pathways related to chemokines (indicated by green dots). **B**, **C** Pal induces the secretion of Ccl3, Ccl4, Ccl6, and Ccl8 in mouse tumors (n = 9879). **D** ELISA detection of Ccl in mouse tumor tissues. **E** ELISA detection of Ccl in the peripheral blood of the mice. **F**, **G** The probability of communication between chemokines and lymphoid cells did not increase significantly. **H** The strength of most communication related to lymphoid activation decreased. **I** Transcriptome analysis revealed that the expression of Mki67 is decreased in T cells. **J** Transcriptome analysis revealed that the expression of Mki67 is decreased in NK cells. **K** The expression of Ki-67 in T and NK cells was reduced, as demonstrated by the mIHC assay (Veh: *n* = 3; Pal: *n* = 3). **L** The expression of Ki-67 was decreased in CD3-positive T cells (CD3T) (PANCK − /CD3 + /CD56 − ) and NK cells (PANCK − /CD3 − /CD56 + ) (Pre: *n* = 3; Post: *n* = 3). mIHC: multiplex immunohistochemistry. Veh: vehicle, Pal: palbociclib, TIME: tumor immune microenvironment. The data are shown as the means ± SEMs. *P < 0.05, **P < 0.01, ***P < 0.001, Wilcoxon rank sum test and Welch’s t test.

Based on a preliminary analysis of immune cell numbers in the TIME of mice, we decided to further analyze immune cell function using scRNA-seq data. Transcriptomic analysis using GO and KEGG methodologies revealed significant enrichment of chemokine-associated signaling pathways (marked by green indicators) in tumor tissue samples (Fig. 2A, Supplementary Fig. S9). Transcriptome analysis of tumor tissues revealed that Pal intervention promoted the expression of various chemokines (*Ccl3*, *Ccl4*, *Ccl6*, and *Ccl8*) (Fig. 2B, C). These ligands are primarily secreted by myeloid cells within the tumor, including *Ccl3*, *Ccl4*, *Ccl6*, and *Ccl8* (Supplementary Fig. S10A). The detection of mouse tumors and blood indicated that the expression of Ccl3, Ccl4, Ccl6, and Ccl8 increased after Pal interference (Fig. 2D, E). Studies have shown that Ccl3, Ccl4, Ccl6, and Ccl8 can promote the chemotaxis of lymphocytes [37]. Therefore, Pal should increase lymphocyte accumulation within the tumor. However, we employed CellPhoneDB to analyze the probability of communication (the likelihood of observing receptor‒ligand interactions between two cell types) and the strength of communication (influenced by the expression levels of receptors and ligands, reflecting the intensity or impact of signal transmission once communication occurs). The probability of communication between chemokines and lymphoid cells did not increase significantly (Fig. 2F–G). The strength of most communication related to lymphoid activation decreased (Fig. 2H, Supplementary Fig. S10B). The probability of communication related to lymphoid activation (MHC-II, CD80, FASLG and CD86) decreased (Supplementary Figs. S11–14), and the changes in the communication probability for MHC-I and ICOS were not significant (Supplementary Figs. S15–16). Transcriptome analysis revealed that the expression of *Cd28* in CD4 + T cells and that of *Klrb1c* in NK cells was downregulated (Supplementary Fig. S10C, D). Transcriptome analysis revealed that the expression of *Mki67* was decreased in T and NK cells (Fig. 2I–J). The expression of Ki-67 in T and NK cells was reduced, as demonstrated by mIHC in mouse and human tumors (Fig. 2K–L), and this finding was validated by flow cytometry (Supplementary Fig. S17). Thus, we considered the possibility that Pal promotes macrophage polarization toward the M2-like phenotype and Arg1 expression, affecting lymphocytes and thereby creating an immunosuppressive TIME.

### Pex inhibits macrophage infiltration and promotes lymphocyte infiltration within tumors

**Fig. 3:**
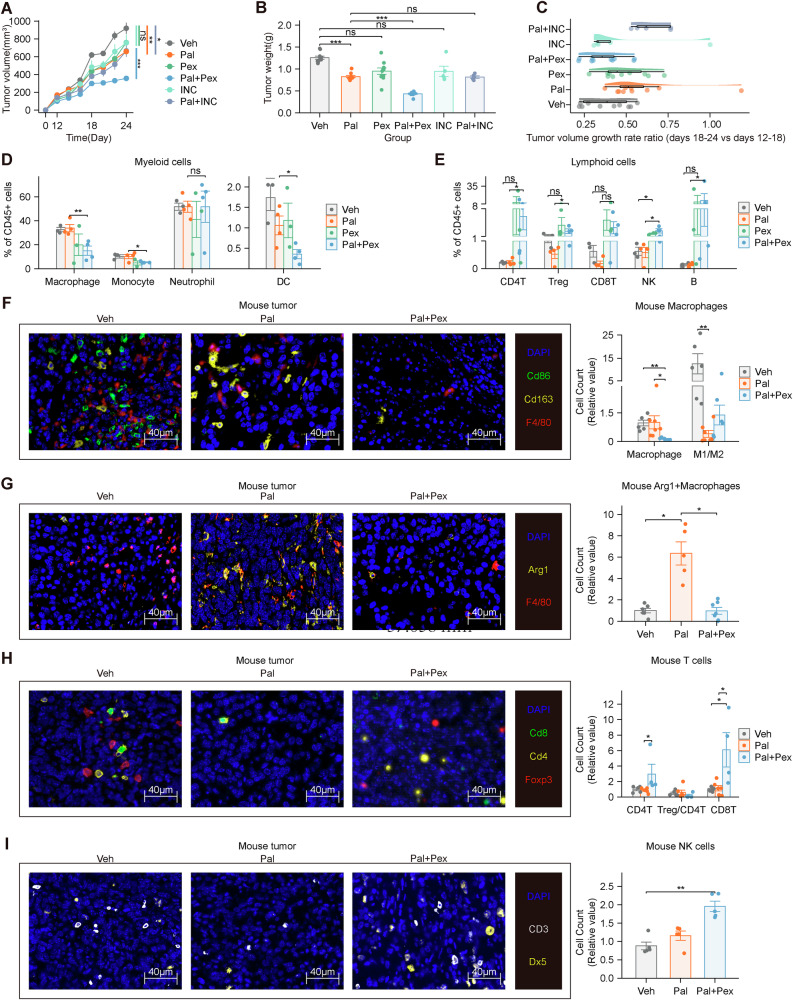
Pex inhibits macrophage infiltration and promotes lymphocyte infiltration within tumors. **A**, **B** Pex enhanced the killing effect of Pal on mouse tumors, whereas INC did not enhance the tumor-killing effect of Pal (Veh, Pal, Pex and Pal+Pex: *n* = 10; INC and Pal+INC: *n* = 5). **C** The tumor growth rate ratio of mouse tumors (Veh, Pal, Pex and Pal+Pex: *n* = 10; INC and Pal+INC: *n* = 10). **D** Pex intervention reduced the number of myeloid cells (*n* = 14). **E** Pex intervention increased the number of lymphoid cells (*n* = 14). **F** Pex decreased the number of macrophages (F4/80 + ) within the tumor according to mIHC (Veh: *n* = 3; Pal: *n* = 3; Pal+Pex: *n* = 3). **G** Pex decreased the number of Arg1+ macrophages (F4/80 + /Arg1 + ) within the tumor according to mIHC (Veh: *n* = 3; Pal: *n* = 3; Pal+Pex: *n* = 3). **H** Pex increased the number of CD4 + T cells (Cd4 + /Cd8 − /Foxp3-) and CD8 + T cells (Cd4 − /Cd8 + /Foxp3 − ) within the tumor according to mIHC (Veh: *n* = 3; Pal: *n* = 3; Pal+Pex: *n* = 3). **I** Pex increased the number of NK cells (CD3 − /Dx5 + ) within the tumor according to mIHC (Veh: *n* = 3; Pal: *n* = 3; Pal+Pex: *n* = 3). Veh: vehicle, Pal: palbociclib, Pal+Pex: palbociclib+pexidartinib. The data are shown as the means ± SEMs. *P < 0.05, **P < 0.01, ns: not significant, one-way ANOVA, Kruskal–Wallis test, two-way repeated-measures ANOVA and Welch’s one-way ANOVA.

On the basis of previous experimental results, we administered targeted immunotherapy drugs for macrophages, the CSF1R inhibitor Pex and the CCR2 inhibitor INCB3344 (INC), to mice. Pex primarily inhibits the proliferation and survival of macrophages through CSF1R, whereas CCR2 inhibitors mainly inhibit the migration of macrophages. The mouse tumors were divided into four groups—Veh, Pal, Pex, and Pal combined with Pex (Pal+Pex)—and treated accordingly. Experiments with 67NR mouse tumors revealed that the effects of Pex and INC monotherapy were not significantly different from those of Veh. A significant difference was not observed between the combination of Pal and INC or Pal alone. The tumoricidal efficacy of Pal+Pex surpassed that of Pal monotherapy (Fig. 3A, B). Compared with that in the first week (days 12–18), tumor growth accelerated in the second week (days 18–24) in both the Pal and Pal+INC groups, whereas Pex inhibited accelerated tumor growth in the Pal group (Fig. 3C). Experiments using the mouse 4T1 tumor model and genetically engineered Stat − /− C57BL/6 mice yielded similar results (Supplementary Figs. S18 and S1A). These results suggest that Pal may induce immune tolerance within the tumor, whereas Pex counteracts this mechanism, increasing antitumor efficacy.

Subsequent scRNA-seq of mouse tumors across all groups, which were analyzed on the basis of the cell classification annotations in Fig. 1 (Supplementary Fig. S19A–C), revealed that Pex intervention reduced the infiltration of macrophages, DCs, and monocytes in the TIME but had no significant effect on neutrophils (Fig. 3D and Supplementary Fig. S19D). Pex intervention increased the numbers of CD4 + T cells, Tregs, NK cells, and B cells in the tumors (Fig. 3E and Supplementary Fig. S19E). Mouse tumor tissues were subjected to mIHC staining. Compared with those in the Veh group, a decrease in the number of M1-like macrophages (F4/80 + /Cd86 + /Cd163 − ) and an increase in the numbers of M2-like macrophages (F4/80 + /Cd86 − /Cd163 + ) and Arg+ macrophages (F4/80 + /Arg1 + ) were detected in the Pal group, whereas the Pal+Pex group showed a decrease in macrophage numbers (Fig. 3F–G). Compared with those in the Veh group, the numbers of CD4 + T cells (Cd4 + /Cd8 − /Foxp3-), Tregs (Cd4 + /Cd8 − /Foxp3 + ), CD8 + T cells (Cd4 − /Cd8 + /Foxp3 − ), and NK cells (CD3 − /Dx5 + ) did not change significantly in the Pal group, but increases in the numbers of CD4 + T cells, CD8 + T cells and NK cells were observed in the Pal+Pex group (Fig. 3H–I), which was further confirmed by flow cytometry analysis of mouse tumor tissues (Supplementary Fig. S20). The consistency between protein data from mIHC staining of mouse tumor FFPE samples and transcriptomic data from scRNA-seq of mouse tumors, along with their similarity to mIHC results from breast cancer patients, suggests a strong correlation. Additionally, Pex intervention resulted in a decrease in macrophages and an increase in lymphocytes within the TIME, indicating that Pex enhanced the therapeutic effects of Pal. Next, we investigated the underlying mechanisms involved.

### Pex reduces Arg1 expression and is associated with attenuation of CD8^+^ T cell exhaustion and Treg-mediated immunosuppression

**Fig. 4:**
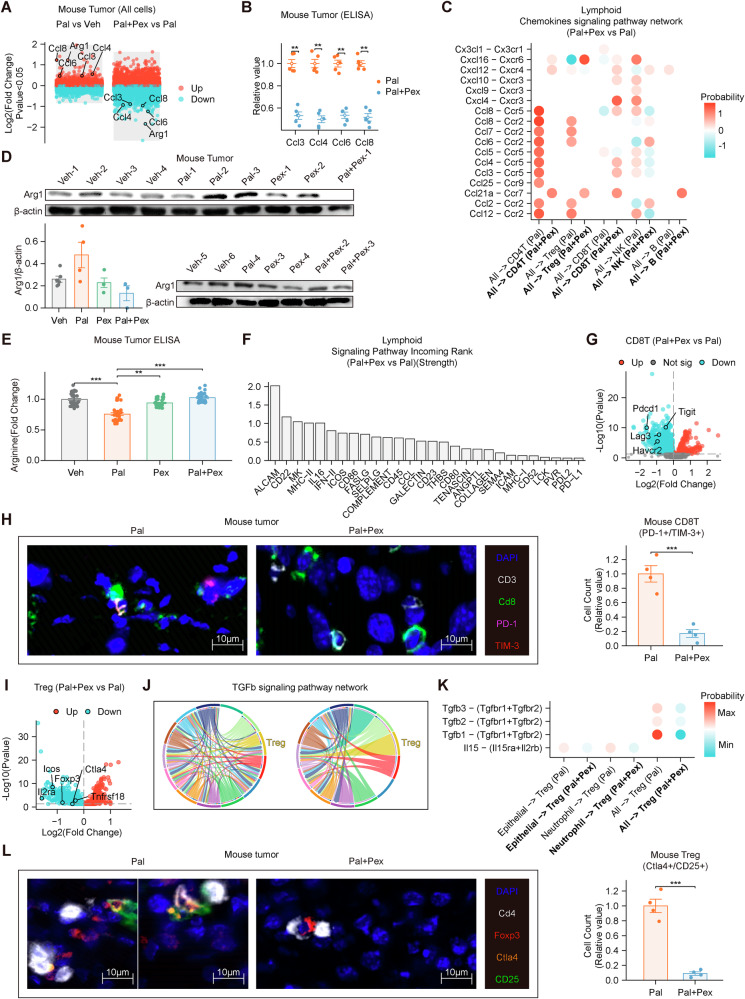
Pex reduces Arg1 expression and is associated with attenuation of CD8⁺ T cell exhaustion and Treg-mediated immunosuppression. **A** Pex inhibited the expression of Arg1, Ccl3, Ccl4, Ccl6, and Ccl8 within the tumor. **B** Pex reduces the secretion of Ccl3, Ccl4, Ccl6, and Ccl8 by mouse tumors (Pal: *n* = 10; Pal+Pex: *n* = 10). **C** Pex weakened the probability of a chemotactic effect of tumors on lymphocytes. **D** WB analyses of mouse tumors revealed that Pal tended to upregulate the expression of the Arg1 protein within tumors, whereas Pal+Pex downregulated Arg1 expression in mouse tumors. **E** Pal treatment reduced arginine levels, whereas Pex increased the arginine content within the tumor (Veh: *n* = 12; Pal: *n* = 9; Pex: *n* = 6; Pal+Pex: *n* = 3). **F** Pex increased the strength of signaling pathway communication related to lymphocyte activation. **G** RNA-seq analysis revealed that Pex reduced the expression of Pdcd1, Tigit, Lag3 and Havcr2 in CD8T (*n* = 18156). **H** mIHC staining revealed that Pex reduced Pdcd1 and Havcr2 expression on CD8T cells in mouse tumors (Pal: *n* = 3; Pal+Pex: *n* = 3). **I** Pex reduced the expression of the Il2ra, Foxp3, Icos, Ctla4, and Tnfrsf18 genes in Tregs. **J**, **K** Pex intervention weakened the communication of signaling pathways related to the activity of Tregs within the tumor microenvironment. **L** mIHC staining showed that Pex reduced Ctla4 and CD25 (Il2ra) expression on Tregs in mouse tumors (Pal: *n* = 3; Pal+Pex: *n* = 3). Veh: vehicle, Pal palbociclib, Pex pexidartinib, Pal+Pex palbociclib + pexidartinib. The data are shown as the means ± SEMs. **P < 0.01, ***P < 0.001, the Kruskal‒Wallis test and Wilcoxon rank sum test.

Transcriptome analysis of tumor tissues revealed that Pex intervention inhibited the tumor expression of *Arg1*, *Ccl3*, *Ccl4*, *Ccl6*, and *Ccl8*, which were upregulated by Pal, in tumors (Fig. 4A). elisas of mouse tumor tissues revealed that Pex reduced the expression of Ccl3, Ccl4, Ccl6, and Ccl8 in tumor tissues (Fig. 4B). The results of the cell communication analysis indicated that Pex weakened the probability of a chemotactic effect of tumors on lymphocytes (Fig. 4C). Arg1 in tumor tissues is expressed primarily by macrophages, and transcriptome analysis of macrophages revealed that Pal and Pex upregulated and downregulated the expression of *Arg1*, respectively, in macrophages (Supplementary Fig. S21). Western blot analysis of mouse tumor tissues revealed that Pal tended to upregulate the expression of the Arg1 protein within tumors, whereas Pex inhibited the expression of the Arg1 protein (Fig. 4D). This effect is twofold: the CSF1R inhibitor Pex suppresses ARG1 expression in macrophages and reduces the number of macrophages. Pal treatment reduced arginine levels, whereas Pex increased the arginine content within the tumor (Fig. 4E). This difference was relatively modest, likely due to the continual replenishment of arginine consumed within the tumor microenvironment [38]. Pex increased the degree of communication strength between signaling pathways related to the activation of lymphocytes within the TIME (Fig. 4F). The probability of communication related to lymphoid activation (MHC-I, MHC-II, CD80, CD86 and ICOS) decreased (Supplementary Figs. S22–26), and the change in the communication probability for FASLG was not significant (Supplementary Fig. S27). As a critical amino acid for lymphocyte function, arginine plays an essential role in sustaining T-cell activity. In our study, Pal treatment reduced arginine levels, potentially impairing lymphocyte viability and function. In contrast, Pex treatment restored arginine levels within the TIME. We screened a broad set of CD8⁺ T cell functional genes to comprehensively assess their activation, cytotoxicity, and exhaustion status. Among these, exhaustion-associated markers including Pdcd1, Tigit, Lag3, and Havcr2 (TIM-3) showed the most significant changes and were therefore highlighted (Fig. 4G–H), which was further confirmed by flow cytometry analysis of mouse tumor tissues (Supplementary Fig. S28A). These findings suggest that Pex may alleviate CD8⁺ T-cell exhaustion by reversing key molecular features associated with the exhausted phenotype.

Pex intervention reduced the expression of the *Il2ra*, *Foxp3*, *Icos*, *Ctla4*, and *Tnfrsf18* genes in Tregs (Fig. 4I). Cell communication analysis revealed that Pex intervention weakened the activation of signaling pathways related to the activity of Tregs within the TIME (Fig. 4J–K). mIHC revealed that Pex reduced Ctla4 and CD25 (Il2ra) expression on Tregs in mouse tumors (Fig. 4L). This finding was further validated by flow cytometry (Supplementary Fig. S28B). These findings suggest that the CSF1R inhibitor Pex suppresses Tregs. On the basis of the aforementioned hypothesis regarding Pal, Pex appears to remodel the antitumor TIME by restoring lymphocyte viability, potentially through targeting macrophages and reducing both their infiltration and Arg1 expression. Further studies are warranted to elucidate the mechanisms underlying this effect.

### Pal promotes fibroblast senescence and induces the secretion of IGF1 and FGF7

**Fig. 5:**
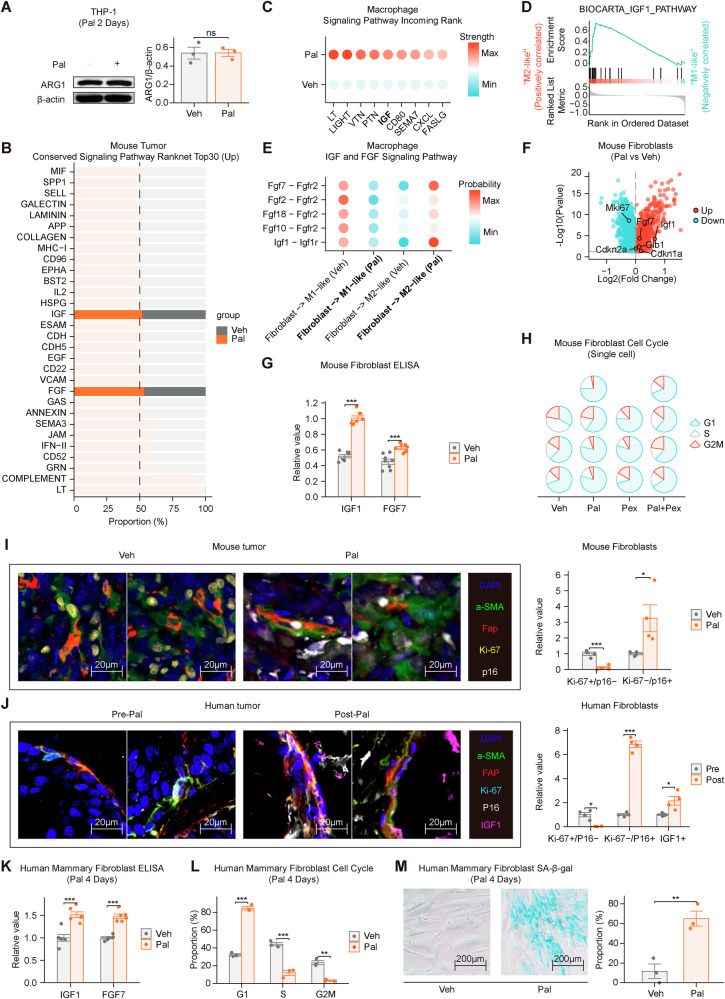
Pal promotes fibroblast senescence and induces the secretion of IGF1 and FGF7. **A** Pal alone did not directly modulate the polarization of PMA-induced THP-1-derived macrophages. **B** In mouse tumors, the IGF and FGF signaling pathways both presented a certain degree of increase among the top 30 conserved signaling pathways. **C** Ranking of cell communication received by macrophages revealed that Pal increased the strength of the ability of IGF to communicate with macrophages. **D** GSEA revealed that M2-like macrophages were enriched in the IGF1 pathway. **E** The probability of IGF and FGF communication between fibroblasts and M2-like macrophages was increased, whereas that between fibroblasts and M1-like macrophages was decreased. **F** Pal upregulated the expression of Igf1, Fgf7, Cdkn1a, Cdkn2a and Glb1, while downregulating Mki67 in mouse tumors (n = 4335). **G** ELISAanalysis revealed increased levels of IGF1 and FGF7 in fibroblasts from mouse tumor tissues**. H** Transcriptome analysis of the cell cycle revealed that Pal arrested fibroblasts in the G1 phase. **I** mIHC staining of mouse tumor tissues revealed that Pal suppressed Ki-67 expression in fibroblasts (a-SMA+Fap + ) and upregulated the senescence marker p16 (Veh: *n* = 3; Pal: *n* = 3). **J** mIHC analysis of HR⁺/HER2⁻ breast cancer tissues from patients treated with Pal also revealed reduced Ki-67 expression and increased P16 levels (Pre: *n* = 3; Post: *n* = 3). **K** ELISA results indicating that treatment of human primary mammary fibroblasts with Pal for four days upregulated the expression of IGF1 and FGF7. **L** Cell cycle analysis confirmed that Pal blocked the cell cycle of HMFs in the G1 phase. **M** β-Galactosidase assays indicated that Pal intervention caused senescence in human primary fibroblasts after four days (*n* = 3). Veh: vehicle, Pal: palbociclib. The data are shown as the means ± SEMs. *P < 0.05, **P < 0.01, ***P < 0.001, ns: not significant, t test, Welch’s one-way ANOVA and one-way ANOVA.

Pal alone did not directly modulate the polarization of PMA-induced THP-1-derived macrophages (Fig. 5A). Intercellular communication within the TIME was analyzed to explore the mechanism by which Pal promotes the polarization of tumor macrophages toward the M2-like phenotype and the overexpression of Arg1. In mouse tumors, the IGF and FGF signaling pathways both presented a certain degree of increase among the top 30 conserved signaling pathways (Fig. 5B). Cell communication targeting macrophages increased the extent to which the IGF signaling cascade was activated by macrophages (Fig. 5C). GSEA indicated that M2-like macrophages received more IGF1 signaling (Fig. 5D). Cell communication analysis indicated that intercellular communication involving IGF and FGF within the TIME is primarily mediated by fibroblasts (Supplementary Fig. S29A), and the probability of IGF and FGF communication between fibroblasts and M2-like macrophages increased, whereas the probability of communication between fibroblasts and M1-like macrophages decreased (Fig. 5E, Supplementary Fig. S29B). These findings suggest that Pal may influence macrophage polarization and Arg1 expression through the IGF and FGF pathways between fibroblasts and macrophages within the tumor, thereby affecting lymphocytes in the TIME.

Studies have shown that CDK4/6 inhibitors induce fibroblast senescence by disrupting the cell cycle of tissue fibroblasts [11], leading to the expression of SASP factors, which include IGF1 and FGF7. In our study, we screened fibroblast genes related to cell-cycle regulation, senescence, and key functional activities, and found that Pal promoted the expression of Igf1 and Fgf7 in fibroblasts (Fig. 5F). Additionally, Pal upregulated the expression of *Cdkn1a*, *Cdkn2a*, and *Glb1*, indicating that the fibroblast cycle was inhibited and that senescence was induced (Fig. 5F). ELISA revealed increased levels of IGF1 and FGF7 in fibroblasts from mouse tumor tissues (Fig. 5G). The analysis of the scRNA-seq data revealed that Pal blocked the cell cycle of fibroblasts in the G1 phase within mouse tumor tissues (Fig. 5H). mIHC of mouse tumor tissues revealed that Pal suppressed Ki-67 expression in fibroblasts (a-SMA + /Fap + ) and upregulated the senescence marker p16 (Fig. 5I). Consistently, mIHC analysis of fibroblasts isolated from HR + /HER2− breast cancer tissues of patients treated with Pal further showed decreased Ki-67 expression and elevated P16 levels, indicating the induction of fibroblast senescence. Notably, IGF1 expression in fibroblasts was also elevated following Pal treatment (Fig. 5J). This finding was validated by flow cytometry (Supplementary Fig. S30).

On the basis of the plasma concentration, the Pal concentration for human primary mammary fibroblasts (HMFs) was set at 0.4 μM [39]. After treating HMFs with Pal for four days in vitro, an increase in the protein expression of IGF1 and FGF7 in fibroblasts was detected via ELISA (Fig. 5K). Cell cycle analysis revealed that Pal blocked the cell cycle of HMFs in the G1 phase (Fig. 5L). Pal intervention induced senescence in HMFs after four days, as evidenced by β-Galactosidase assays (Fig. 5M, Supplementary Fig. S31).

### Pal promotes fibroblast secretion of IGF1 and FGF7 to activate macrophage Stat3 (Tyr705) phosphorylation leading to ARG1 expression and lymphocyte suppression

**Fig. 6:**
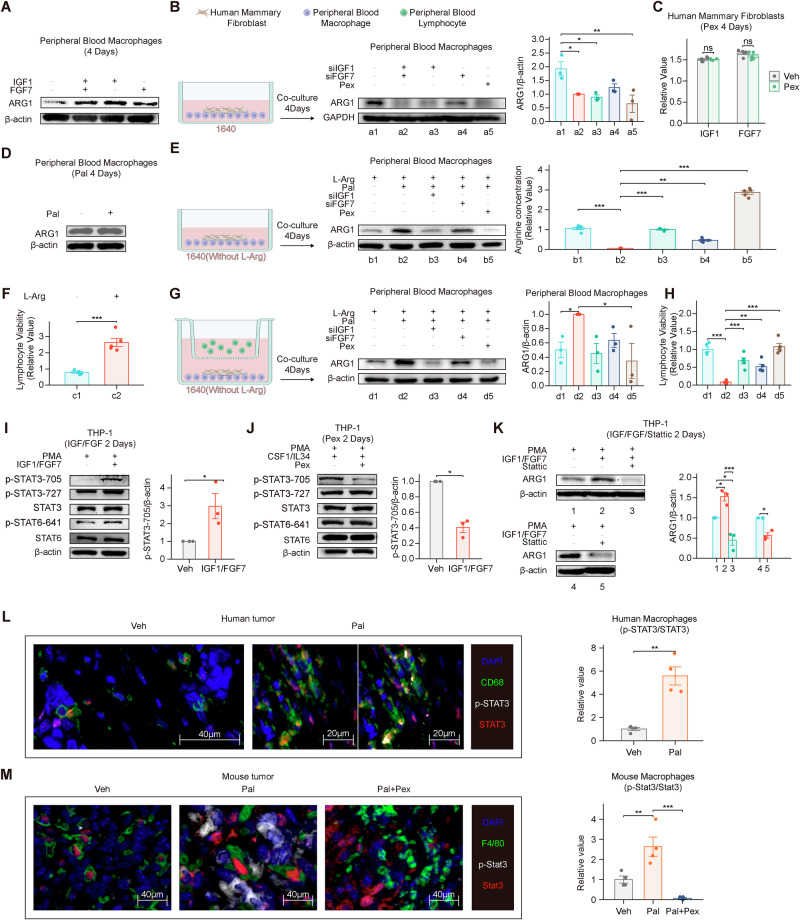
Pal promotes fibroblast secretion of IGF1 and FGF7 to activate macrophage Stat3 (Tyr705) phosphorylation leading to ARG1 expression and lymphocyte suppression. **A** IGF1 and FGF7 upregulated ARG1 expression in HPBMs. **B** Knockdown of IGF1 and FGF7 in HMFs, as well as treatment with Pex, inhibited ARG1 protein expression in HPBMs. **C** Pex did not affect the expression of IGF1 or FGF7 in HMFs. **D** Pal did not increase ARG1 expression in HPBMs. **E** ARG1 protein expression was upregulated in the Pal group of HPBMs, whereas siIGF1, siFGF7, and Pex inhibited ARG1 protein expression in HPBMs. ELISAof the arginine content in the coculture medium revealed the lowest arginine content in the b2 group and the highest content in the b5 group. **F** CCK-8 results indicating that arginine restored HPBL viability. **G** WB results showing that ARG1 protein expression was upregulated in the Pal group of HPBMs and that Pex inhibited ARG1 protein expression in HPBMs. **H** CCK-8 results revealed that the cell viability of the d2 group of HPBLs was the lowest and the survival rate was the highest in the d5 group. **I** IGF1 and FGF7 induced the phosphorylation of the STAT3 protein at tyrosine 705 in macrophages. **J** Pex inhibited STAT3 phosphorylation at tyrosine 705 in macrophages. **K** THP-1 cells were treated with 5 µM Stattic (a STAT3 phosphorylation inhibitor) for two days. Western blot (WB) analysis revealed that Stattic inhibited the expression of ARG1 protein, while IGF1 and FGF7 upregulated ARG1 expression in THP-1 cells. **L** mIHC analysis of HR + /HER2− breast cancer patient samples demonstrated that Pal induced STAT3 phosphorylation at tyrosine 705 in macrophages(Veh: *n* = 3; Pal: *n* = 3). **M** mIHC analysis of breast cancer-bearing mice revealed that Pal promoted STAT3 phosphorylation at tyrosine 705 in macrophages, whereas macrophage depletion resulted in the downregulation of STAT3 phosphorylation at this specific site (Veh: *n* = 3; Pal: *n* = 3; Pal+Pex: *n* = 3). Veh: vehicle, Pal: palbociclib, Pex: pexidartinib, Pal+Pex: palbociclib+pexidartinib, HMFs: human primary mammary fibroblasts, HPBMs: human peripheral blood primary macrophages, HPBLs: human peripheral blood primary lymphocytes. The data are shown as the means ± SEMs. *P < 0.05, **P < 0.01, ***P < 0.001, t test, Welch’s one-way ANOVA and one-way ANOVA.

Next, we investigated whether IGF1 and FGF7 expressed by fibroblasts affect the expression of ARG1 in macrophages. We cocultured HMFs, human peripheral blood primary macrophages (HPBMs), and human peripheral blood primary lymphocytes (HPBLs) in vitro. Cells were cultured in serum-free RPMI 1640 medium for four days. WB revealed that IGF1 and FGF7 upregulated ARG1 expression in HPBMs (Fig. 6A). Knockdown of IGF1 and FGF7 at the RNA and protein levels reduced the expression of IGF1 and FGF7 in HMFs (Supplementary Fig. S32A). Knockdown of IGF1 and FGF7 in HMFs, as well as treatment with Pex, inhibited ARG1 protein expression in HPBMs (Fig. 6B). Pex did not affect the expression of IGF1 or FGF7 in HMFs (Fig. 6C). Pal did not increase ARG1 expression in HPBMs (Fig. 6D). After the addition of 0.4 μM Pal to the coculture in RPMI 1640 medium without L-arginine and serum and the addition of 3 mM L-arginine followed by culture for four days, the WB results revealed that ARG1 protein expression was upregulated in the HPBMs from the Pal group, whereas siIGF1, siFGF7, and Pex inhibited the expression of ARG1 protein in the HPBM group. Elizas of the arginine content in the coculture medium revealed the lowest arginine content in the b2 group and the highest content in the b5 group (Fig. 6E). After HPBLs were cocultured in RPMI 1640 medium lacking L-arginine and serum, 3 mM L-arginine was added and the cells were cultured for an additional four days. CCK-8 assays revealed that arginine supplementation restored the viability of HPBLs (Fig. 6F), whereas PCR and ELISA results indicated the upregulation of MKI67 expression (Supplementary Figures S32B). After coculturing HMFs, HPBMs, and HPBLs for four days in RPMI 1640 medium without L-arginine or serum and adding 3 mM L-arginine, WB analysis revealed that ARG1 protein expression was upregulated in the HPBMs of the Pal group and the interference with Pex inhibited the expression of the ARG1 protein in HPBMs (Fig. 6G). The cell viability of HPBLs was lowest in the d2 group and highest in the d5 group (Fig. 6H). We validated the cellular communication and metabolic mechanisms identified in scRNA-seq analyses among fibroblasts, macrophages, and lymphocytes through the coculture of human primary cells. Using in vitro primary cell coculture, we verified that Pal promoted fibroblast secretion of IGF1 and FGF7, which in turn stimulated macrophage ARG1 expression and affected lymphocyte viability. We then investigated the underlying mechanisms.

Studies have shown that the activation of signaling pathways, such as the STAT3 and STAT6 pathways in macrophages, is closely related to the expression of ARG1 [40, 41]. To further investigate whether IGF1 and FGF7 affect macrophage ARG1 expression through these pathways, we selected THP-1 cells for in vitro mechanistic studies. THP-1 cells were treated with 100 ng/ml PMA for one day to induce their differentiation into macrophages and promote their adhesion. The macrophages were then treated with 100 ng/ml LPS and 20 ng/ml IFN-γ for two days to induce M1 macrophage polarization. Macrophages were treated with 20 ng/ml IL-4 and 20 ng/ml IL13 for two days to induce M2 macrophage polarization [42, 43]. After induction, the macrophages were treated with the corresponding recombinant proteins or drugs for two days (Supplementary Fig. S32C). Pex (5 μM) was used to inhibit the proliferation of THP-1 cells exposed to PMA (Supplementary Fig. S32D). The WB results obtained after THP-1 cell induction are shown in Supplementary Fig. S32E. Subsequently, THP-1 cells were treated with 50 ng/ml IGF1 and 50 ng/ml FGF7 for two days [44], and WB analysis revealed that IGF1 and FGF7 upregulated ARG1 protein expression in unpolarized macrophages and M2 macrophages (Supplementary Fig. S32F). IGF1 and FGF7 induced the phosphorylation of the STAT3 protein at site 705 in both unpolarized macrophages (Fig. 6I) and M2-polarized macrophages, whereas the STAT3 protein in M1 macrophages remained unphosphorylated (Supplementary Fig. S32G), possibly because negative regulatory factors are activated in M1 macrophages by IL4/IL13 induction, which inhibits the STAT3 pathway. Furthermore, LPS/IFN-γ-induced M1 macrophages typically lean toward inflammatory responses, whereas the STAT3 pathway in macrophages is associated primarily with anti-inflammatory or repair signaling, as observed in M2 macrophages. IGF1 and FGF7 slightly induced the phosphorylation of the AKT, ERK, and JNK proteins in macrophages (Supplementary Fig. S32H), and GSEA revealed that the ERK and MAPK signaling pathways were enriched in M2-like macrophages (Supplementary Fig. S32I). Subsequently, THP-1 cells were treated with 50 ng/ml CSF1, 50 ng/ml IL-34, and 5 μM Pex for two days in vitro [45–47], and CSF1 and IL-34 are ligands for the CSF1R receptor. WB results revealed that Pex inhibited the expression of the ARG1 protein in unpolarized macrophages and M2 macrophages (Supplementary Fig. S32J, K) and inhibited the phosphorylation of the STAT3 protein at tyrosine 705 in macrophages (Fig. 6J). THP-1 cells were treated with 5 µM Stattic (an inhibitor of STAT3 phosphorylation at tyrosine 705) (Supplementary Fig. S32D) for two days, and the WB results revealed that Stattic inhibited the phosphorylation of the STAT3 protein at tyrosine 705 and that the expression of the ARG1 protein was upregulated by IGF1 and FGF7 in THP-1 cells (Fig. 6K and Supplementary Fig. S32L). This may be due to the primary expression of ARG1 in M2 macrophages [48]. mIHC analysis revealed increased phosphorylation of STAT3 at tyrosine 705 in macrophages within tumor tissues from both post-Pal HR + /HER2− breast cancer patients and mouse models (Fig. 6L, M). This finding was validated by flow cytometry (Supplementary Fig. S33). Pex slightly inhibited the phosphorylation of the ERK protein in macrophages (Supplementary Fig. S32M). scRNA-seq and WB results indicated that Pal upregulated the expression of IGF1R and FGFR2 in unpolarized macrophages and M2-like macrophages (Supplementary Fig. S32N, O). On the basis of the above experiments, we found that IGF1, FGF7, and Pex affect ARG1 expression in macrophages by influencing STAT3 phosphorylation.

### The combination of Pal and Pex inhibits the growth of HR + /HER2− breast cancer organoids

**Fig. 7:**
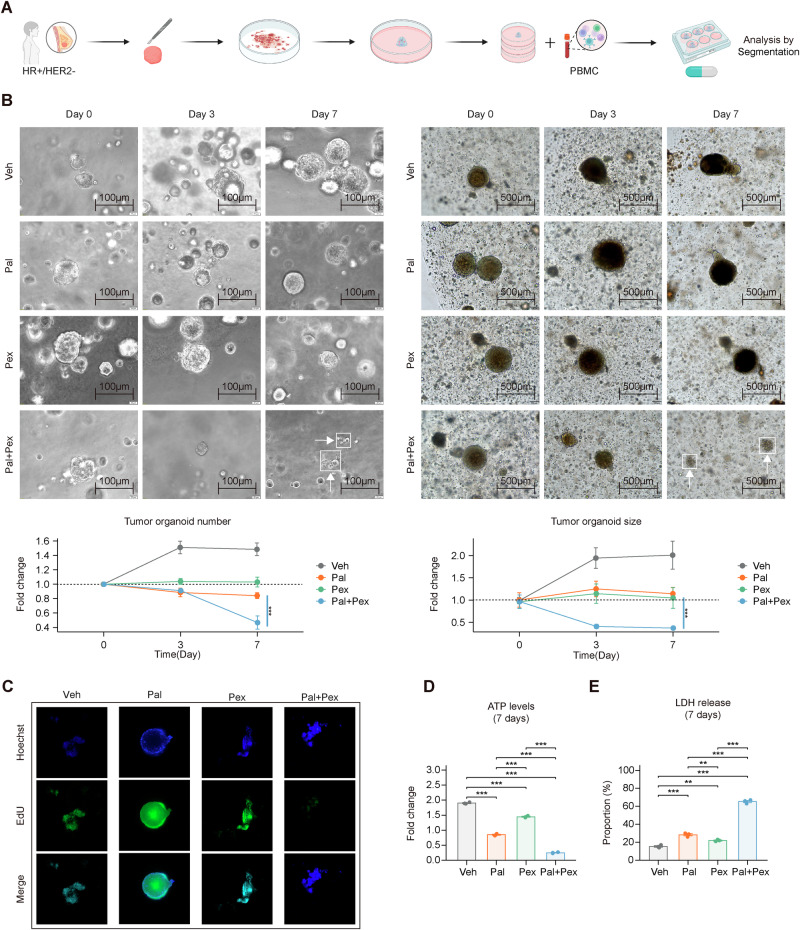
The combination of Pal and Pex inhibits the growth of HR + /HER2− breast cancer organoids. **A** Breast cancer organoids were established from primary tumor cells isolated from untreated HR + /HER2− breast cancer patients, and subsequently cocultured with peripheral blood mononuclear cells. The cultures were treated with Pal or Pex (*n* = 3). **B** Pal+Pex reduced the volume and number of organoids. **C**–**E** EdU incorporation experiments in organoids demonstrated that Pal+Pex inhibited the proliferation of breast cancer organoids. ATP and LDH assays further confirmed these findings, showing that Pal+Pex inhibited the growth of breast cancer organoids, as validated by confocal microscopy. Veh: vehicle, Pal: palbociclib, Pex: pexidartinib, Pal+Pex: palbociclib + pexidartinib.

Breast cancer organoids were established from primary tumor cells isolated from two untreated HR + /HER2− breast cancer patients, and subsequently cocultured with peripheral blood mononuclear cells (PBMCs). The cells were divided into groups according to the treatments received—Veh, Pal, Pex, and Pal+Pex, and the duration of treatment was seven days (Fig. 7A).

The breast cancer organoids were treated with Pal or Pex for seven days, and both agents inhibited tumor cell growth as early as the third day of treatment. After 7 days, Pal+Pex significantly inhibited tumor organoid growth. The organoid size and number were measured and analyzed using segmentation across different time points (Supplementary Figure S34). Compared with that on Day 0, the volume of the organoids in the Veh group increased by Day 7, while no significant change in the volume of the organoids in the Pal and Pex groups was observed by Day 7, and the volume of the organoids in the Pal+Pex group decreased. Compared with those on Day 1, the number of organoids in the Veh group increased by Day 7, while the number of organoids in the Pal and Pex groups decreased, and the volume of the organoids in the Pal+Pex group decreased (Fig. 7B). After the organoids were dispersed into a cell suspension, EdU staining was performed. In addition, ATP and LDH assays were conducted, and the results consistently demonstrated that Pal+Pex inhibited the growth of the breast cancer organoids, as confirmed by confocal microscopy (Fig. 7C–E).

## Discussion

Pharmacotherapeutic approaches for HR + /HER2− breast cancer patients require enhancement. Currently, there is a lack of systematic research on the TIME of HR + /HER2− breast cancer. As a first-line therapeutic option, CDK4/6 inhibitors may remodel the TIME, thereby offering a novel approach to optimize immunotherapeutic strategies. Therefore, elucidating the regulatory mechanisms by which CDK4/6 inhibitors modulate the TIME in HR + /HER2− breast cancer and exploring corresponding immunotherapeutic approaches holds significant clinical relevance.

For these reasons, we initially explored the potential of a CDK4/6 inhibitor to modulate the TIME of HR + /HER2− breast cancer. We utilized mouse models and conducted scRNA-seq and mIHC staining to investigate the effects of Pal on the immune microenvironment of breast cancer. Additionally, we performed mIHC staining on FFPE samples from patients treated with Pal. We found that the CDK4/6 inhibitor Pal promoted macrophage polarization toward the M2-like phenotype and promoted the expression of ARG1. Our findings are consistent with the recent findings of Allison L. Creason, who described the immunohistochemical pathological characteristics of HR + /HER2− breast cancer patients and suggested that CDK4/6 inhibitors lead to T-cell dysfunction and increased infiltration of CD163+ macrophages [49]. We also found that Pal enhanced the production of several chemokines in tumors, which may facilitate the chemotaxis of lymphocytes into tumors, aligning with the findings of Uzhachenko et al. [50]. However, there was no significant change in lymphocyte counts in the TIME, which contradicts the current findings that CDK4/6 inhibitors upregulate peripheral blood lymphocytes and inhibit peripheral blood Tregs [16, 51]. To date, human studies have focused primarily on immune cells in the blood [15, 16, 51], and the results from animal studies on immune cells within tumors are inconsistent [50, 52–54]. Based on our cellular communication and transcriptomic analyses, we hypothesized that Pal influences macrophage polarization and Arg1 expression by inducing fibroblast senescence and the subsequent secretion of IGF1 and FGF7, thereby affecting lymphocyte viability within the tumor. Keyomarsi et al. recently reported that CDK4/6 inhibitors induce CD8⁺ T cell exhaustion in the tumor microenvironment of breast cancer. Our work validates this observation and provides mechanistic insight [55]. Building on current research, we propose a new mechanism by which Pal mediates tumor immune suppression in the TIME. These results suggest that Pal may inhibit lymphocyte viability through a novel mechanism within the tumor that is primarily mediated by macrophages, exerting immunosuppressive effects.

Notably, combination therapy with high-dose paclitaxel and Pex has been explored in TNBC, demonstrating that remodeling the TIME with high-dose paclitaxel can enhance the therapeutic efficacy of Pex [56]. Studies have shown that the proportion of macrophages in HR + /HER2− breast cancer is greater than that in TNBC, and the proportion of lymphocytes in HR + /HER2− breast cancer is less than that in TNBC [6, 7], suggesting that the use of immunotherapy drugs targeting macrophages to treat HR + /HER2− breast cancer might be a better choice. Combining the results above, we administered two types of macrophage-targeted drugs, the CSF1R inhibitor Pex [57] and the CCR2 inhibitor INCB3344 [30], to mouse models of breast cancer via the ER + /HER2- 67NR cell line and the TNBC 4T1 cell line. In the 67NR model, we found that Pal combined with Pex had a better tumor-killing effect in mouse experiments. A comparison of tumor growth rates in mice showed that Pex inhibited Pal's immunosuppressive effects on mouse tumors. Previous studies have shown that palbociclib primarily exerts its antitumor activity by blocking tumor cell cycle progression through CDK4/6 inhibition. However, in the 4T1 TNBC model, where CDK4/6 expression is relatively low and Rb loss is common (Supplementary Fig. S1D, E), the direct antiproliferative effect of palbociclib is limited and insufficient to offset the immunosuppressive changes it induces. Our study further uncovers a novel mechanism, demonstrating that palbociclib not only lacks potent cell-cycle inhibition in 4T1 cells but also remodels the TIME, promoting M2-like macrophage polarization and upregulating immunosuppressive molecules, such as ARG1. Pex effectively counteracts this palbociclib-induced immunosuppression, thereby improving the antitumor immune response in this model. Research by Patel and Basu indicated that TNBC, characterized by a lack of AR expression and BRCA1 mutation, often exhibits Rb loss and is therefore considered a poor candidate for CDK4/6 inhibition [58, 59]. These findings further underscore the potent immunosuppressive effects of Pal on the TIME, particularly in contexts where its direct antitumor efficacy is limited.

We further explored the mechanism underlying these phenomena in vitro. HMFs, HPBMs, HPBLs, and THP-1 cells were used. We confirmed that Pal induced senescence in HMFs; promoted the release of SASP components [18, 60], including IGF1 and FGF7; and affected macrophage ARG1 expression and immunosuppressive functions. Arg1 is a characteristic marker of M2-like macrophages in mice. Similarly, in humans, an increase in Arg1 expression in macrophages, particularly M2-like macrophages, is often associated with a tumor immunosuppressive environment [61–63]. The primary function of Arg1 is to metabolize arginine, a crucial amino acid necessary for lymphocyte proliferation and function [48, 64]. Griffiths et al. reported that the combination of radiotherapy (RT), CDK4/6 inhibitors, and ET promotes macrophage polarization toward the M2-like phenotype in mice [65]. Our study provides a preliminary explanation of the potential mechanism underlying this phenomenon. Research indicates that IGF and FGF can affect the activity of multiple intracellular signaling pathways, such as the STAT3 pathway [66, 67], which in turn can influence ARG1 expression [40, 41]. In our experiments, we found that IGF1 and FGF7 primarily regulate ARG1 expression by modulating STAT3 phosphorylation at tyrosine 705 in macrophages. Moreover, these pathways can prevent the cell cycle blockade effect of Pal [68, 69], explaining why the effect of Pal on cell cycle blockade is more evident in M1-like macrophages (Supplementary Figure S6C). Additionally, Pex primarily inhibited STAT3 signaling, thereby counteracting the immunosuppressive effects induced by Pal. Based on the aforementioned experiments, we validated the effects of Pal and Pex on fibroblasts, macrophages, lymphocytes, and tumor cells within the TIME, as well as the potential mechanisms underlying these effects.

Finally, we isolated primary cells from the tumor tissues of two HR + /HER2− breast cancer patients who had not received any treatment and cultured them as HR + /HER2− breast cancer organoids. By measuring the number and volume of organoids and their proliferative activity, we found that Pex increased the tumor-killing effect of Pal. We documented the effectiveness of combined treatment with Pal and Pex in a model closer to the TIME of HR + /HER2− breast cancer patients.

In addition, we observed that Tregs did not show a significant change in overall abundance following Pal treatment. However, Pex intervention markedly reduced the expression of key Treg activation and immunosuppressive markers, including Il2ra, Foxp3, Icos, Ctla4, and Tnfrsf18, thereby weakening Treg-associated activation signals. These findings suggest that while Pal alone does not substantially alter Treg numbers, the immunosuppressive TIME induced by Pal may support the maintenance of Treg functional activity, and CSF1R inhibition can counteract this effect. Recent studies further support the predictive value of Tregs in immunotherapy response. For instance, Wang et al. demonstrated that both the infiltration level and functional state of Tregs within the tumor are closely correlated with the efficacy of immune checkpoint inhibitors (ICIs), highlighting their potential as predictive biomarkers for immunotherapy responsiveness [70]. Our findings align with this concept: although Pal alone did not significantly reduce Treg numbers, Pex suppressed Treg functional activation and dampened Treg-mediated signaling within the TIME, suggesting that targeting macrophage-driven immunosuppressive pathways to indirectly modulate Tregs may enhance the sensitivity of HR + /HER2− breast cancer to immunotherapy.

Although we have made progress in the immunotherapy of HR + /HER2− breast cancer, our study still has limitations. The robustness of our statistical analysis is affected by the sample size of the clinical patient cohort, and future studies can further investigate the effects of treatment combining Pal with Pex on HR + /HER2− breast cancer by increasing the number of organoid models. The potential side effects of combined treatment with CDK4/6 inhibitors and CSF1R inhibitors remain underexplored, and further research is needed in animal models and clinical trials. Given the absence of patient-derived xenograft (PDX) models, further validation in animal models and clinical settings is warranted.

## Conclusion

In conclusion, our study revealed previously undiscovered immunosuppressive mechanisms of CDK4/6 inhibitors, which not only promote macrophage polarization toward M2-like macrophages but also upregulate ARG1 expression, driving arginine depletion in the TIME and inhibiting lymphocyte viability. A CSF1R inhibitor (pexidartinib) improved immune tolerance induced by a CDK4/6 inhibitor (palbociclib) in HR + /HER2− breast cancer by blocking this proposed mechanism. Given the poor efficacy of CDK4/6 inhibitors in NAT, their benefit in postoperative adjuvant therapy remains uncertain [71]. Combined treatment with a CDK4/6 inhibitor and a CSF1R inhibitor has good application prospects for the treatment of HR + /HER2− breast cancer, but whether this regimen can provide long-term benefits to patients remains to be studied.

## Supplementary information

Supplementary Figure

Supplementary Data (mIHC)

Supplementary Data (Western Blot)

## Data Availability

The genomic sequence data described in this study have been archived in the National Genomics Data Center’s Genome Sequence Archive (Accession Code: CRA013697), maintained by the Beijing Institute of Genomics at the Chinese Academy of Sciences. These datasets are publicly available through the repository portal at https://ngdc.cncb.ac.cn/gsa.
