# Supplementary material for: CSF1R inhibitors mitigate CDK4/6 inhibitor-induced immunosuppression to increase antitumor immunity in HR+/HER2− breast cancer
Source: Oncogene. 2026 Apr 15;45(21):1970–87. doi: 10.1038/s41388-026-03786-w (PMC13190250; doi:10.1038/s41388-026-03786-w)
Supplement: Supplementary file 1 — Supplementary Figure [file 41388_2026_3786_MOESM1_ESM.pdf]

Supplementary Materials for

CSF1R Inhibitors Mitigate CDK4/6 Inhibitor-Induced Immunosuppression to Increase Antitumor Immunity in HR+/HER2- Breast Cancer

Siwei Li *et al.*

*Corresponding author: Da Pang, Email: pangda@ems.hrbmu.edu.cn

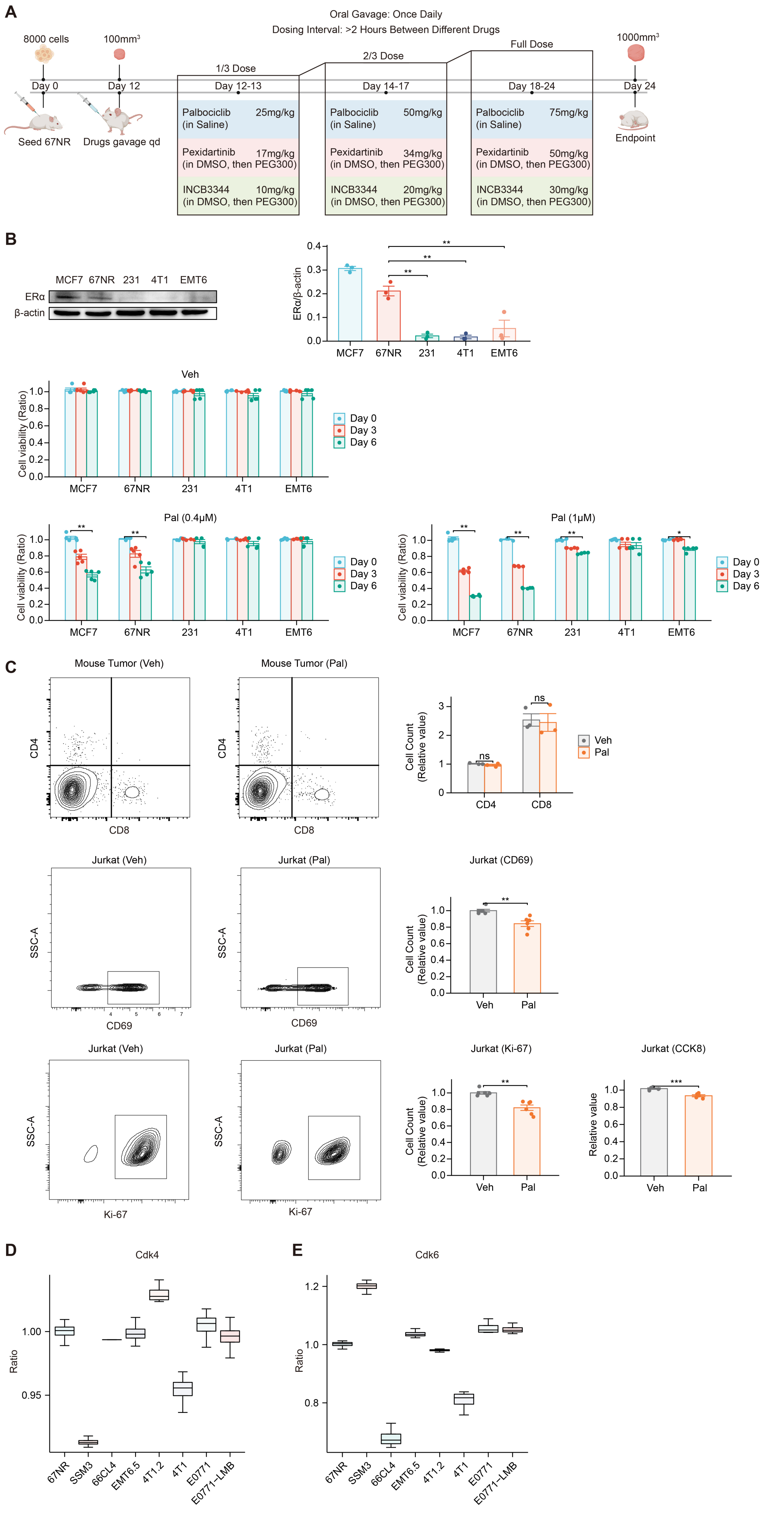
A
Oral Gavage: Once Daily
Dosing Interval: >2 Hours Between Different Drugs
8000 cells
100mm³
1000mm³
1/3 Dose
2/3 Dose
Full Dose
Day 0
Day 12
Day 12-13
Day 14-17
Day 18-24
Day 24
Seed 67NR
Drugs gavage qd
Endpoint
Palbociclib 25mg/kg (in Saline)
Pexidartinib 17mg/kg (in DMSO, then PEG300)
INCB3344 10mg/kg (in DMSO, then PEG300)
Palbociclib 50mg/kg (in Saline)
Pexidartinib 34mg/kg (in DMSO, then PEG300)
INCB3344 20mg/kg (in DMSO, then PEG300)
Palbociclib 75mg/kg (in Saline)
Pexidartinib 50mg/kg (in DMSO, then PEG300)
INCB3344 30mg/kg (in DMSO, then PEG300)
B
MCF7 67NR 231 4T1 EMT6
ERα
β-actin
ERα/β-actin
Veh
Pal (0.4μM)
Pal (1μM)
Cell viability (Ratio)
Day 0
Day 3
Day 6
C
Mouse Tumor (Veh)
Mouse Tumor (Pal)
CD4
CD8
Cell Count (Relative value)
ns
Veh
Pal
Jurkat (Veh)
Jurkat (Pal)
Jurkat (CD69)
SSC-A
CD69
Ki-67
Jurkat (Ki-67)
Jurkat (CCK8)
Relative value
D
Cdk4
Ratio
67NR
SSM3
66CL4
EMT6.5
4T1.2
4T1
E0771
E0771-LMB
E
Cdk6

Fig. S1. Expression levels of *Cdk4* and *Cdk6* in 67NR cells.

(**A**) Schematic Overview of Drug Administration in the Mouse Model.

(**B**) 67NR cells express ERα at a lower level than the ER-positive control MCF7, but markedly higher than the ER-negative cell lines MDA-MB-231, 4T1, and EMT6. CCK-8 (6 days) showed that 0.4 μM palbociclib inhibited MCF7 and 67NR, with no detectable effects on MDA-MB-231, 4T1, or EMT6. At 1 μM, inhibition was stronger in MCF7/67NR, modest in MDA-MB-231/EMT6, and negligible in 4T1.

(**C**) Flow cytometric analysis of murine tumor tissues revealed no significant changes in the number of $CD4^+$ and $CD8^+$ T cells within the tumor immune microenvironment (TIME) following treatment with Pal. Pal modestly reduced Jurkat cell activation, as evidenced by decreased CD69 and Ki-67 expression to approximately 80% of the stimulated control after 48 h. CCK-8 assays demonstrated that Pal reduced Jurkat cell viability in vitro.

(**D**, **E**) *Cdk4* and *Cdk6* expression in 67NR cells. Data were obtained from the GSE160101, GSE226910, GSE11259 and GSE42272 datasets.

A

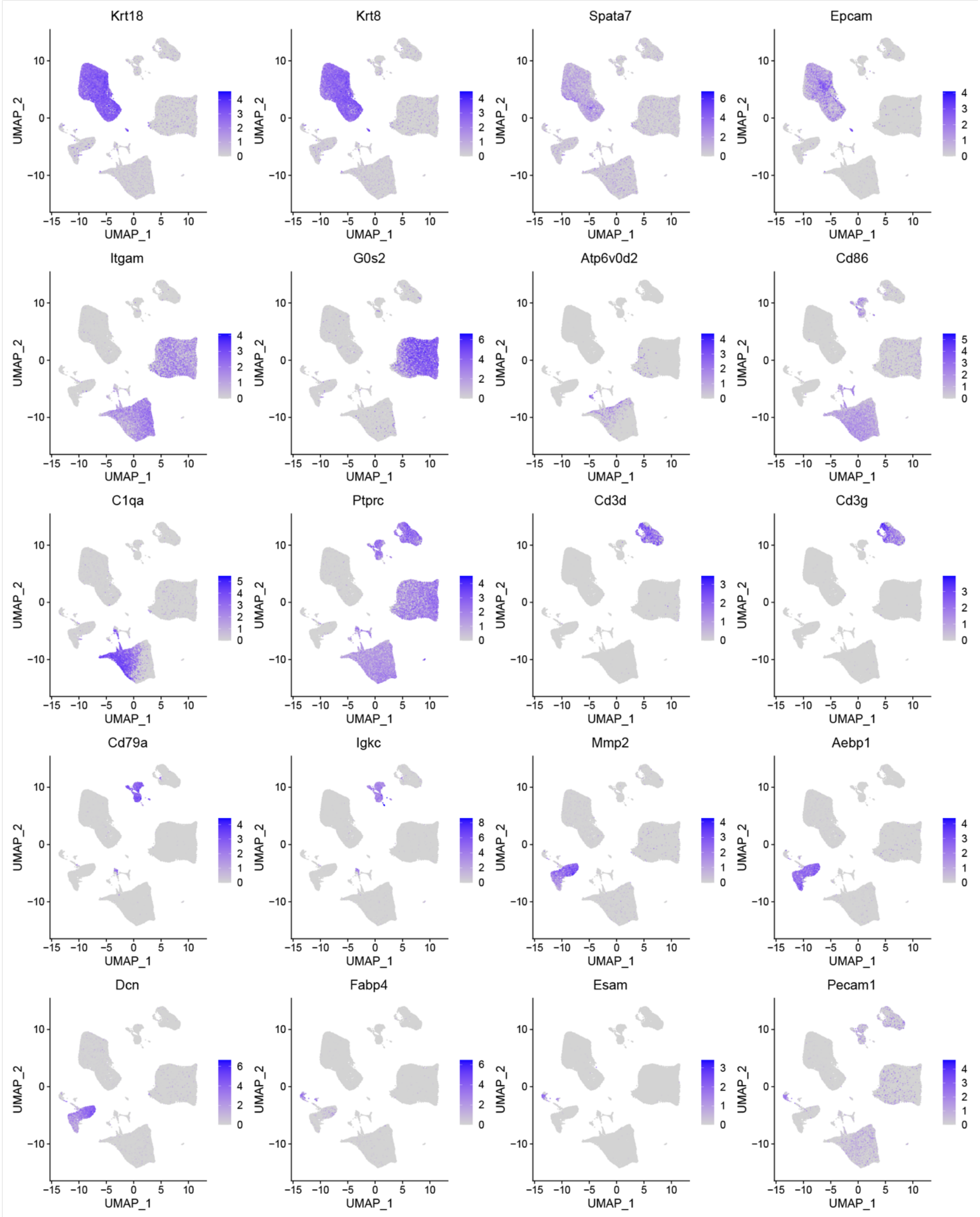

Fig. S2. UMAP of single-cell sequencing data for mouse tumors.

(**A**) Individual UMAP plot for each gene.

A

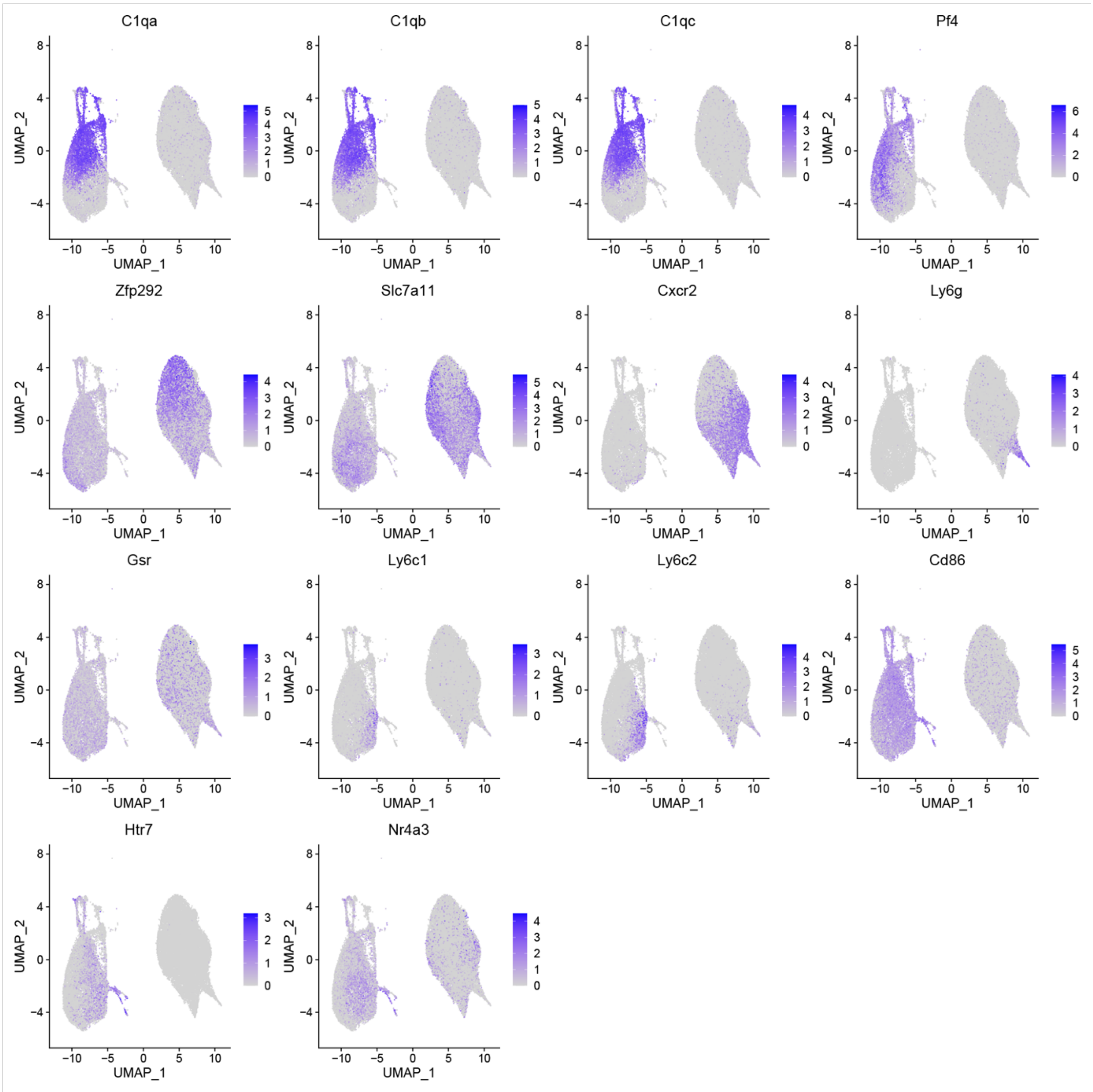

Fig. S3. UMAP of single-cell sequencing data of myeloid cells.

(**A**) Individual UMAP plot for each gene.

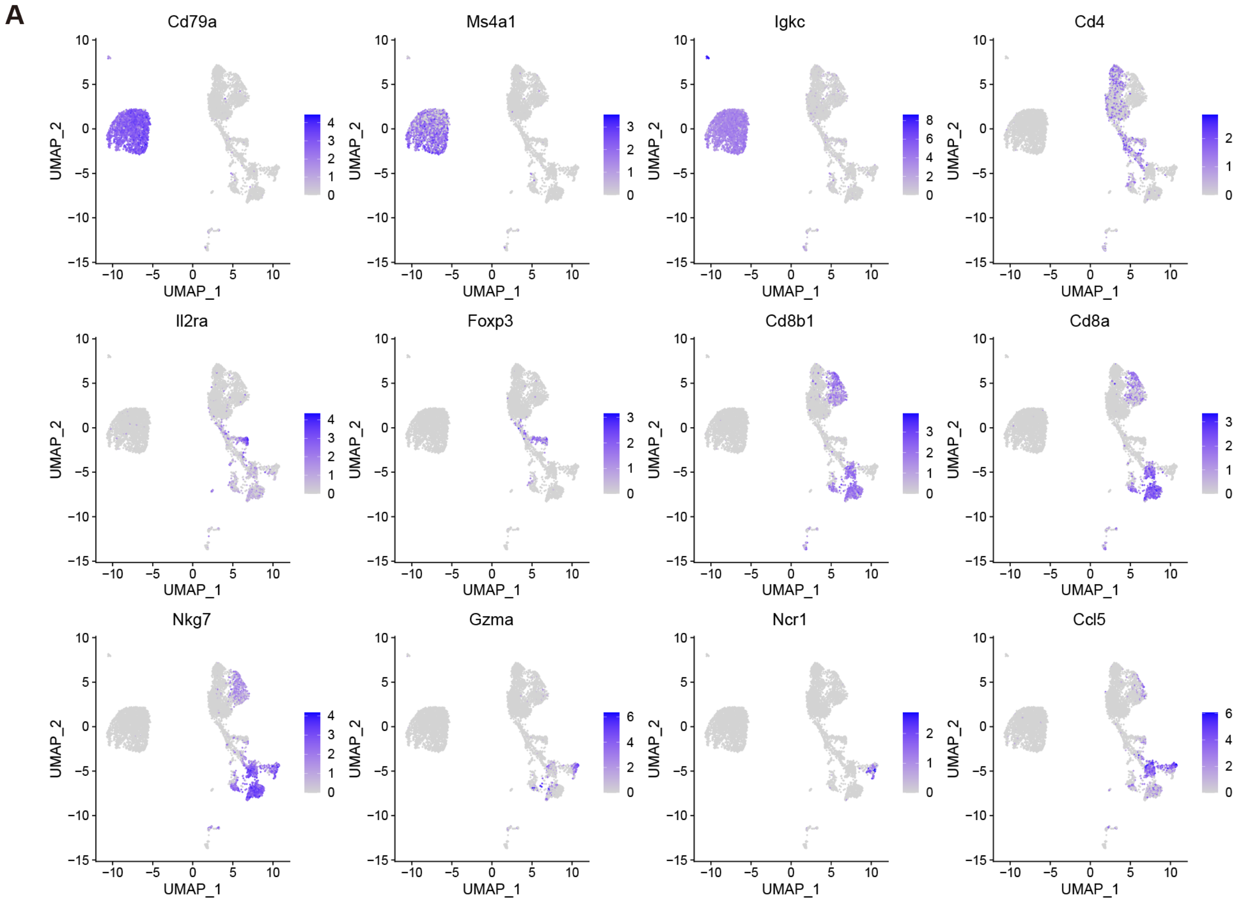

Fig. S4. UMAP of single-cell sequencing data of lymphoid cells.

(**A**) Individual UMAP plot for each gene.

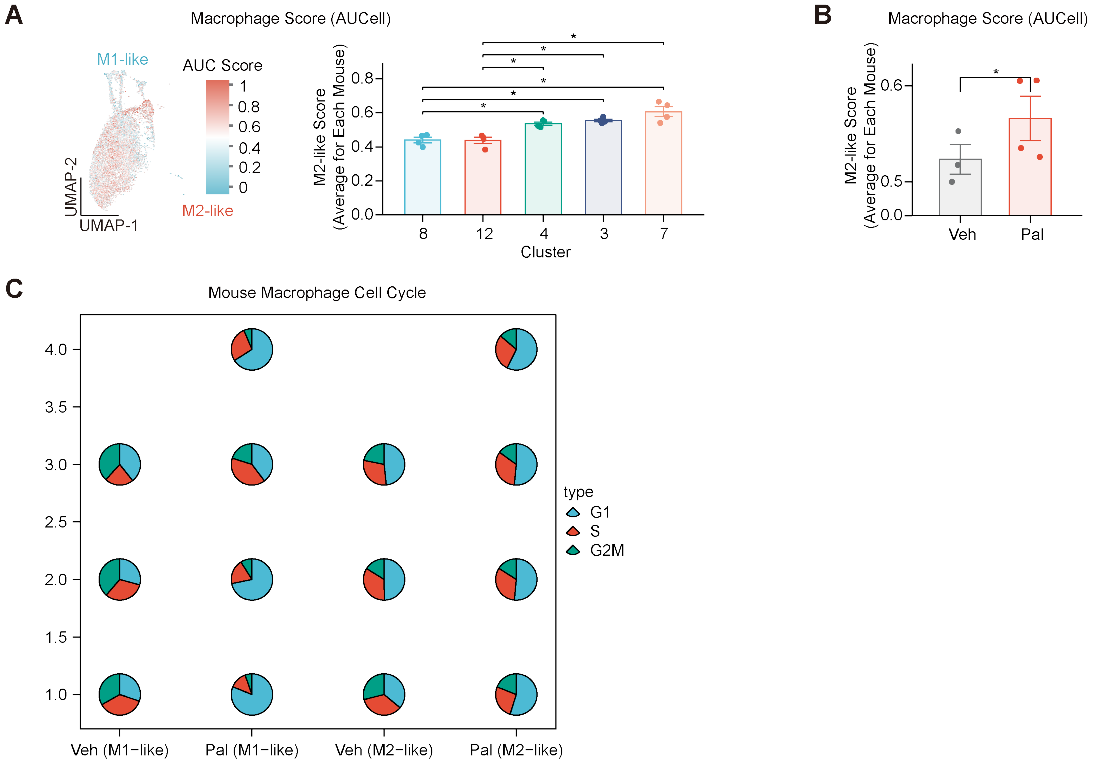

Fig. S5. AUCell score and cell cycle analysis of mouse macrophages based on single-cell data.

(**A** and **B**) AUCell score analysis of mouse macrophages based on single-cell data.

(**C**) Cell cycle analysis of mouse macrophages based on single-cell data.

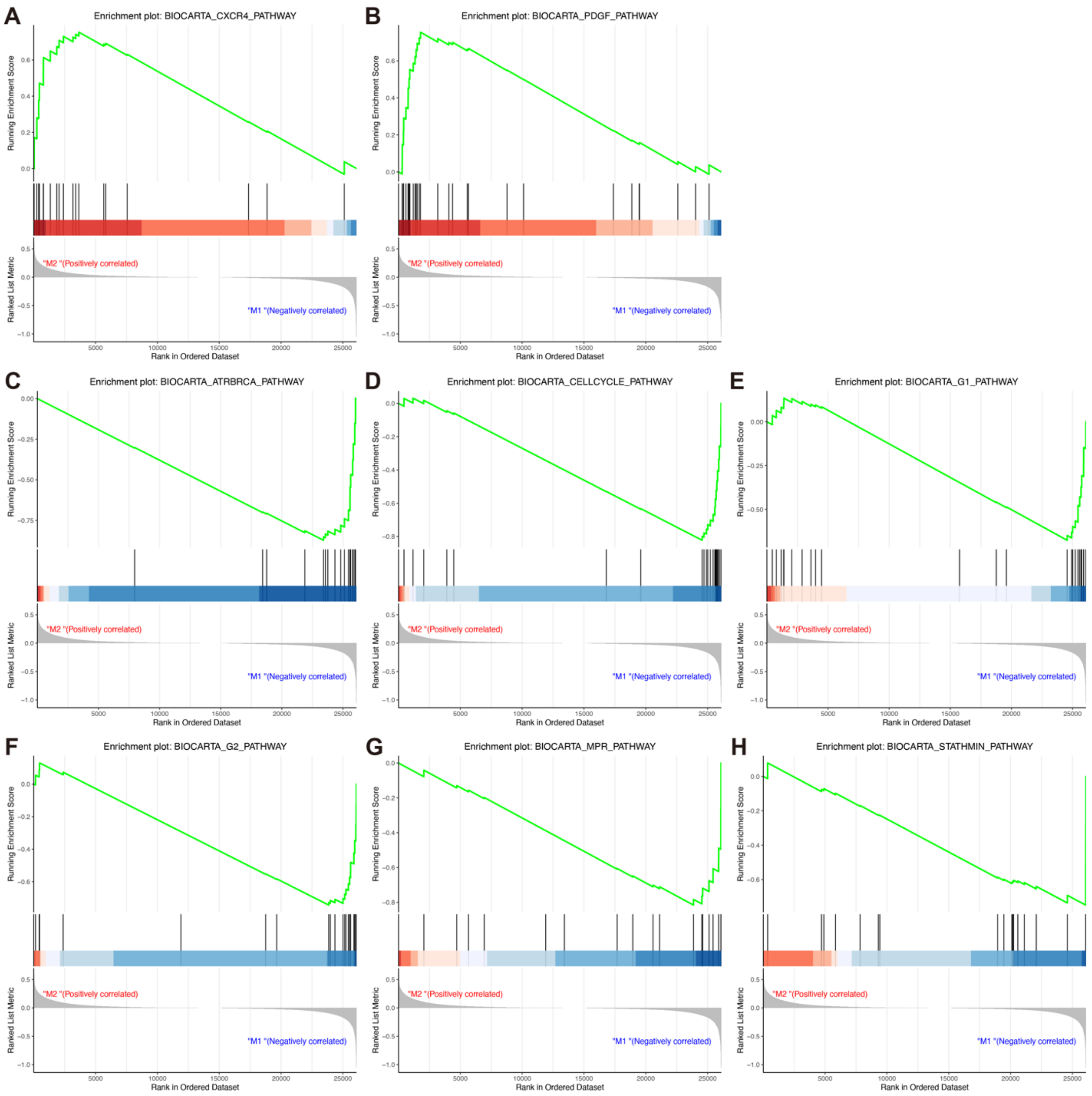

Fig. S6. GSEA enrichment analysis of macrophages.

(**A**) Individual GSEA enrichment analysis for each.

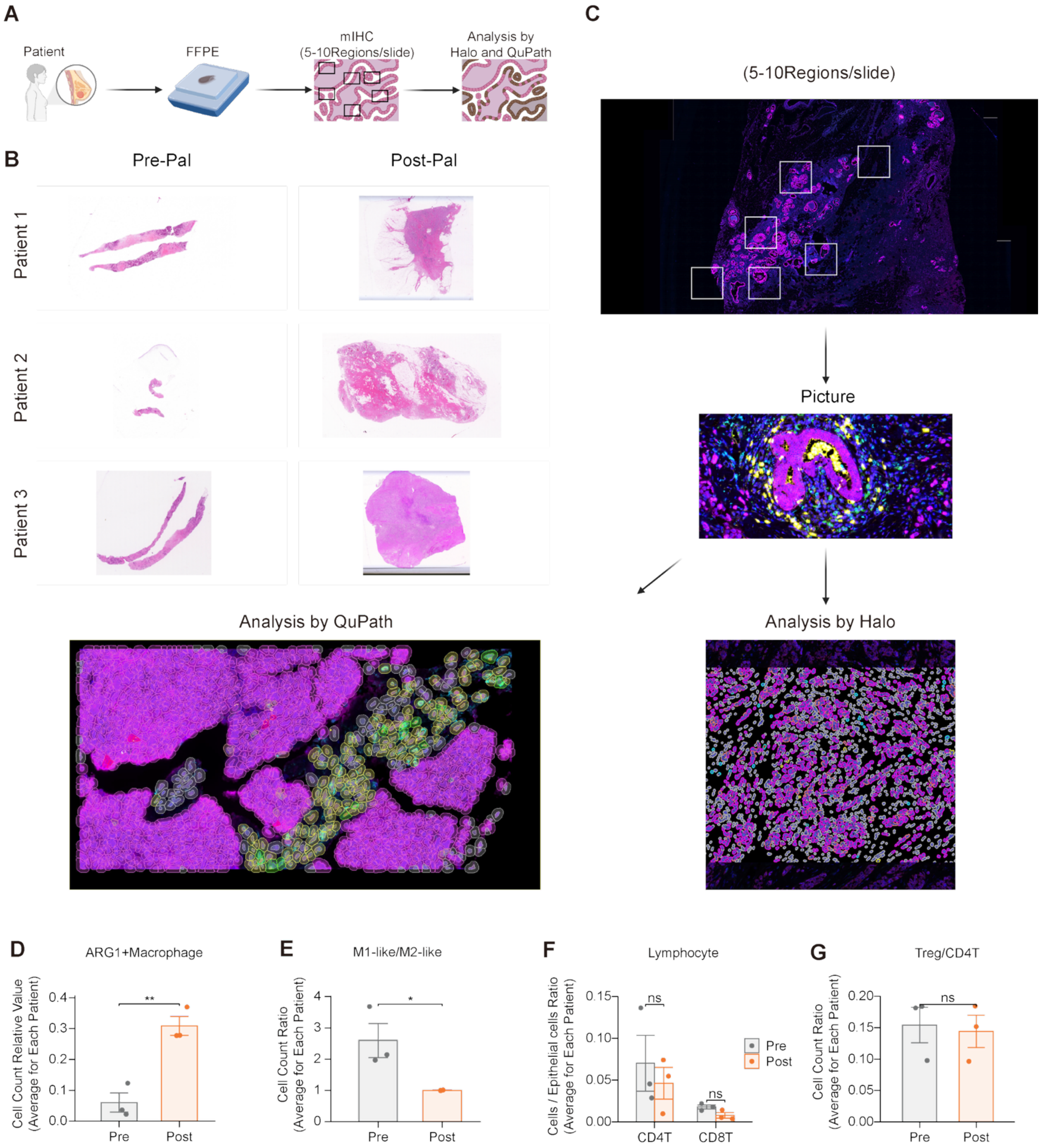

Fig. S7. Illustration of the steps of mIHC analysis.

(**A**) Hematoxylin and eosin (HE) staining of FFPE samples from three patients.

(**B** and **C**) Illustration of the steps of mIHC analysis.

(**D** and **E**) Pal decreased the number of M1-like macrophages (PANCK-CD68+CD86+), increased the number of M2-like macrophages (PANCK-CD68+CD86-CD206+), and increased the number of ARG1+ macrophages.

(**F** and **G**) No significant differences in the numbers of CD4-positive T cells (CD4T) (PANCK-CD4+CD8-FOXP3-), CD8-positive T cells (CD8T) (PANCK-CD4-CD8+FOXP3-), or regulatory T cells (Treg) (PANCK-CD4+CD8-FOXP3+) were observed.

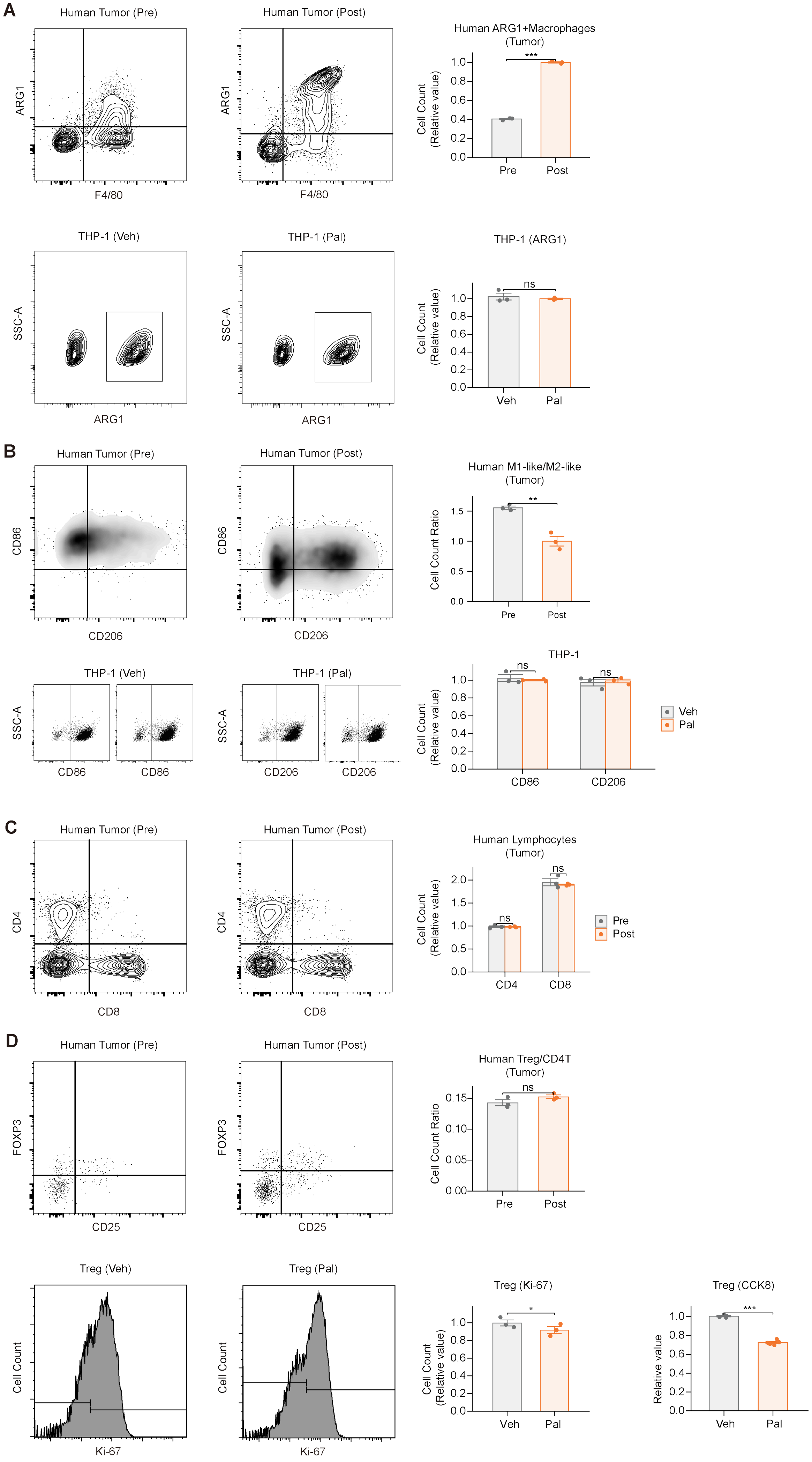
A
Human Tumor (Pre)
Human Tumor (Post)
ARG1
F4/80
Human ARG1+Macrophages (Tumor)
Cell Count (Relative value)
***
Pre
Post
THP-1 (Veh)
THP-1 (Pal)
SSC-A
ARG1
THP-1 (ARG1)
ns
Veh
Pal
B
Human Tumor (Pre)
Human Tumor (Post)
CD86
CD206
Human M1-like/M2-like (Tumor)
Cell Count Ratio
**
Pre
Post
THP-1 (Veh)
THP-1 (Pal)
SSC-A
CD86
CD206
THP-1
ns
Veh
Pal
C
Human Tumor (Pre)
Human Tumor (Post)
CD4
CD8
Human Lymphocytes (Tumor)
ns
Pre
Post
D
Human Tumor (Pre)
Human Tumor (Post)
FOXP3
CD25
Human Treg/CD4T (Tumor)
Cell Count Ratio
ns
Treg (Veh)
Treg (Pal)
Cell Count
Ki-67
Treg (Ki-67)
*
Treg (CCK8)
Relative value
***

Fig. S8. Flow cytometry analysis of human tumor tissues, THP-1 cells, and Tregs.

(**A** and **B**) Pal decreased the number of M1-like macrophages while increasing M2-like macrophages and $ARG1^+$ macrophages in the human TME. In contrast, in vitro treatment of THP-1–derived macrophages with Pal did not significantly alter the expression of ARG1, CD86, or CD206.

(**C** and **D**) No significant differences in the numbers of $CD4^+$ T cells (CD4T), $CD8^+$ T cells (CD8T), or regulatory T cells (Tregs) were observed in the human TME. Consistently, in vitro treatment of primary human Tregs with Pal reduced Ki-67 expression and decreased cell viability, as assessed by CCK-8 assays.

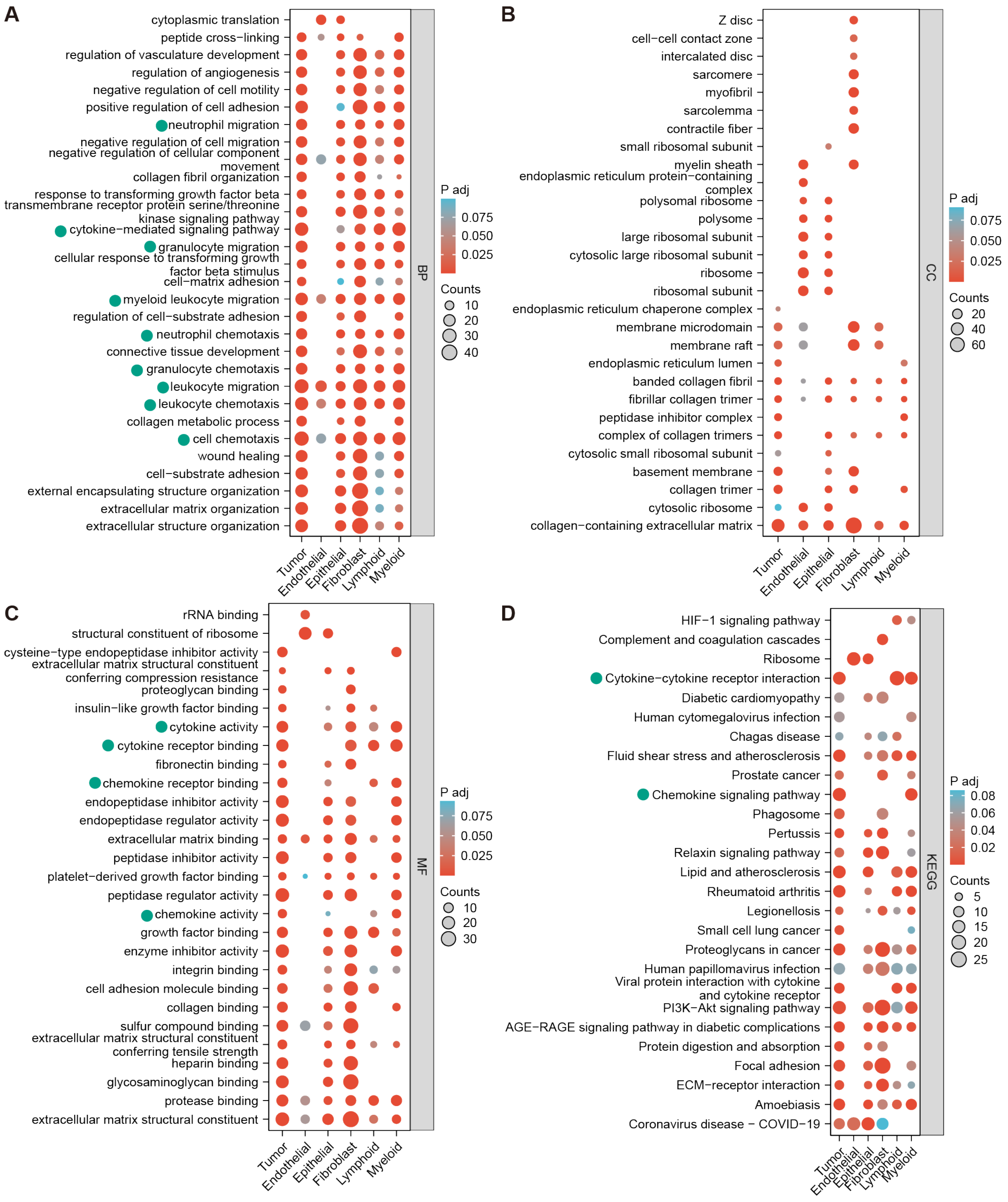

Fig. S9. GO/KEGG enrichment analysis of mouse tumors.

(**A**) BP enrichment analysis.

(**B**) CC enrichment analysis.

(**C**) MF enrichment analysis.

(**D**) KEGG enrichment analysis.

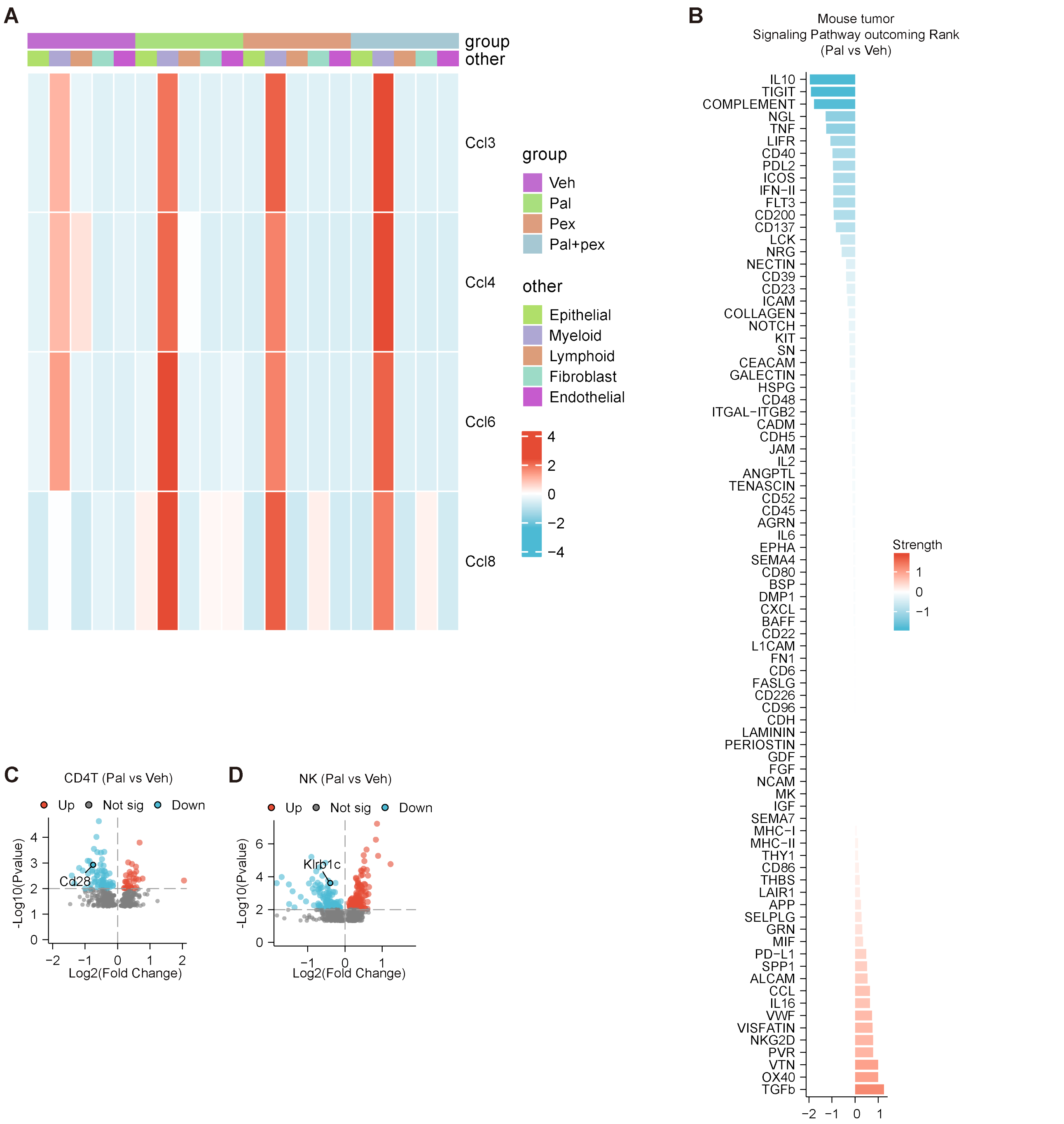

Fig. S10. Pal inhibited the activity of lymphocytes within the tumor.

(**A**) *Ccl3*, *Ccl4*, *Ccl6*, and *Ccl8* are primarily expressed by myeloid cells within the tumor, and Pex does not inhibit the expression of these chemokines.

(**B**) Analysis of the strength of cell communication pathways related to lymphoid cells in mouse tumor tissue.

(**C**) The expression of *Cd28* in CD4+ T cells was downregulated.

(**D**) The expression of *Klrb1c* in NK cells was downregulated.

A

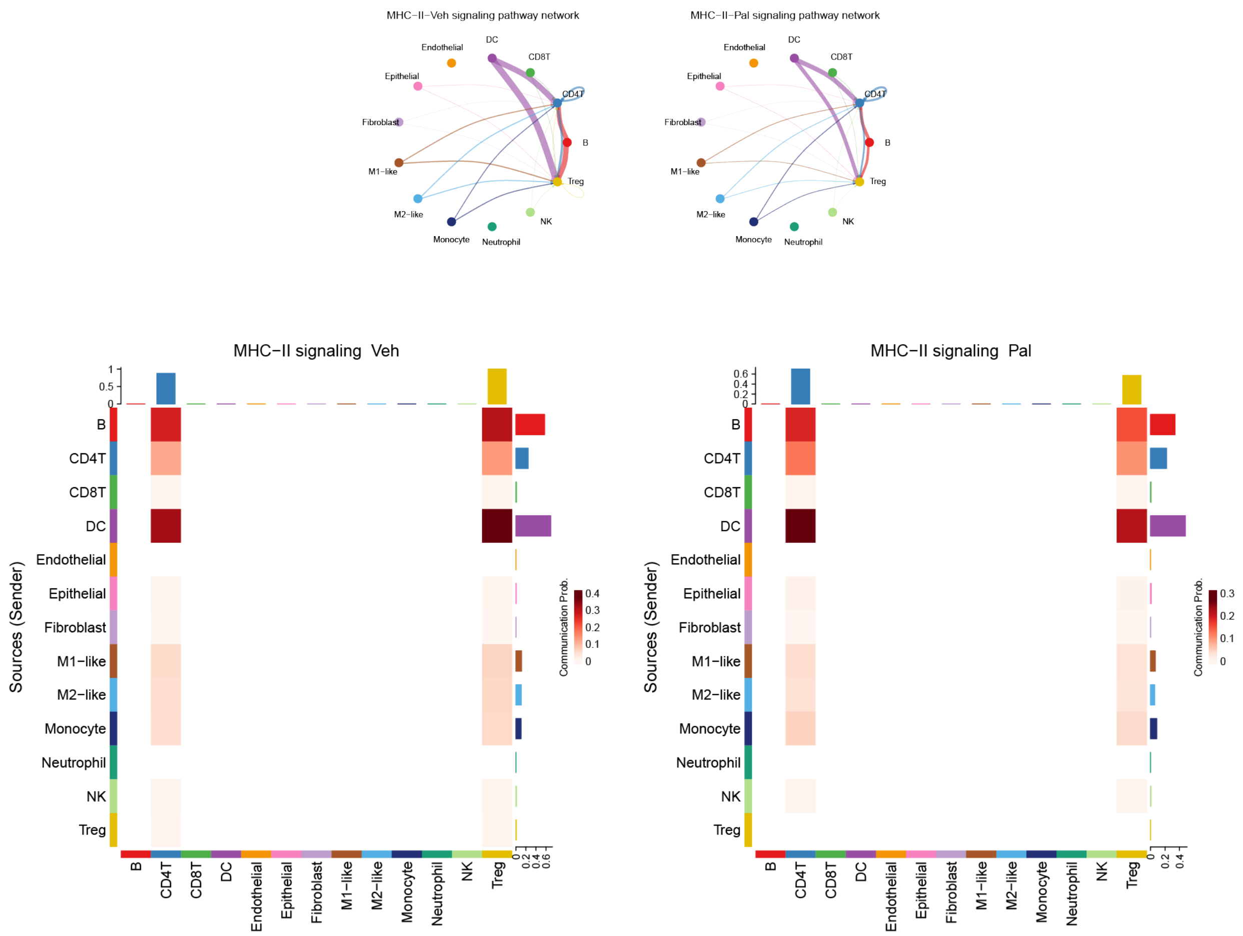

Fig. S11. Analysis of MHC-II pathway interactions in lymphocytes.

(**A**) MHC-II pathway interactions in lymphoid cells.

A

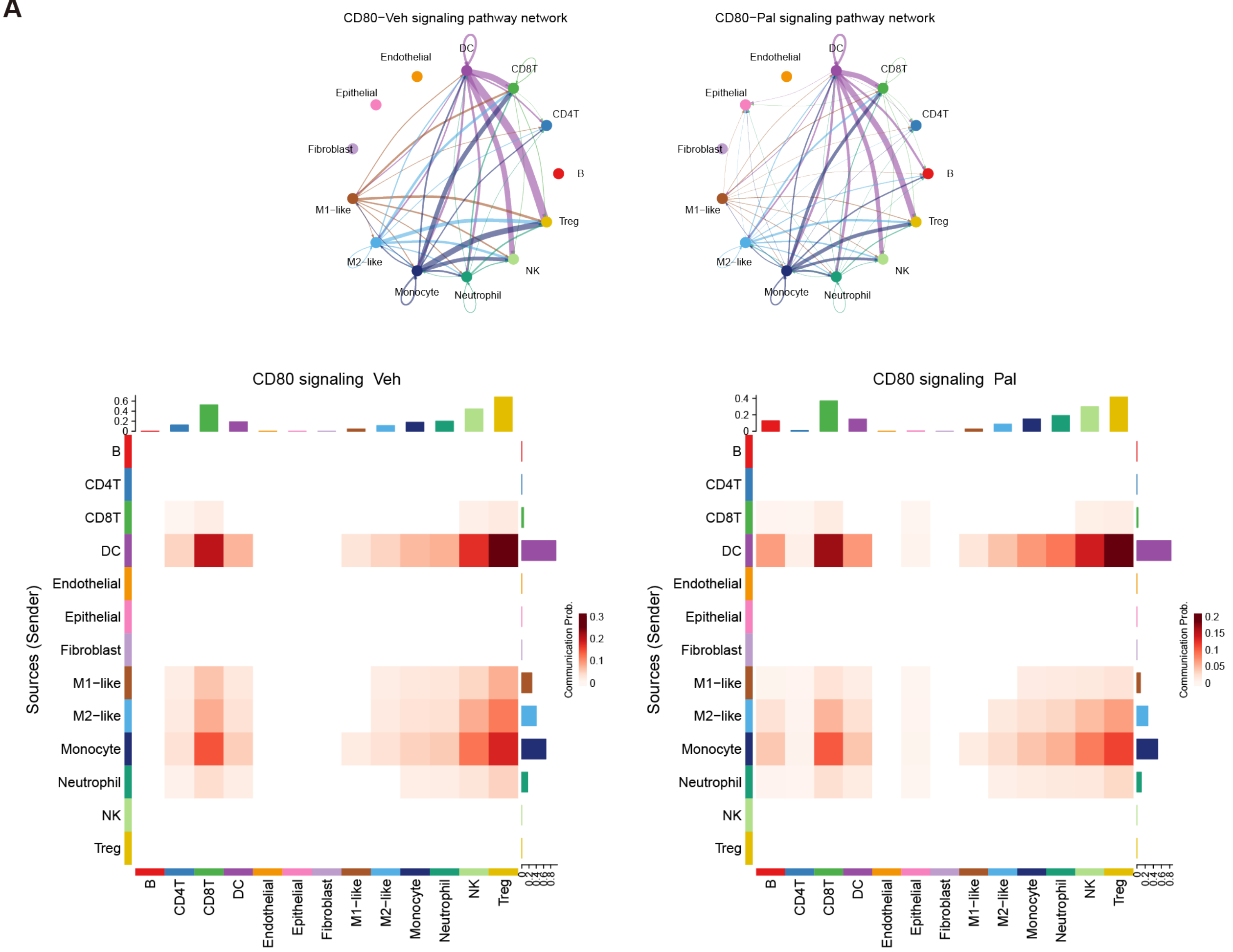

Fig. S12. Analysis of CD80 pathway activity in lymphoid cells.

(**A**) CD80 pathway interactions in lymphoid cells.

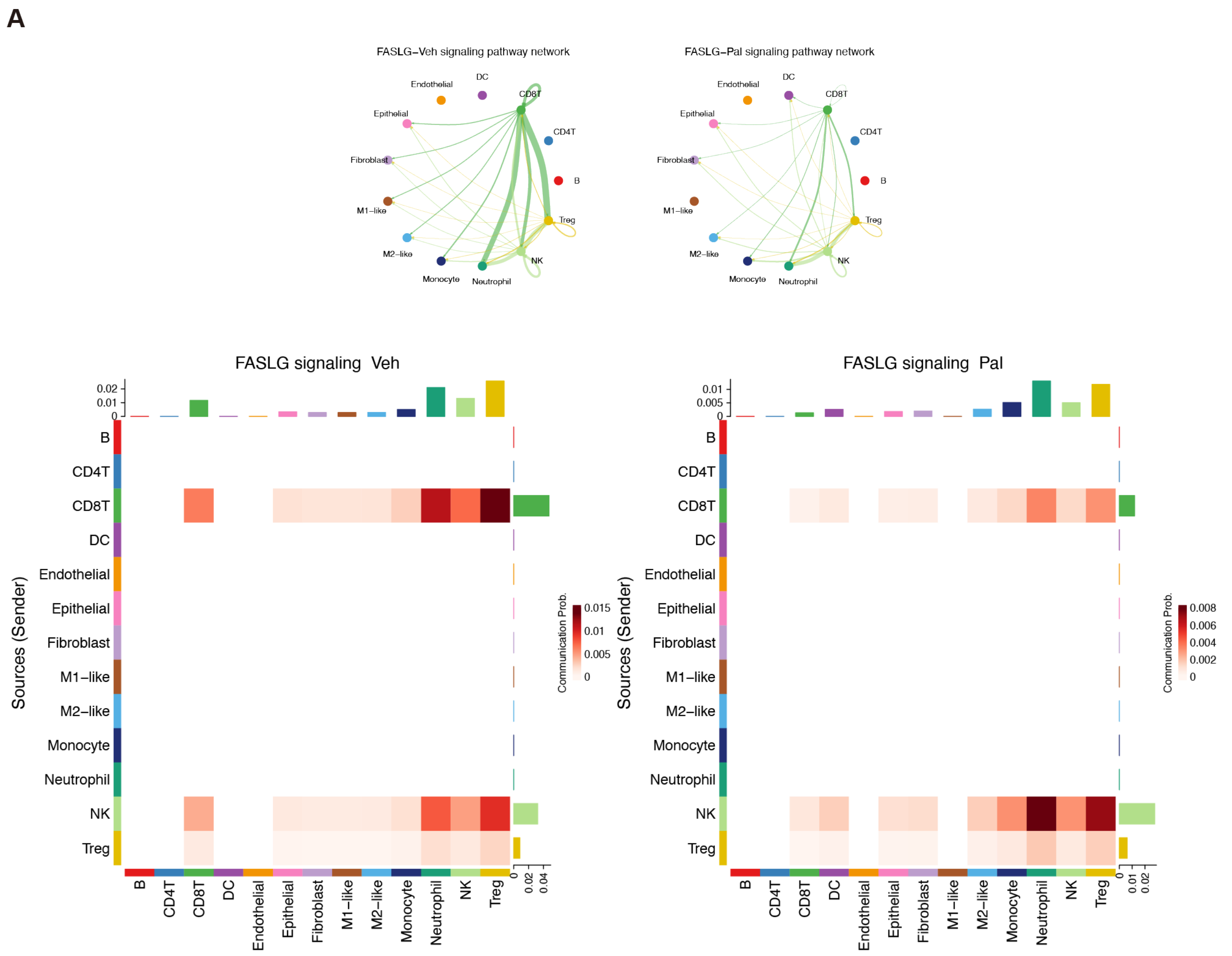

Fig. S13. Communication analysis of the FASLG pathway in lymphoid cells.

(**A**) FASLG pathway interactions in lymphoid cells.

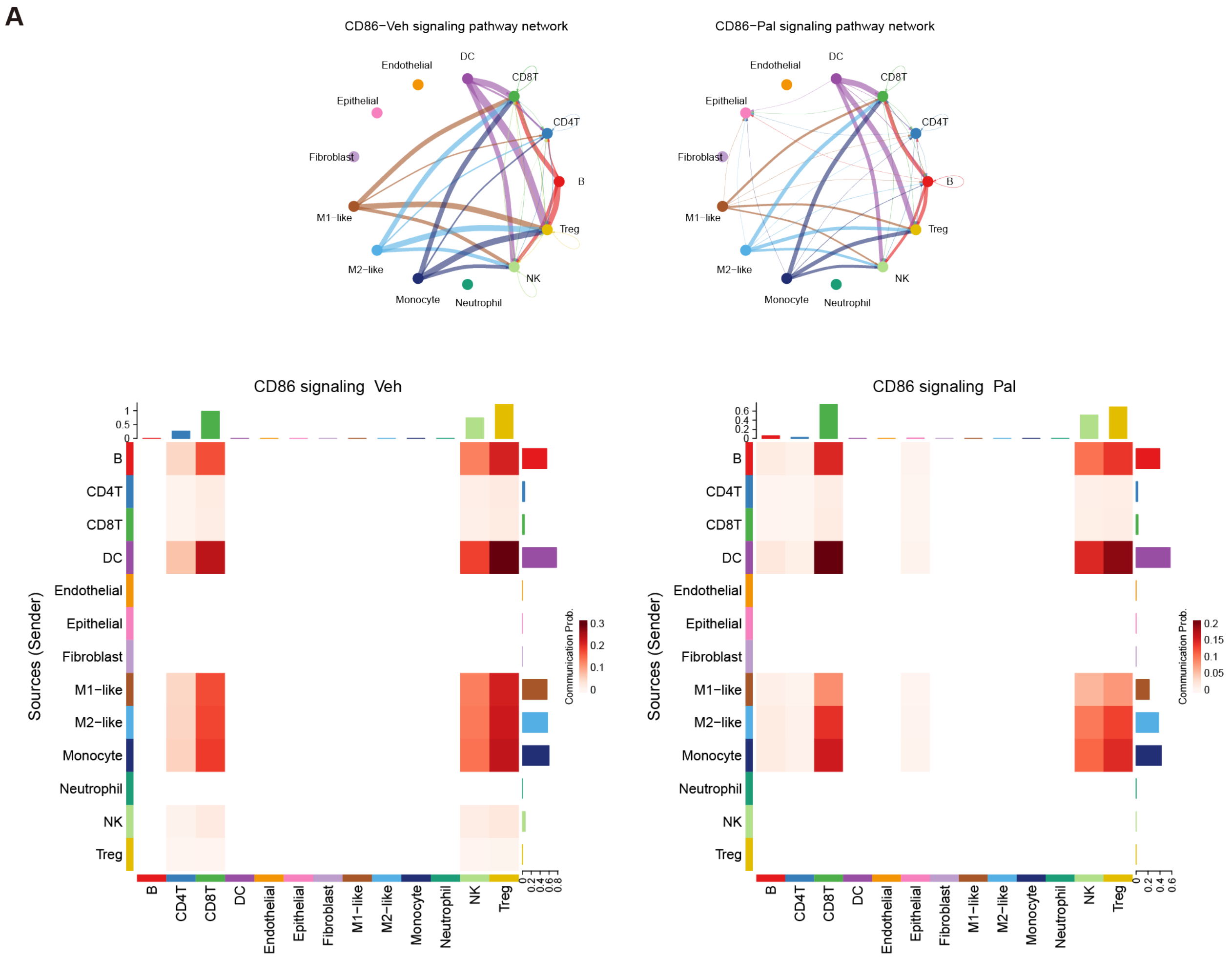

Fig. S14. Analysis of CD86 pathway activity in lymphoid cells.

(**A**) CD86 pathway interactions in lymphoid cells

A

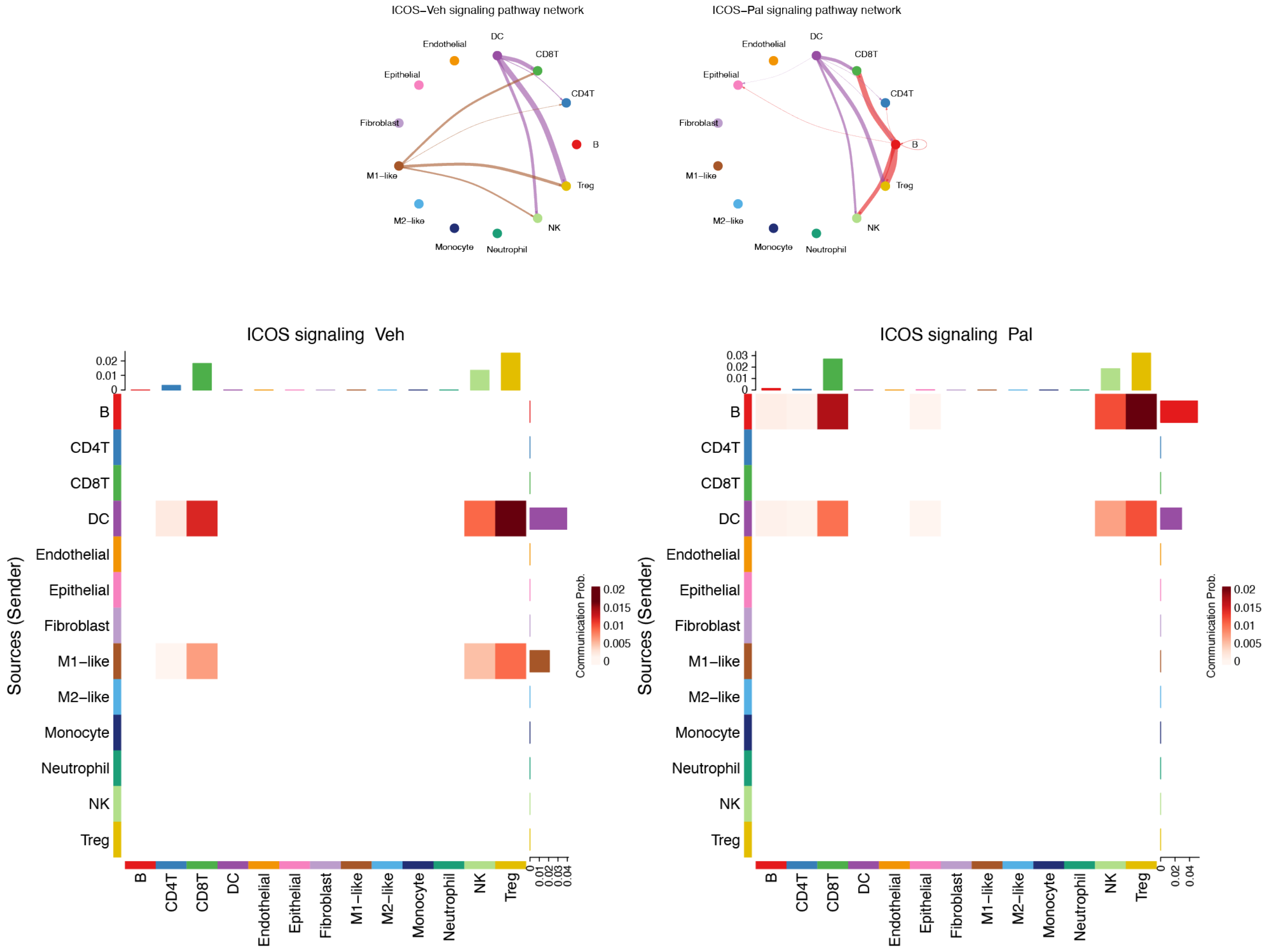

Fig. S15 . Analysis of ICOS pathway activity in lymphoid cells.

(**A**) ICOS pathway interactions in lymphoid cells

A

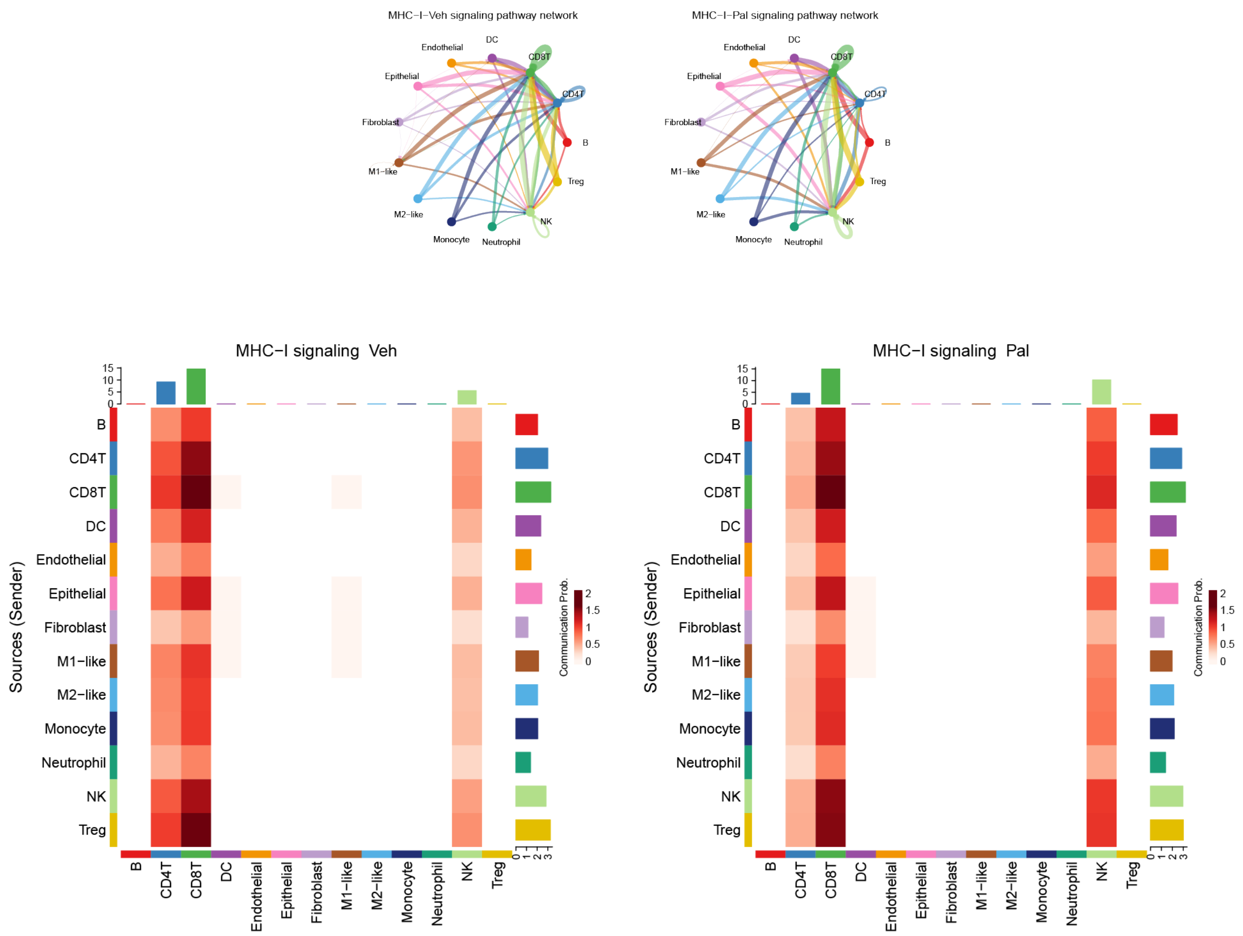

Fig. S16. Analysis of MHC-I pathway interactions in lymphoid cells.

(**A**) MHC-I pathway interactions in lymphoid cells.

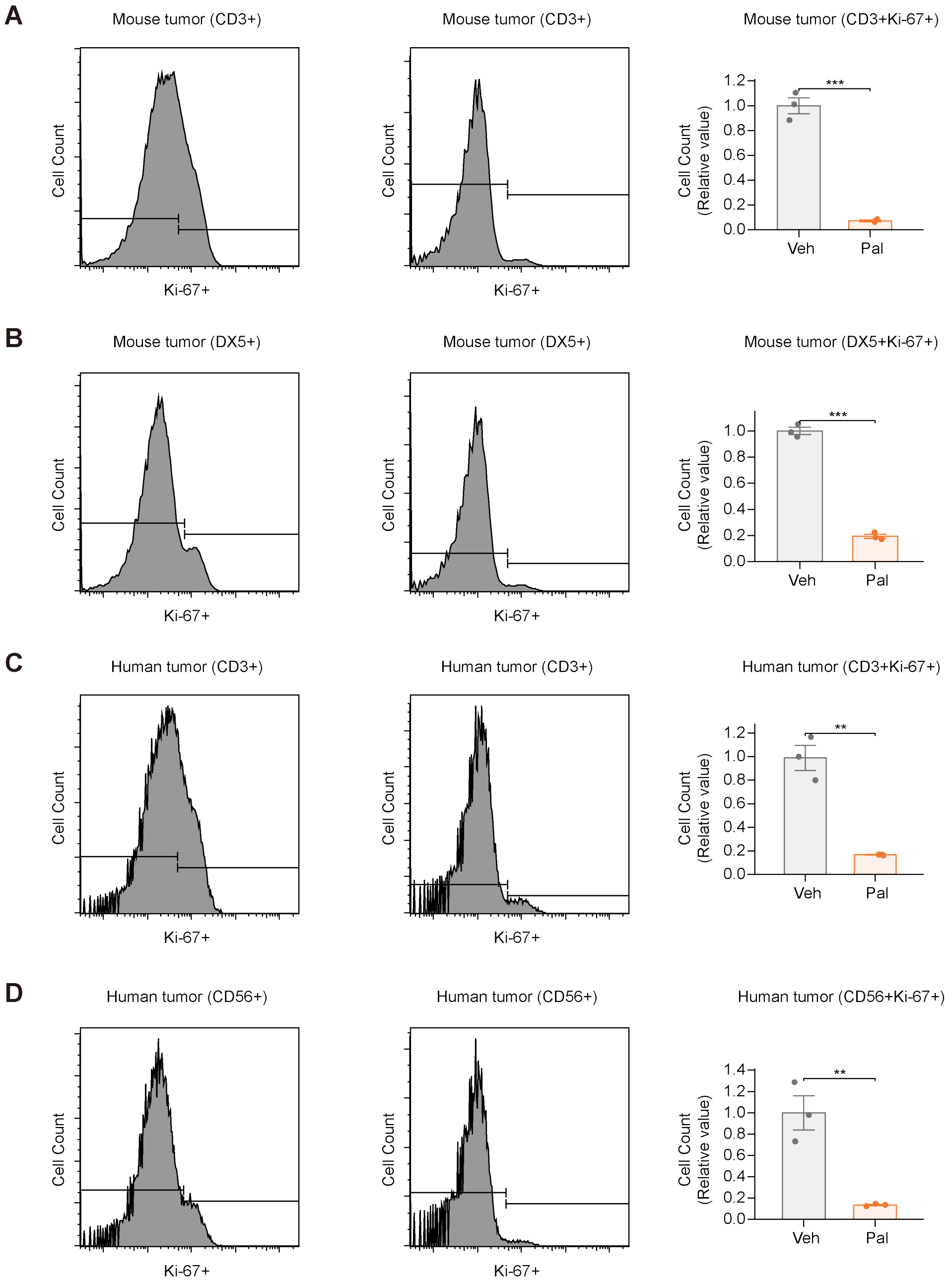

Fig. S17. Flow cytometry analysis of mouse and human tumor tissues.

(**A**–**D**) The expression of Ki-67 in T and NK cells was reduced after Pal treatment.

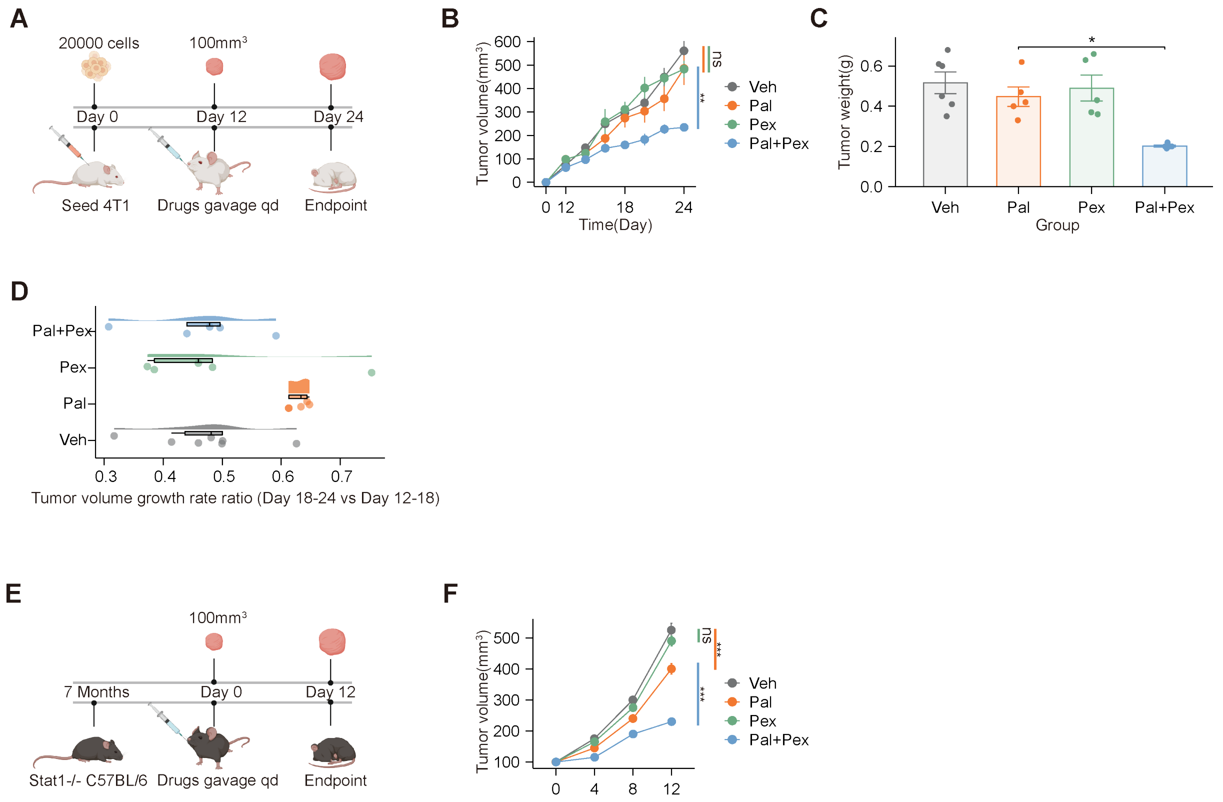

Fig. S18. Pal combined with Pex inhibited tumor growth in 4T1 mouse model.

(**A**) 4T1 breast cancer cells derived from BALB/c mice were orthotopically implanted into the third pair of mammary fat pads on the right side of 20 female BALB/c mice. On day 12, when the tumor volume reached 100 mm³, the mice were administered Pal or Pex by gavage. The experiment was terminated on day 24.

(**B** and **C**) Pex enhanced the killing effect of Pal on mouse tumors.

(**D**) The tumor growth rate ratio of mouse 4T1 tumors.

(**E**) When the tumor volume in Stat1$^{-/-}$ C57BL/6 mice reached approximately 100 mm³, the mice were administered Pal or Pex by oral gavage. The experiment was terminated on day 12.

(**F**) The tumor volume of Stat1-/- mouse tumors.

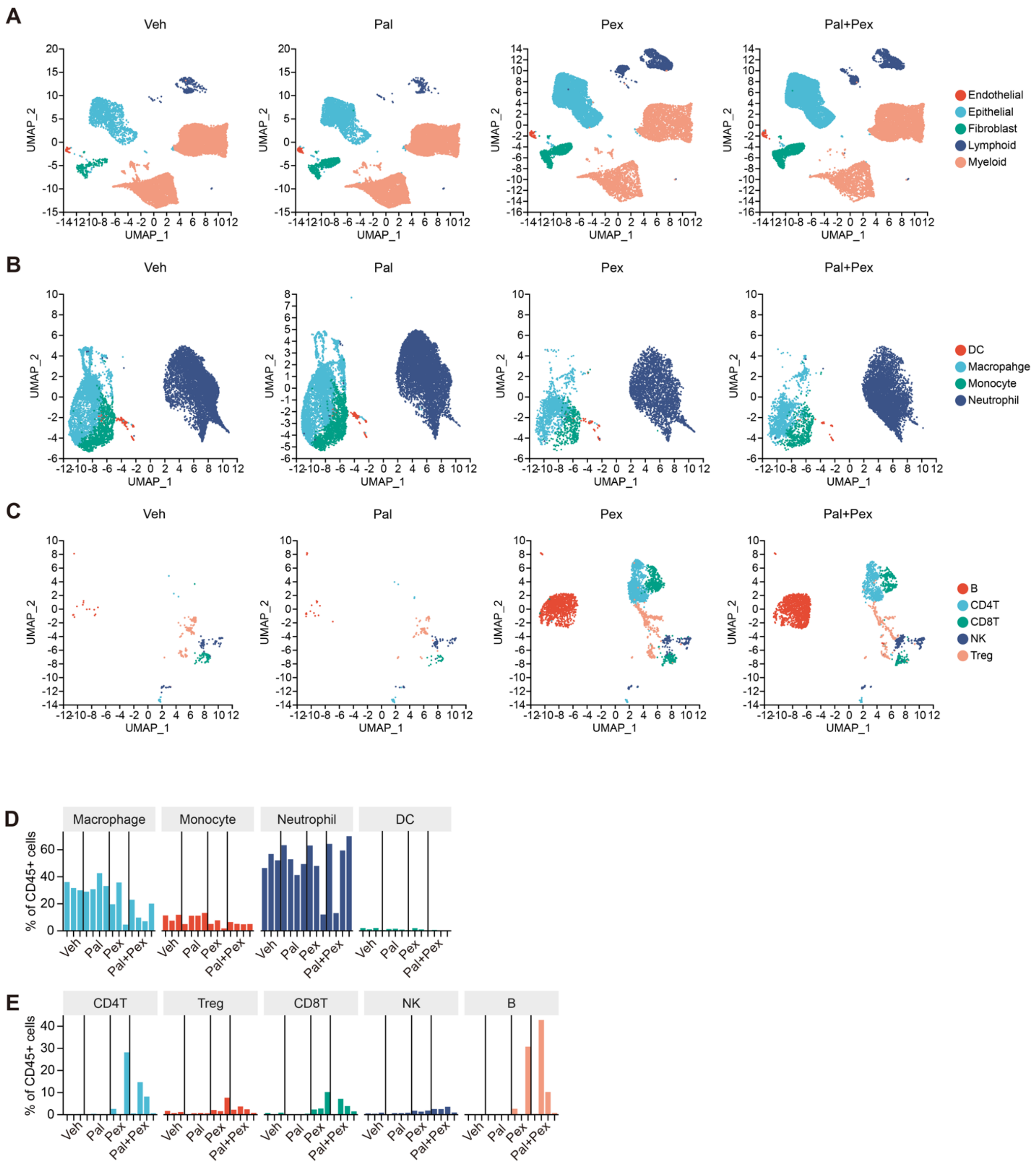

Fig. S19. UMAP of tumor cells.

(**A-C**) UMAP of tumor cells.

(**D**) The Pex intervention reduced the number of myeloid cells.

(**E**) The Pex intervention increased the number of lymphoid cells.

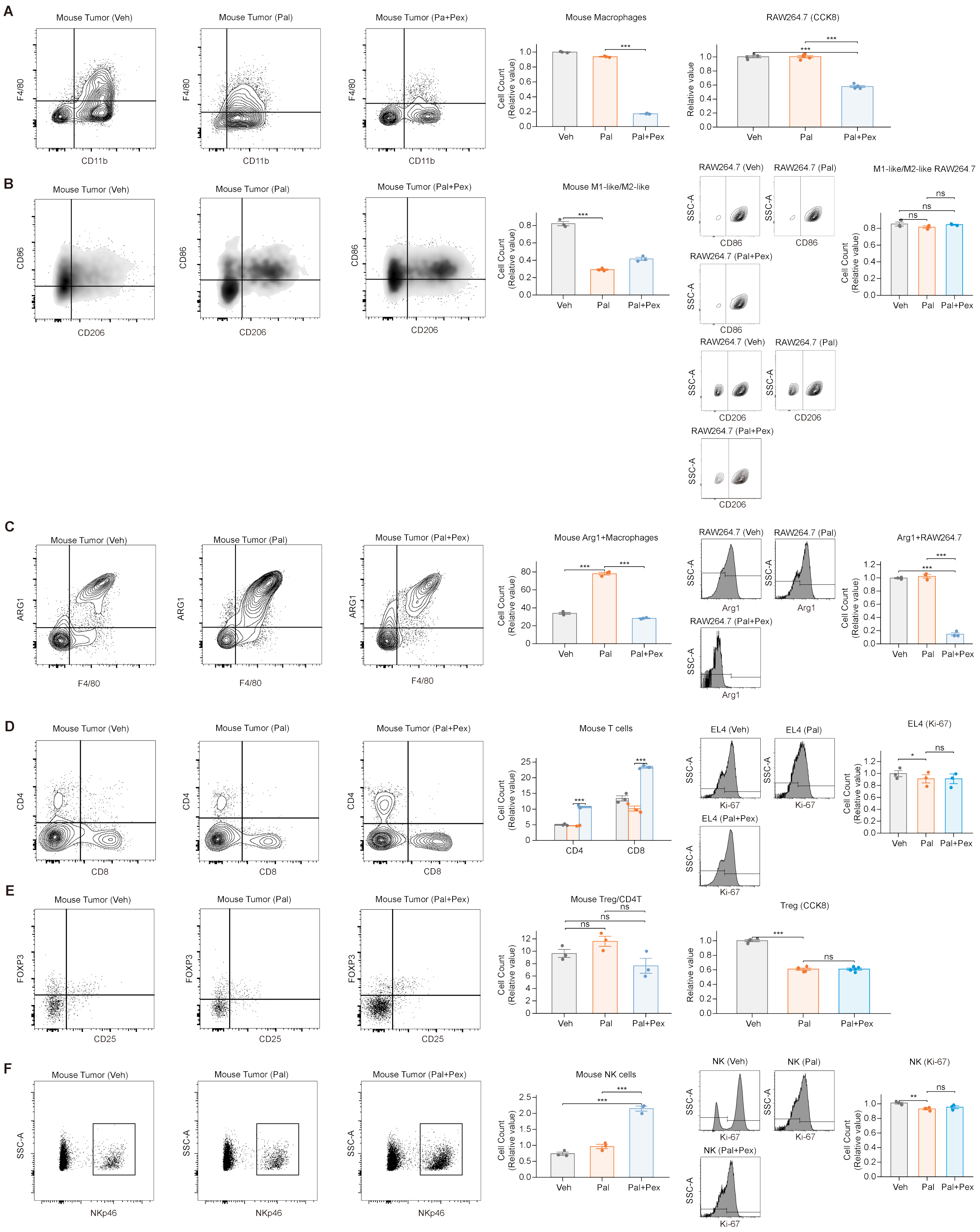

Fig. S20. Flow cytometry analysis of mouse tumor tissues, RAW264.7 cells, EL4 cells and NK cells.

(**A**–**C**) Compared with the Veh group, the Pal group exhibited a decrease in M1-like macrophages and an increase in M2-like and ARG1^{+} macrophages, whereas the Pal+Pex group showed an overall reduction in macrophage numbers in the mouse TME. In contrast, in vitro treatment of RAW264.7 macrophages with Pal did not affect cell viability as assessed by CCK-8 assays, nor did it alter the expression of ARG1, CD86, or CD206. Pex treatment reduced macrophage numbers and attenuated ARG1 expression in vivo.

(**D**–**F**) Compared with those in the Veh group, the numbers of CD4+ T cells, Tregs, CD8+ T cells, and NK cells did not change significantly in the Pal group, but increases in the numbers of CD4+ T cells, CD8+ T cells and NK cells were observed in the Pal+Pex group in the mouse TME. In contrast, in vitro assays revealed that Pal

reduced the viability of primary murine Tregs as assessed by CCK-8 assays and modestly decreased Ki-67 expression in EL4 cells and primary murine NK cells, while Pex alone did not exert significant effects on cell viability or proliferation in these cell types.

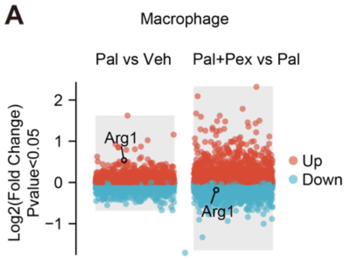

Fig. S21. Pal and Pex upregulated and downregulated the expression of Arg1.

(**A**) Transcriptome analysis of macrophages revealed that Pal and Pex upregulated and downregulated the expression of Arg1.

A

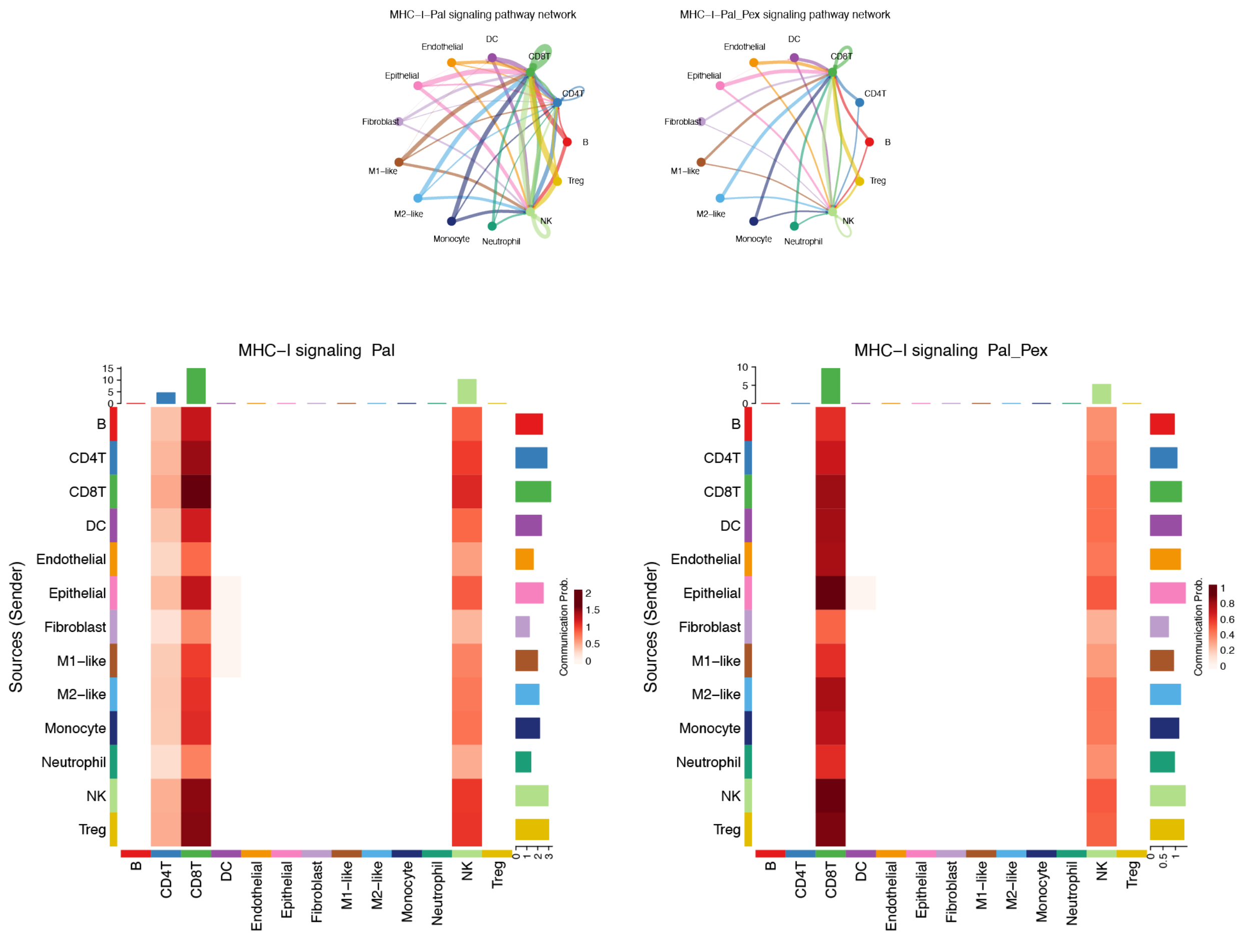

Fig. S22. Analysis of MHC-I pathway interactions in lymphoid cells.

(**A**) MHC-I pathway interactions in lymphoid cells

A

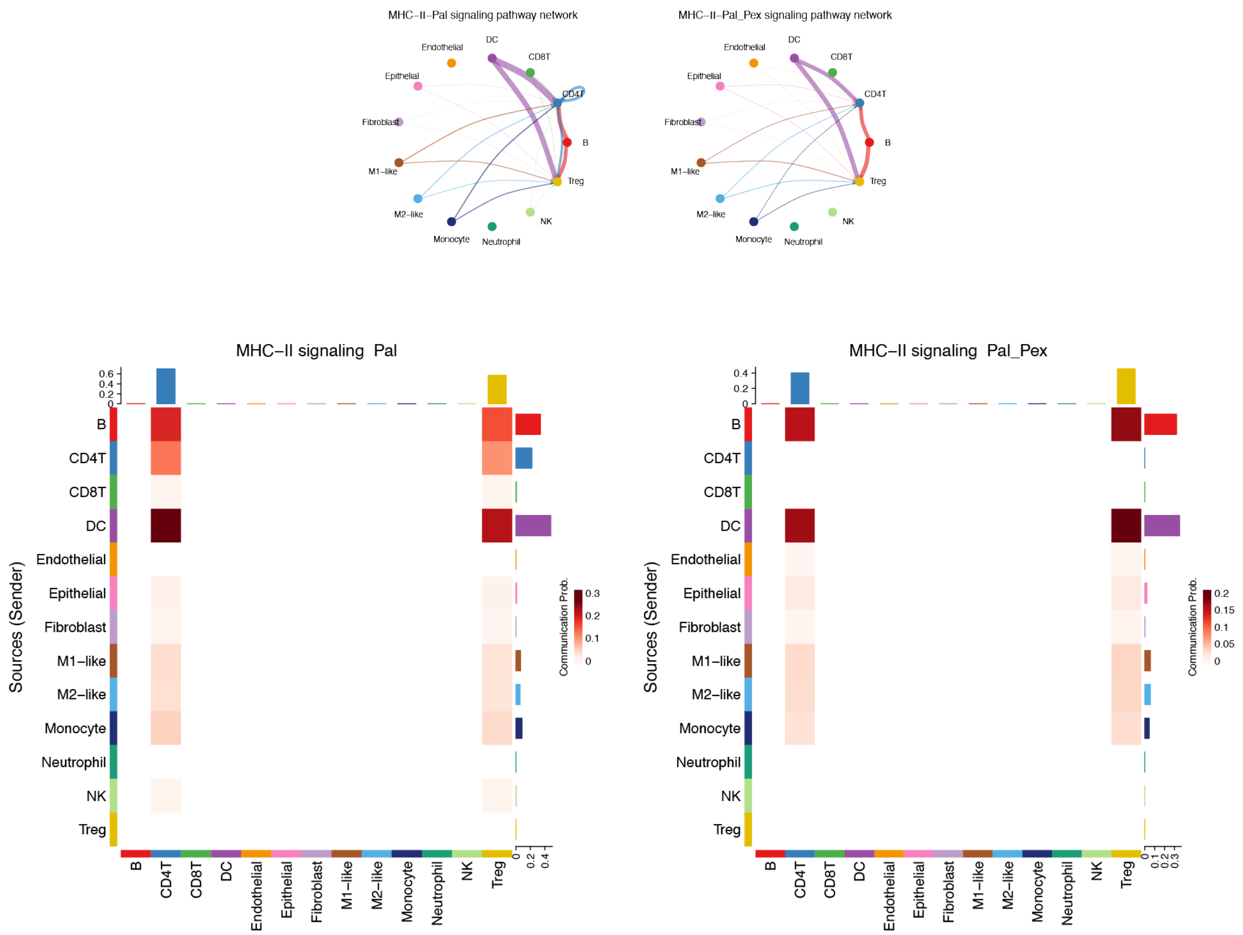

Fig. S23. Analysis of MHC-II pathway interactions in lymphoid cells.

(**A**) MHC-II pathway interactions in lymphoid cells

A

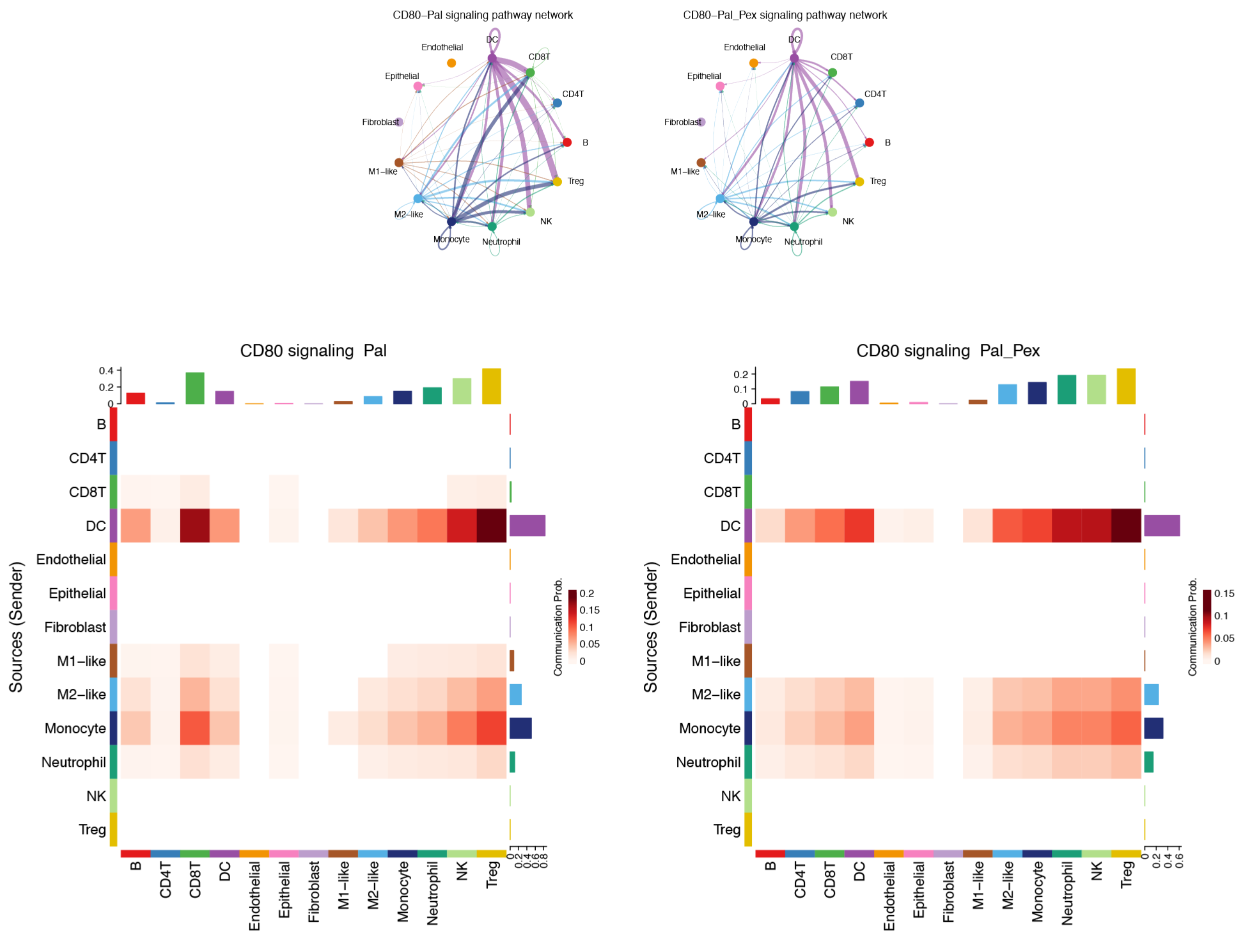

Fig. S24. Analysis of CD80 pathway activity in lymphoid cells.

(**A**) CD80 pathway interactions in lymphoid cells

A

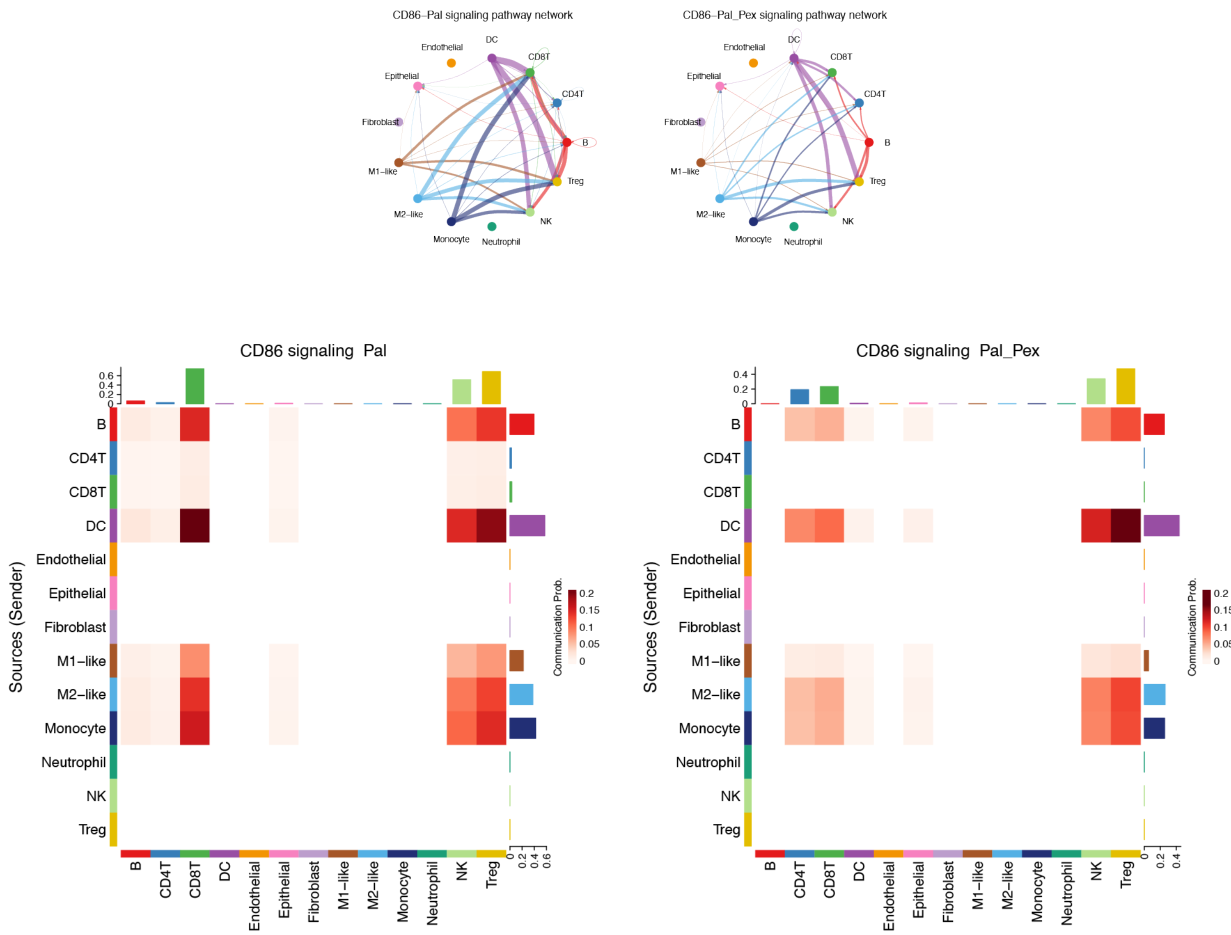

Fig. S25. Analysis of CD86 pathway activity in lymphoid cells.

(**A**) CD86 pathway interactions in lymphoid cells

A

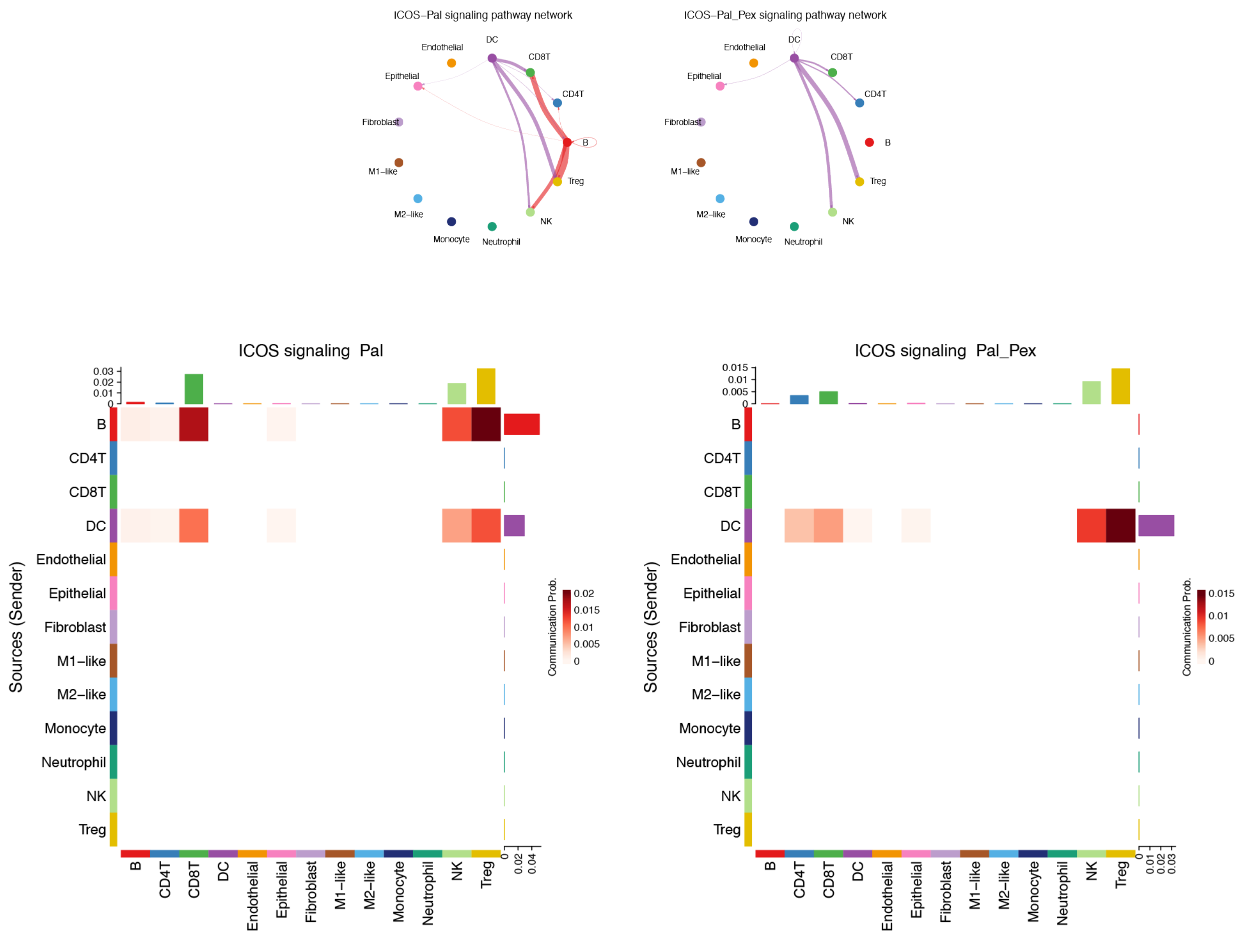

Fig. S26. Analysis of ICOS pathway activity in lymphoid cells.

(**A**) ICOS pathway interactions in lymphoid cells

A

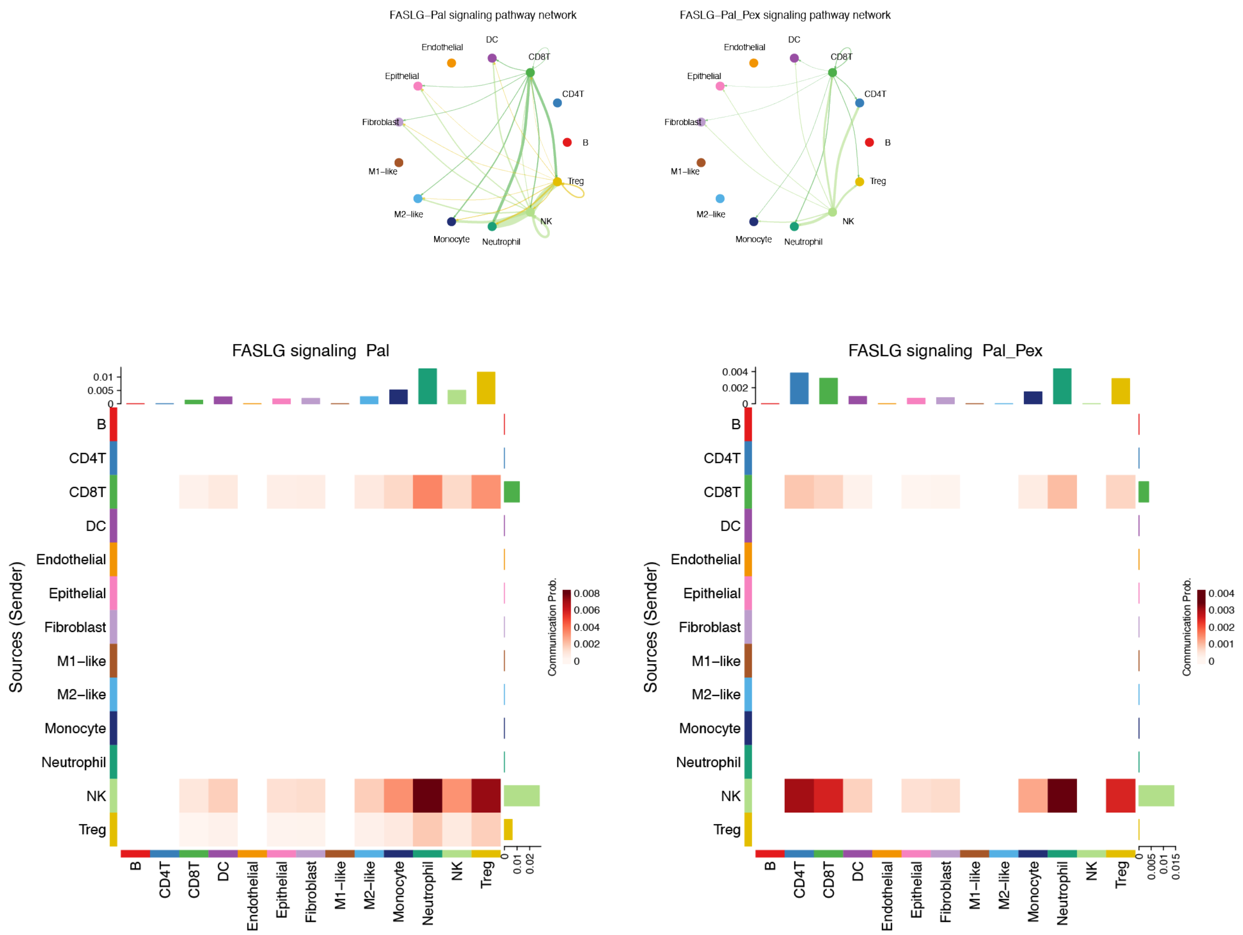

Fig. S27. Communication analysis of the FASLG pathway in lymphoid cells.

(**A**) FASLG pathway interactions in lymphoid cells

A

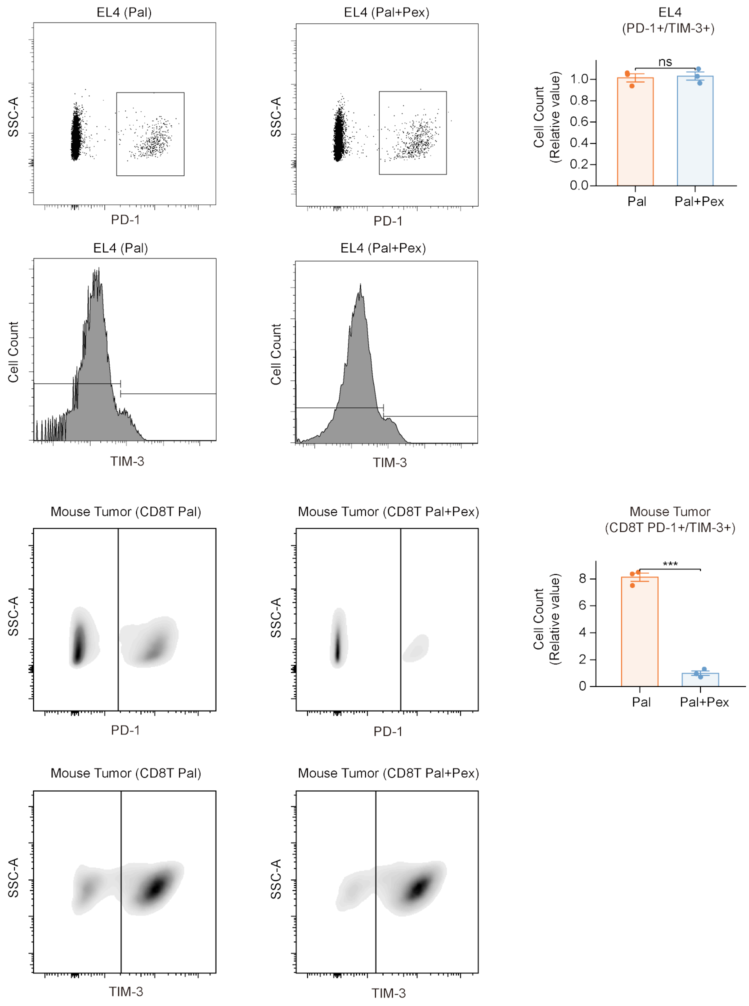
EL4 (Pal)
EL4 (Pal+Pex)
SSC-A
PD-1
EL4
(PD-1+/TIM-3+)
ns
Cell Count
(Relative value)
1.0
0.8
0.6
0.4
0.2
0.0
Pal
Pal+Pex
Cell Count
TIM-3
Mouse Tumor (CD8T Pal)
Mouse Tumor (CD8T Pal+Pex)
Mouse Tumor
(CD8T PD-1+/TIM-3+)
***
8
6
4
2
0
TIM-3

B

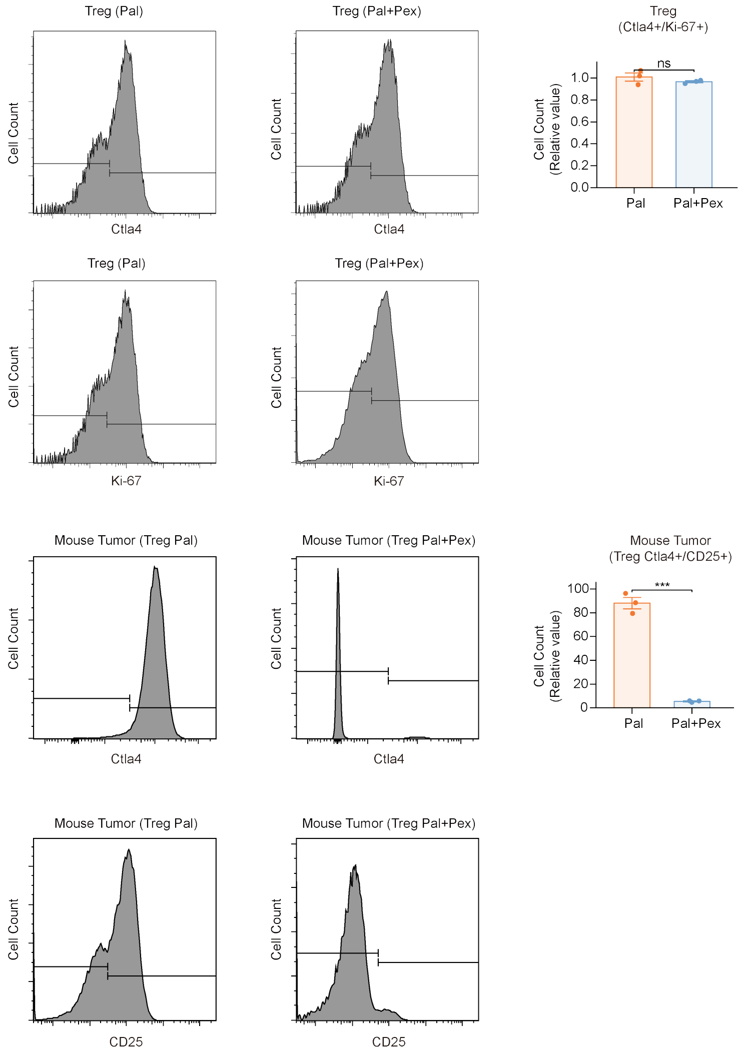
Treg (Pal)
Treg (Pal+Pex)
Cell Count
Ctla4
Treg
(Ctla4+/Ki-67+)
ns
Cell Count
(Relative value)
1.0
0.8
0.6
0.4
0.2
0.0
Pal
Pal+Pex
Ki-67
Mouse Tumor (Treg Pal)
Mouse Tumor (Treg Pal+Pex)
Mouse Tumor
(Treg Ctla4+/CD25+)
***
100
80
60
40
20
0
CD25

Fig. S28. Flow cytometry analysis of mouse tumor tissues, CD8$^+$ T cells, and Tregs.

(**A**) Exhaustion-associated markers including Pdcd1 and Havcr2 (TIM-3) showed the most significant changes in mouse TME. In contrast, in vitro Pal treatment did not alter Pdcd1 or TIM-3 expression in activated EL4 cells.

(**B**) Pex reduced Ctla4 and Il2ra (CD25) expression on Tregs in mouse TME. In contrast, in vitro Pal treatment did not alter Ctla4 or Ki-67 expression in activated primary murine Treg cells.

A

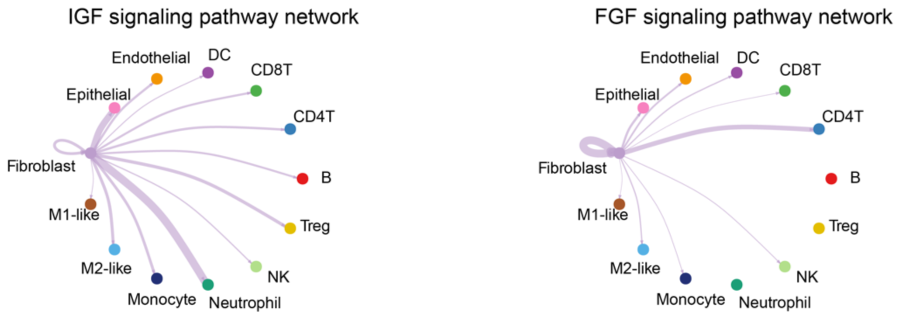

B

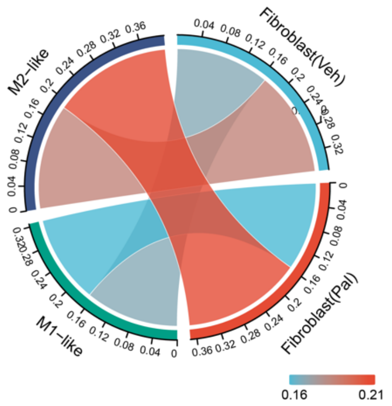

Fig. S29. Communication diagram between fibroblasts and lymphoid cells via the IGF pathway.

(**A**) Cell communication analysis indicated that intercellular communication involving IGF and FGF within the TME is primarily mediated by fibroblasts.

(**B**) The probability of IGF and FGF communication between fibroblasts and M2-like macrophages increased, while the probability of communication between fibroblasts and M1-like macrophages decreased

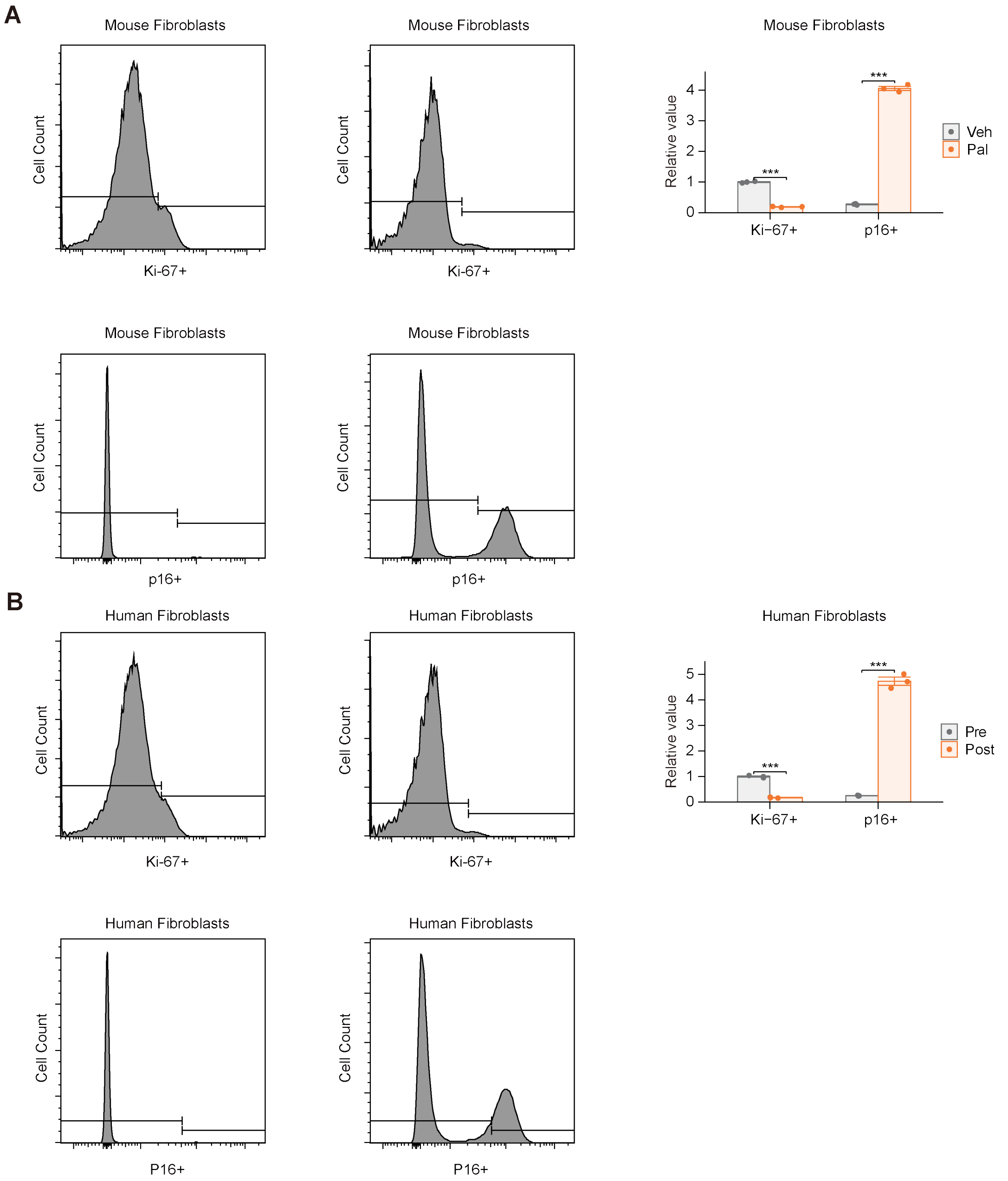

Fig. S30. Flow cytometry analysis of mouse and human fibroblasts.

(**A**) Pal suppressed Ki-67 expression in fibroblasts and upregulated the senescence marker p16.

(**B**) Fibroblasts isolated from HR+/HER2− breast cancer tissues from patients treated with Pal also revealed reduced Ki-67 expression and increased P16 levels.

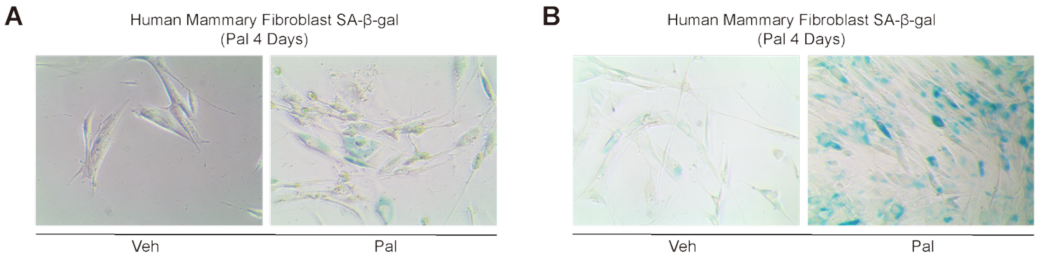

Fig. S31. Pal intervention induced senescence in HMFs after four days, as evidenced by β-Galactosidase assays.

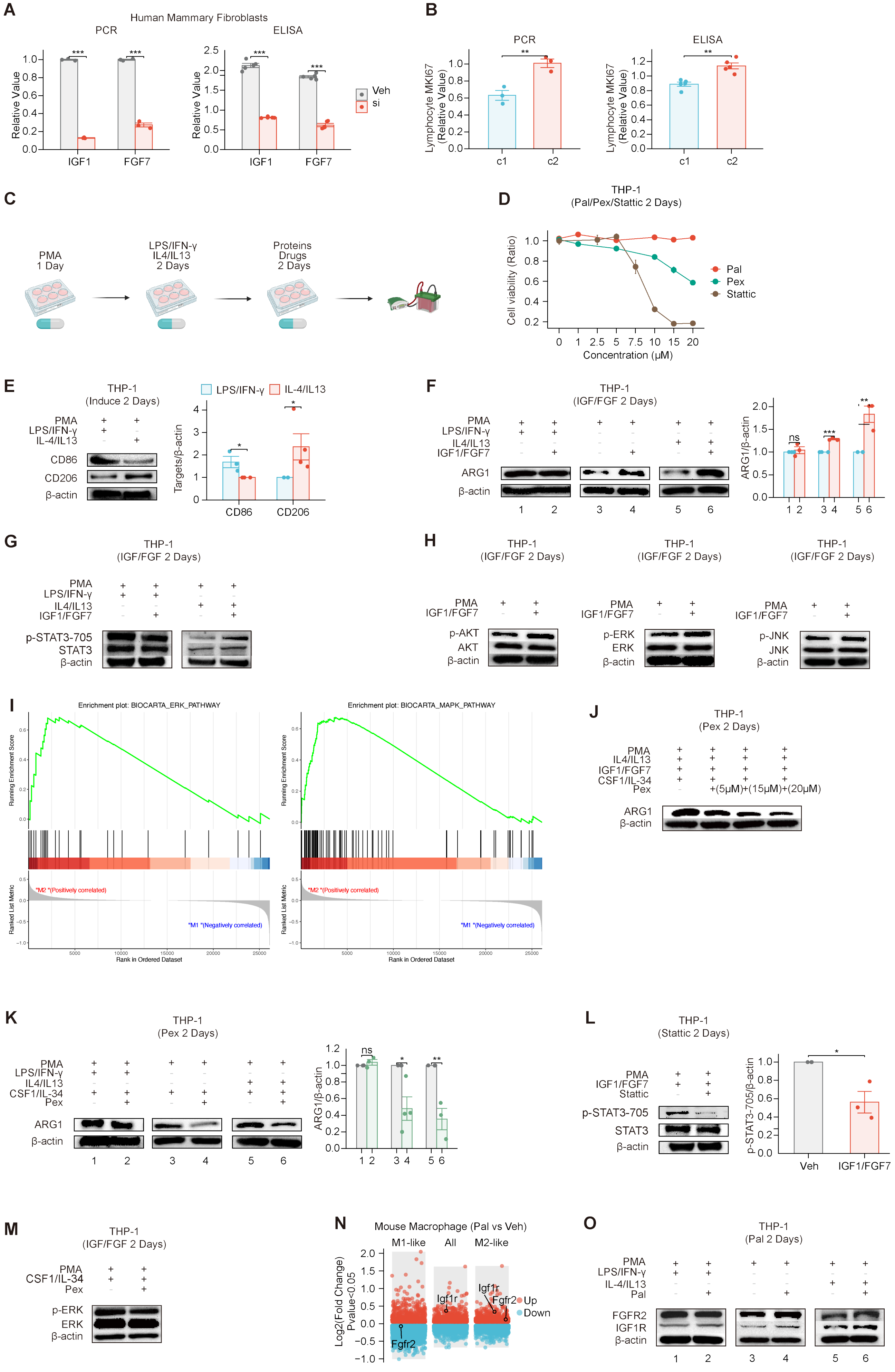
A
Human Mammary Fibroblasts
PCR
ELISA
Relative Value
IGF1
FGF7
Veh
si
B
PCR
ELISA
Lymphocyte MKI67 (Relative Value)
c1
c2
C
PMA 1 Day
LPS/IFN-γ IL4/IL13 2 Days
Proteins Drugs 2 Days
D
THP-1 (Pal/Pex/Stattic 2 Days)
Cell viability (Ratio)
Concentration (μM)
Pal
Pex
Stattic
E
THP-1 (Induce 2 Days)
PMA
LPS/IFN-γ
IL-4/IL13
CD86
CD206
β-actin
LPS/IFN-γ
IL-4/IL13
Targets/β-actin
F
THP-1 (IGF/FGF 2 Days)
PMA
LPS/IFN-γ
IL4/IL13
IGF1/FGF7
ARG1
β-actin
ARG1/β-actin
G
THP-1 (IGF/FGF 2 Days)
p-STAT3-705
STAT3
β-actin
H
THP-1 (IGF/FGF 2 Days)
p-AKT
AKT
p-ERK
ERK
p-JNK
JNK
I
Enrichment plot: BIOCARTA_ERK_PATHWAY
Enrichment plot: BIOCARTA_MAPK_PATHWAY
Running Enrichment Score
Ranked List Metric
"M2 "(Positively correlated)
"M1 "(Negatively correlated)
Rank in Ordered Dataset
J
THP-1 (Pex 2 Days)
PMA
IL4/IL13
IGF1/FGF7
CSF1/IL-34
Pex
+(5μM)+(15μM)+(20μM)
ARG1
β-actin
K
THP-1 (Pex 2 Days)
PMA
LPS/IFN-γ
IL4/IL13
CSF1/IL-34
Pex
ARG1
β-actin
ARG1/β-actin
L
THP-1 (Stattic 2 Days)
PMA
IGF1/FGF7
Stattic
p-STAT3-705
STAT3
β-actin
p-STAT3-705/β-actin
Veh
IGF1/FGF7
M
THP-1 (IGF/FGF 2 Days)
PMA
CSF1/IL-34
Pex
p-ERK
ERK
β-actin
N
Mouse Macrophage (Pal vs Veh)
M1-like
All
M2-like
Log2(Fold Change) Pvalue<0.05
Igf1r
Fgfr2
Up
Down
O
THP-1 (Pal 2 Days)
PMA
LPS/IFN-γ
IL-4/IL13
Pal
FGFR2
IGF1R
β-actin

Fig. S32. Pal promotes fibroblast secretion of IGF1 and FGF7 which stimulate macrophages to express ARG1 and suppress lymphocyte viability.

(**A**) Knockdown of IGF1 and FGF7 at the RNA and protein levels reduced the expression of IGF1 and FGF7 in HMFs.

(**B**) Arginine restored the viability of HBPLs at both the RNA and protein levels.

(**C**) THP-1 cells were treated with 100 ng/ml PMA for one day to induce their differentiation into macrophages and promote their adhesion. The macrophages were then treated with 100 ng/ml LPS and 20 ng/ml IFN-γ for two days to induce M1 macrophage polarization. The macrophages were treated with 20 ng/ml IL-4 and 20 ng/ml IL13 for two days to induce M2 macrophage polarization. After induction, the macrophages were treated with the corresponding recombinant proteins or drugs for two days.

(**D**) The CCK-8 results revealed that, based on the proliferation of THP-1 cells induced by PMA (100 ng/ml, 1 day), the concentration of Pex was determined to be 5 μM, and the concentration of Stattic was determined to be 5 μM.

(**E**) The WB results obtained after THP-1 cell induction are shown.

(**F**) THP-1 cells were treated with 50 ng/ml IGF1 and 50 ng/ml FGF7 for two days, and IGF1 and FGF7 upregulated ARG1 expression in unpolarized macrophages and M2 macrophages.

(**G**) IGF1 and FGF7 induced the phosphorylation of the STAT3 protein at site 705 in M2-polarized macrophages, whereas the STAT3 protein in M1 macrophages remained unphosphorylated.

(**H**) IGF1 and FGF7 slightly induced the phosphorylation of AKT, ERK, and JNK proteins in macrophages.

(**I**) GSEA revealed that the ERK and MAPK signaling pathways were enriched in M2-like macrophages.

(**J**) Pex inhibited the protein expression of ARG1 in unpolarized macrophages and M2 macrophages.

(**K**) THP-1 cells were treated with 50 ng/ml CSF1, 50 ng/ml IL-34, or 5 μM Pex for two days in vitro, and Pex downregulated ARG1 expression in unpolarized macrophages and M2 macrophages.

(**L**) THP-1 cells were treated with 5 μM Stattic (a STAT3 phosphorylation inhibitor) for two days, and the WB results revealed that Stattic inhibited the phosphorylation of the STAT3 protein at tyrosine 705.

(**M**) Pex slightly inhibited the phosphorylation of the ERK protein in macrophages.

(**N**) Transcriptomic data indicating that Pal upregulated Igf1r in macrophages and both Igf1r and Fgfr2 in M2-like macrophages.

(**O**) WB results indicating that Pal upregulated the expression of IGF1R and FGFR2 in unpolarized macrophages and M2-like macrophages.

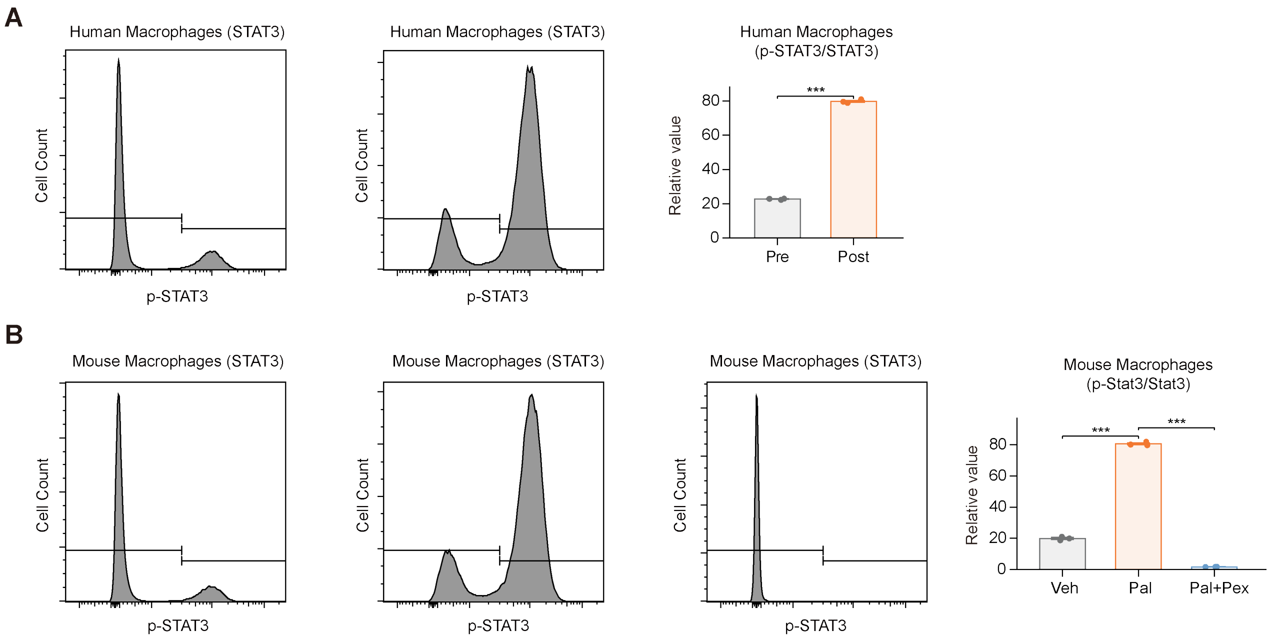

Fig. S33. Flow cytometry analysis of mouse and human Macrophages.

(**A** and **B**) Increased phosphorylation of STAT3 at tyrosine 705 in macrophages within tumor tissues from both post-Pal HR+/HER2− breast cancer patients and mouse models.

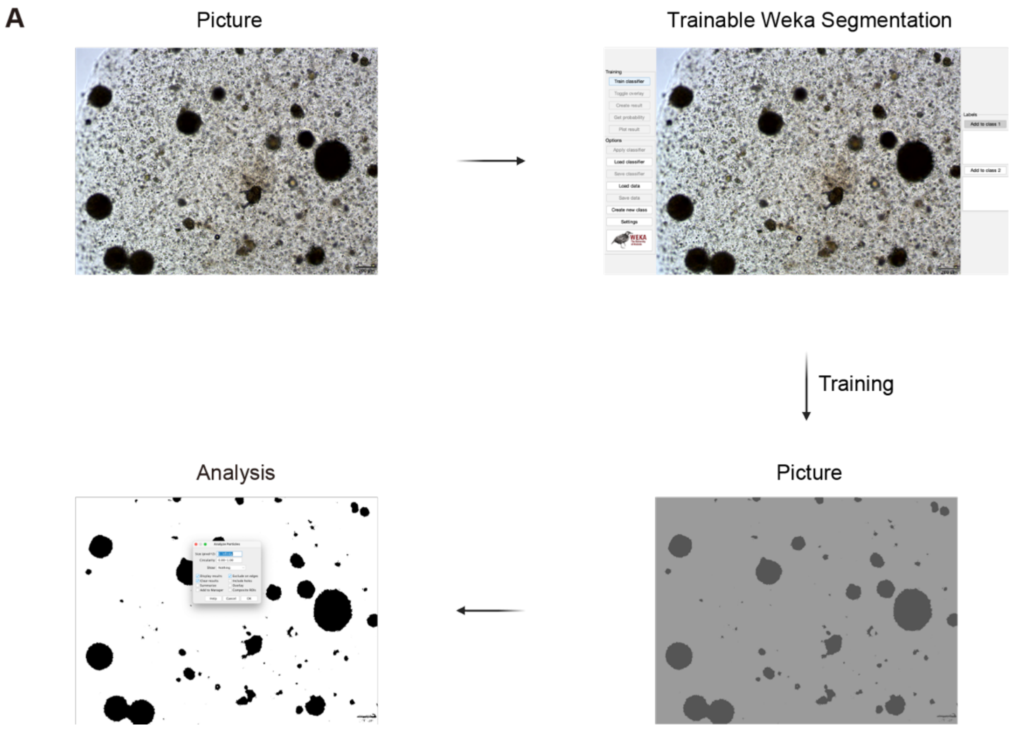

Fig. S34. 3D Cultured cells statistical step diagram.

(**A**) 3D Cultured cells statistical step diagram.
