# Supplementary material for: CSF1R inhibitors mitigate CDK4/6 inhibitor-induced immunosuppression to increase antitumor immunity in HR+/HER2− breast cancer
Source: Oncogene. 2026 Apr 15;45(21):1970–87. doi: 10.1038/s41388-026-03786-w (PMC13190250; doi:10.1038/s41388-026-03786-w)
Supplement: Supplementary file 2 — Supplementary Data (mIHC) [file 41388_2026_3786_MOESM2_ESM.docx]

**Antibody**

**Human**

CD68 (76437, CST)

CD86 (269587, ABCAM)

CD206 (24595, CST)

ARG1 (EPR6672(B), ABCAM)

CD11b (SDT-058-44, STARTER)

CD14 (SDT-060-50, STARTER)

CD15 (SDT-R009, STARTER)

CD4 (48274, ABCAM)

CD8 (85336, CST)

FOXP3 (98377, CST)

PAN-CK (7753, ABCAM)

**Mouse**

CD86 (19589, CST)

CD163 (182422, ABCAM)

CD11b (133357, ABCAM)

LY6G (87048, CST)

LY6C (HA500087, ABCAM)

F4/80 (70076, CST)

CD4 (183685, ABCAM)

CD8 (209775, ABCAM)

FOXP3 (12653, CST)

**Laboratory Equipment**

| Name | Macufaacturer | Model |
| --- | --- | --- |
| Dehydrator | leica | Leica ASP300S |
| Embedding Machine | leica | HistoCore Arcadia H |
| Pathology Slicer | leica | HistoCore BIOCUT |
| Cryostat | leica | HistoCore Arcadia C |
| Tissue Spreader | leica | HI1210 |
| Slide Dryer | leica | HI1220 |
| Slides | Jiangsu Shitai Experimental Equipment | 80312-3161 |
| Cover Slips | Nantong Meiweide Life Science | CS01-2450 |
| Microwave Oven | Midea | M1-L3B |
| Decoloring Shaker | Haimen Qilinbeier Instrument Manufacturing | T8-1000 |
| Vortex Mixer | Haimen Qilinbeier Instrument Manufacturing | VORTEX5 |
| Immunohistochemistry Pen | ImmEdg | H-4000 |
| EP Tubes | BKMAM | BK-CTOP2 |
| Pipette Tips | Code | SF-T-200 |
| Bright Field Scanner | Shunyu | HS6 |
| Pure Water Machine | Millipore | SYNSVR000 |
| Restaining Hematoxylin Staining Machine | leica | ST5020 |
| Sealing Machine | leica | CV5030 |
| Fluorescence Scanner | leica | VERSA8 |
| Microscope | OLYMPUS | CX23 |
| Multilabel Fully Automatic Stainer | leica | BOND RX |

**Main reagents**

| Reagent | Manufacturer | Item Num |
| --- | --- | --- |
| Anhydrous Ethanol | Sinopharm Chemical Reagent | 100092683 |
| Xylene | Sinopharm Chemical Reagent | 1330-20-7 |
| Tween-20  H_2_O_2_  Goat Serum Blocking Solution  Primary Antibody Diluent | Sinopharm Chemical Reagent Zhongshanjinqiao  CWBIO  Zhongshanjinqiao | Tween-20  PV-6001  01380/34021  ZLI-9030D |
| Enzyme-labeled Goat Anti-Rabbit IgG Polymer  Enzyme-labeled Goat Anti-Mouse IgG Polymer | Zhongshanjinqiao  Zhongshanjinqiao | PV-6001  PV-6001 |
| Hematoxylin Staining Solution | Zhuhai Besso Biotechnology | BA4041 |
| Differentiation Solution | Leagene | DH0085 |
| DAB Chromogenic Reagent Kit | Zhongshanjinqiao | ZLI-9018 |
| Fluorescent Dye Diluent | AKOYA | FP1498 |
| 480 Dye | AKOYA | OP-001000 |
| 520 Dye | AKOYA | OP-001001 |
| 620 Dye | AKOYA | OP-001004 |
| 690 Dye | AKOYA | OP-001006 |
| 570 Dye  Anti-fluorescence  Quenching Mounting Medium | AKOYA  Beyotime | OP-001003  PO131 |
