# Supplementary material for: CSF1R inhibitors mitigate CDK4/6 inhibitor-induced immunosuppression to increase antitumor immunity in HR+/HER2− breast cancer
Source: Oncogene. 2026 Apr 15;45(21):1970–87. doi: 10.1038/s41388-026-03786-w (PMC13190250; doi:10.1038/s41388-026-03786-w)
Supplement: Supplementary file 3 — Supplementary Data (Western Blot) [file 41388_2026_3786_MOESM3_ESM.docx]

**Antibody**

p-ERK1-202/204+ERK2-185/187 (AP0974, ABclonal)

ERK1/ERK2 (A16686, ABclonal)

p-AKT-473 (GTX128414, GeneTex)

AKT (9272, Cell Signaling Technology)

p-p38 MAPK-180/182 (GTX133460, GeneTex)

p38 MAPK (8690T, Cell Signaling Technology)

p-JNK-183/185 (GTX635799, GeneTex)

SAPK/JNK (9252T, Cell Signaling Technology)

p-STAT3-705 (GTX118000, GeneTex)

p-STAT3-727 (AP0715, ABclonal)

STAT3 (A19566, ABclonal)

p-STAT6-641 (AP1390, ABclonal)

STAT6 (A19120, ABclonal)

IGF1R (A21984, ABclonal)

FGFR2 (A19051, ABclonal)

ARG1 (A4923, ABclonal)

β-actin (TA-09, ZSGB-BIO)

GAPDH (TA-08, ZSGB-BIO)

ESRα Rabbit pAb (A0296, ABclonal)^1–5^

**References**

1 Jia Z, Yang F, Liu X, Zhang X, Hu W, Sheng Z. The n-butanol fraction of the Xiao-Chai-Hu decoction alleviates the endocrine disturbance in the liver of mice exposed to lead. *Journal of Ethnopharmacology* 2021; **279**: 114381.

2 Zhao R, Feng D, Zhuang G, Liu Y, Chi S, Zhang J *et al.* Protein kinase CK2 participates in estrogen-mediated endothelial progenitor cell homing to endometriotic lesions through stromal cells in a stromal cell–derived factor-1– CXCR4-dependent manner. *Fertility and Sterility* 2020; **113**: 1067-1079.e5.

3 Lee SH, Shin HS, So YH, Lee DH, An B-S, Lee G-S *et al.* Maternal exposure to 4-tert-octylphenol causes alterations in the morphology and function of microglia in the offspring mouse brain. *Journal of Hazardous Materials* 2024; **480**: 136258.

4 Zhao X, Li X, Zhang W, Gao M, Zhong C, Zhang B *et al.* Integrated mRNA-seq and miRNA-seq analysis reveals miR-210a-5p regulates uterine aging in laying hens by targeting the RASL11B/Raf/MAPK pathway. *J Animal Sci Biotechnol* 2025; **16**: 129.

5 Dai Y, Yue N, Gong J, Liu C, Li Q, Zhou J *et al.* Development of cell-permeable peptide-based PROTACs targeting estrogen receptor α. *European Journal of Medicinal Chemistry* 2020; **187**: 111967.
